# Conditional Spike Transmission Mediated by Electrical Coupling Ensures Millisecond Precision-Correlated Activity among Interneurons In Vivo

**DOI:** 10.1016/j.neuron.2016.04.013

**Published:** 2016-05-18

**Authors:** Ingrid van Welie, Arnd Roth, Sara S.N. Ho, Shoji Komai, Michael Häusser

**Affiliations:** 1 Wolfson Institute for Biomedical Research and Department of Neuroscience, Physiology, and Pharmacology, University College London, Gower Street, London WC1E 6BT, UK; 2 Nara Institute of Science and Technology, 8916-5 Takayama, Ikoma, Nara 630-0192, Japan

## Abstract

Many GABAergic interneurons are electrically coupled and in vitro can display correlated activity with millisecond precision. However, the mechanisms underlying correlated activity between interneurons in vivo are unknown. Using dual patch-clamp recordings in vivo, we reveal that in the presence of spontaneous background synaptic activity, electrically coupled cerebellar Golgi cells exhibit robust millisecond precision-correlated activity which is enhanced by sensory stimulation. This precisely correlated activity results from the cooperative action of two mechanisms. First, electrical coupling ensures slow subthreshold membrane potential correlations by equalizing membrane potential fluctuations, such that coupled neurons tend to approach action potential threshold together. Second, fast spike-triggered spikelets transmitted through gap junctions conditionally trigger postjunctional spikes, depending on both neurons being close to threshold. Electrical coupling therefore controls the temporal precision and degree of both spontaneous and sensory-evoked correlated activity between interneurons, by the cooperative effects of shared synaptic depolarization and spikelet transmission.

## Highlights

• Double patch-clamp recordings from Golgi cells reveal millisecond synchrony in vivo
• Millisecond synchrony requires gap junctions and is enhanced by sensory stimuli
• Gap junctions drive synchrony via slow V_m_ equalization and fast spikelet transmission
• Modeling shows these findings can be generalized to any electrically coupled neurons

## Teaser

van Welie et al. show using double patch-clamp recordings that cerebellar Golgi cells display millisecond precise correlated activity in vivo, which is enhanced during sensory processing. Gap junctions mediate precise correlated activity via slow membrane potential equalization and fast spikelet transmission.

## Introduction

The cerebellum is thought to play a central role in governing the timing of movements. To understand how the cerebellum mediates temporally precise motor control, we need to understand the mechanisms underlying temporal coding in the cerebellar circuitry (Heck et al., 2013, Person and Raman, 2012b). Various types of correlated activity have been reported at the output stage of the cerebellar cortex, in the form of Purkinje cell complex spike synchrony (Bell and Kawasaki, 1972, Sasaki et al., 1989) and Purkinje cell simple spike synchrony (Heck et al., 2007). It has also been suggested that Purkinje cell simple spike synchrony will lead to time-locked activity of downstream target neurons in the deep cerebellar nuclei (Person and Raman, 2012a). However, to understand temporal coding regimes at the output stage of the cerebellar cortex, we must first determine how temporal codes are formed and transmitted at earlier stages in the circuit, starting with the temporal integration of mossy fiber inputs in the sensory input layer.

The granule cell layer forms the input layer of the cerebellar cortex and receives mossy fiber inputs conveying sensory and motor information that are integrated by two cell types: the excitatory granule cells and inhibitory Golgi cells. Golgi cells locally inhibit the excitatory granule cells and each other (Hull and Regehr, 2012). They have large receptive fields (Holtzman et al., 2006b, Tahon et al., 2005, Vos et al., 1999b) and have been reported to display a combination of responses to mossy fiber inputs: short-latency excitation alone, long-latency inhibition alone, or a combination of both (Holtzman et al., 2006a, Holtzman et al., 2006b, Prsa et al., 2009, Tahon et al., 2005, Tahon et al., 2011, Volny-Luraghi et al., 2002, Vos et al., 1999b). Golgi cell inhibition is traditionally thought to control the gain of granule cell excitation, thus ensuring sparse granule cell spiking (Marr, 1969), a hypothesis supported by the finding that, both in vitro and in vivo, tonic inhibition controls the gain of granule cell excitability (Chadderton et al., 2004, Duguid et al., 2012, Mitchell and Silver, 2003). However, Golgi-to-granule-cell connectivity patterns, which display a strong divergence of single Golgi cell axons onto many granule cells and the convergence of several Golgi cells onto individual granule cells, have prompted the hypothesis that Golgi cells also control the spatiotemporal patterning of granule cell activity (D’Angelo, 2008). This in turn can drive loose correlated activity of Golgi cells via the parallel fibers (Vos et al., 1999a). As such, it has been hypothesized that feedforward Golgi cell inhibition may provide a time-windowing function by limiting granule cell responsiveness to mossy fiber inputs (D’Angelo, 2008, D’Angelo and De Zeeuw, 2009).

In common with many other interneuron types (Connors and Long, 2004, Galarreta and Hestrin, 2001) in the mammalian brain, Golgi cells are electrically coupled (Dugué et al., 2009, Vervaeke et al., 2010). The contribution of electrical coupling to precisely correlated activity between interneurons, while often assumed to be important, is controversial, with experimental and theoretical work suggesting that both synchronization (Dugué et al., 2009, Galarreta and Hestrin, 1999, Gibson et al., 1999, Landisman et al., 2002, Long et al., 2005, Mann-Metzer and Yarom, 1999) and desynchronization (Chow and Kopell, 2000, Dugué et al., 2009, Ostojic et al., 2009, Pfeuty et al., 2003, Vervaeke et al., 2010) can occur depending on synaptic connectivity, spike shape, and intrinsic currents. Electrical coupling between Golgi cells has been proposed to synchronize Golgi cell networks at preferred oscillatory frequencies (Dugué et al., 2009) or to desynchronize oscillatory activity patterns under conditions of sparse mossy fiber excitation (Vervaeke et al., 2010). However, electrical coupling between Golgi cells, like electrical coupling between other interneuron types, has predominantly been studied in vitro, in isolation of spontaneous or sensory-evoked synaptic activity. The functional role of electrical coupling has not yet been directly studied in vivo, where circuitry is intact, background synaptic activity is high, and the synaptic inputs conveying sensory information can be stimulated directly.

We have therefore investigated the functional role of electrical coupling in the Golgi cell network in anaesthetized mice in vivo. Using targeted dual patch-clamp recordings from Golgi cells, we reveal that nearby Golgi cells display robust correlated activity with millisecond precision that is dependent on electrical coupling. Electrical coupling mediates this precisely correlated activity by the cooperative effect of two mechanisms: by equalization of the subthreshold membrane potential and by direct spike-to-spike transmission via spikelets. This precisely correlated activity is further enhanced when sensory stimuli are used to evoke spiking, even though spikes driven by sensory input display broad temporal variation due to the integration of sensory-evoked synaptic inputs. These results suggest a role for precisely correlated activity in Golgi cells in cerebellar function and provide a template for understanding correlated activity in electrically coupled interneuron networks across the mammalian brain.

## Results

### Neighboring Golgi Cells Display Correlated Activity with Millisecond Precision

**Figure 1:**
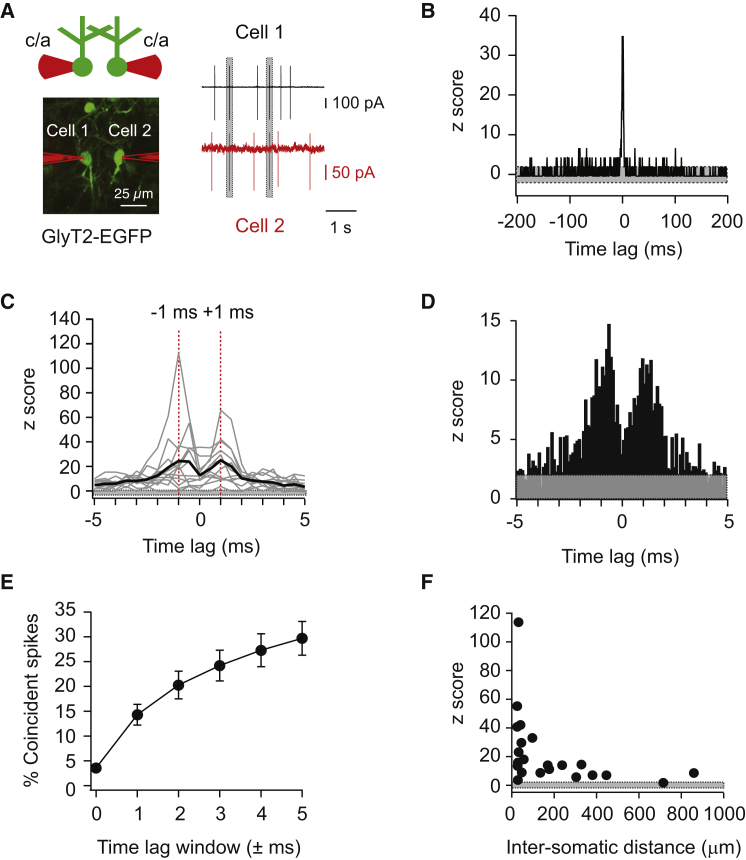
Golgi Cell Pairs Display Millisecond Precision-Correlated Activity (A) Recording configuration for measuring spike time correlations between two Golgi cells in vivo. Two EGFP-expressing Golgi cells (left) were targeted using two-photon guidance, and spike trains were recorded using dual loose patch cell-attached (c/a) recordings (right). Gray areas on spike trains indicate the occurrence of correlated spikes (< ±5 ms time lag). (B) The cross-correlogram (0.5 ms bins) computed from the example spike trains in (A) shows a large peak around 0 ms. (C) The cross-correlograms (0.5 ms bins) for all pairs recorded within 100 μm of each other (n = 12) display two peaks (red dotted lines) around 0 ms: at −1 ms and at +1 ms. Gray lines are cross-correlograms from individual pairs; the black trace is the mean of all 12 pairs. (D) Mean cross-correlogram as indicated in (C), but computed with 0.1 ms time bins revealing that the majority of spike time correlations occur between 0 and ±2 ms. (E) Computing the percentage of spikes that fall within distinct time lag windows across all pairs located <100 μm distance from each other reveals that 30% ± 3% of all spikes in WT mice are correlated within 5 ms of a spike in its paired neuron. (F) Recordings of Golgi cell pairs at various intersomatic distances indicated that the degree of correlation (defined as the maximal z score within a ±5 ms time lag window) decreases with distance. Gray areas in (B)–(D) and (F) indicate confidence interval (z scores between −2 and +2).

To establish whether Golgi cells exhibit correlated activity in vivo, two-photon guided targeted loose cell-attached recordings (Figure 1A) were made from Golgi cell pairs in mice anesthetized with ketamine/xylazine. Golgi cells fired spontaneously at 2.1 ± 0.3 Hz (mean ± SEM, n = 50 cells) without obvious rhythmicity (C.V. = 1.2 ± 0.1). This spontaneous activity is driven in part by synaptic input, as voltage-clamp recordings from Golgi cells showed a high level of continuous background synaptic activity, with EPSCs and IPSCs occurring at rates of 73 ± 12 and 64 ± 11 Hz, respectively (see Figures S1A–S1D, available online). Normalized cross-correlations of spike times in Golgi cell pairs were computed by subtracting the mean cross-correlogram of 30 shuffled spike trains for each channel from the raw cross-correlogram and then dividing by the SD of the mean shuffled cross-correlogram. This normalization method controls for chance effects and normalizes for changes in firing rate between cells and pairs. The resulting normalized cross-correlograms exhibited prominent peaks around 0 ms time lag (Figures 1B and 1C). In most pairs, two discrete peaks were present—one at −1 ms and one at +1 ms (Figure 1C)—suggesting that spikes can either precede or follow a spike from a neighboring Golgi cell with millisecond precision. Histograms with 0.1 ms time bins showed that the majority of correlated activity occurred between 0 and ±2 ms (Figure 1D). To further quantify the precision of correlated activity, we determined the percentage of correlated spikes that occurred within time windows ranging between 0 to 5 ms. This revealed that 30% ± 3% of all spikes in a spike train occurred within ±5 ms of a spike from its paired neuron (n = 12 pairs, Figure 1E). Precisely correlated activity was dependent on intersomatic distance, with the strongest correlated activity found at intersomatic distances of less than 100 μm (Figure 1F). There was no significant relationship between the degree of correlated activity and the plane of orientation of Golgi cell pairs (Figures S1E and S1F), indicating that proximity alone determines the degree of millisecond precision-correlated activity, rather than activation by shared parallel fiber inputs (Volny-Luraghi et al., 2002, Vos et al., 1999a).

### Electrical Coupling Is Essential for Precisely Correlated Activity

**Figure 2:**
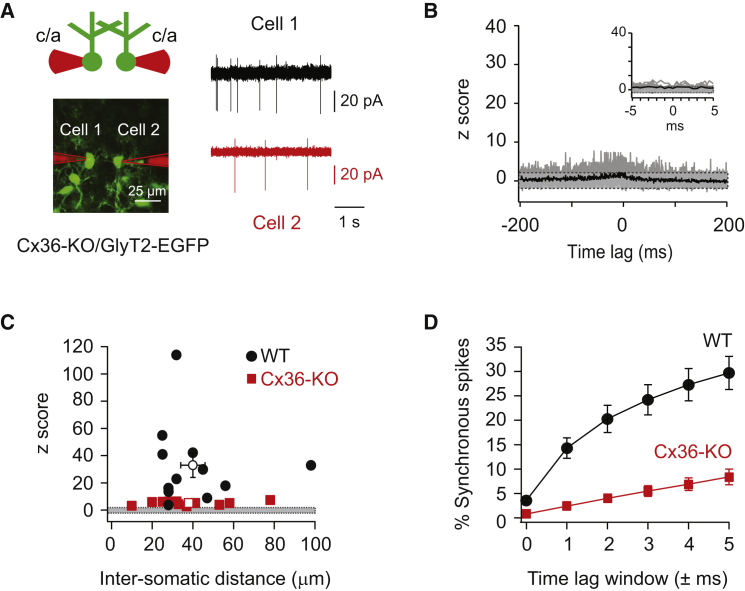
Precisely Correlated Activity Is Lacking in Cx36-KO Mice (A) Recording configuration for measuring correlated activity between Golgi cells in a mouse line in which connexin36 is knocked out and glycinergic neurons express EGFP (Cx36-KO/GlyT2-EGFP, left). Dual loose-patch recordings were performed to determine spike timing in two neighboring Golgi cells (right). (B) The cross-correlograms of all pairs recorded in Cx36-KO/GlyT2-EGFP mice lack a central peak around zero, indicating a lack of strong and precisely correlated activity. Gray lines are cross-correlograms from individual pairs; the black trace is the mean over all 12 pairs. The inset (right) displays the individual (gray lines) and mean (black line) cross-correlograms (0.5 ms bins) from −5 to +5 ms for all pairs (n = 12). (C) Summary of maximal z scores determined from cross-correlograms of spike trains between Golgi cells pairs recorded with <100 μm intersomatic distance reveal strongly reduced degrees of correlated activity in Cx36-KO animals (mean = 5.0 ± 0.4, n = 12; red solid squares) compared to pairs in wild-type animals (mean = 33.1 ± 8.5, n = 12; black solid circles, p = 0.001). (D) Computing the percentage of spikes within time lag windows between 0 and 5 ms across spike trains in all pairs reveals that 30% ± 3% of all spikes in WT mice are correlated within 5 ms. Only 8% ± 2% of all spikes across all spike trains in pairs in the Cx36-KO/GlyT2-EGFP mice displayed correlated activity with similar precision (n = 12, p < 0.0001). Gray areas in (B) and (C) indicate the confidence interval (z scores between −2 and +2).

To test the contribution of electrical coupling to precisely correlated Golgi cell activity we crossed connexin36 knockout (Cx36-KO) mice with GlyT2-EGFP mice (see Supplemental Experimental Procedures). Connexin36 is both necessary and sufficient for mediating electrical coupling between Golgi cells (Vervaeke et al., 2010). We verified that the connexin36 gene was disrupted in the crossed mouse line and confirmed the results with immunocytochemistry (Experimental Procedures; Figures S2A and S2B). Baseline firing rates and irregularity of spiking in the Cx36-KO/GlyT2-EGFP mice were similar to those in wild-type GlyT2 mice (2.7 ± 0.5 Hz; C.V. = 1.2 ± 0.2, n = 19; p = 0.06 and p = 0.13, Figure S2C). Strikingly, though, paired recordings (Figure 2A) revealed a lack of precisely correlated activity in the Cx36-KO/GlyT2-EGFP mice: cross-correlations of spike times lacked peaks around 0 ms time lag (Figure 2B), and the mean maximal z score in the ± 5 ms time lag window around zero (5.0 ± 0.4, n = 12 pairs) was significantly reduced compared to the mean maximal z score in wild-type GlyT2-mice (33.1 ± 8.5, n = 12 pairs, p = 0.0002, Figures 2B and 2C). In Cx36-KO mice, only 8% ± 2% of all spikes on a given spike train occurred within ±5 ms of spikes from its paired neighbor, significantly fewer than in wild-type mice (30% ± 3%, p < 0.0001, n = 12, Figure 2D). These results indicate that strong and precisely correlated activity requires functional electrical coupling via gap junctions between Golgi cells.

### Slow Subthreshold Membrane Potential Correlations Mediated by Electrical Coupling

**Figure 3:**
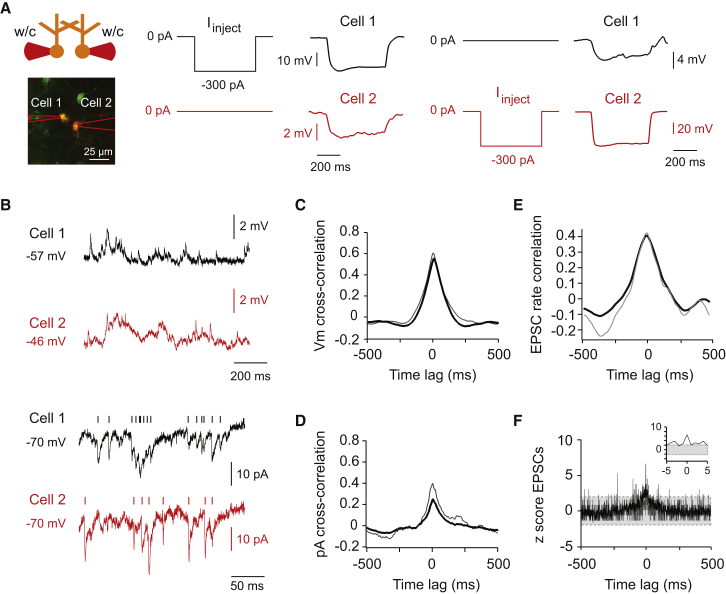
Dual Recordings Reveal Slow Subthreshold Correlations in Membrane Potential between Coupled Cells (A) Recording configuration for dual whole-cell recordings between Golgi cells (left). Two nearby (<100 μm) Golgi cells were targeted, and membrane potentials or synaptic currents were recorded using dual whole-cell (w/c) recordings in current- and/or voltage-clamp mode. Consecutive hyperpolarizing current injections in one of two cells were used to determine electrical coupling between the two Golgi cells (middle and right). The coupling coefficient for this pair was 9%, averaged over both directions. (B) Example traces of a dual whole-cell recording in a coupled Golgi cell pair. Membrane potential fluctuations were recorded in current-clamp mode (top traces), and membrane currents were recorded in voltage-clamp mode (bottom traces). The occurrences of identified individual EPSCs are indicated above the voltage-clamp traces (vertical lines). (C) Cross-correlograms (on a scale of −1 to +1) computed from membrane voltage fluctuations revealed slow membrane potential correlations. The gray trace is the cross-correlation from the example traces shown in (B), and the black trace is the average cross-correlation across five pairs. (D) Cross-correlograms computed from membrane current fluctuations also revealed a slow but less substantial correlation compared to the cross-correlation of voltage fluctuations. The gray trace is the cross-correlation from the example traces shown in (B), and the black trace is the average cross-correlation across three pairs. (E) Cross-correlations of EPSC rates computed by counting synaptic event times in 128 ms bins. The gray trace is the cross-correlation of EPSC rates in a single pair. The black trace is the mean cross-correlation across three pairs of coupled neurons. (F) The mean normalized cross-correlogram (1 ms bins) of the event times of individual EPSCs displays a small significant (z score of >2) peak around 0 time lag (inset). The gray trace is the cross-correlation from the example traces shown in (B), and the black trace is the average cross-correlation across three pairs. Gray area indicates the confidence interval (z scores between −2 and +2).

To determine the mechanisms by which electrical coupling controls precisely correlated activity in vivo, dual whole-cell recordings were made from neighboring Golgi cells (Figure 3A). Most neighboring Golgi cells exhibited direct bidirectional electrical coupling in vivo, with a coupling coefficient of 5% ± 2% (n = 8; four pairs with detectable coupling out of five pairs). Spontaneous membrane potential fluctuations in coupled cells (Figure 3B, upper traces) exhibited slow positive membrane potential correlations (mean peak of 0.6 ± 0.1 on a scale of −1 to +1, and mean width between the negative inflection points of the cross-correlation peak = 474 ± 71 ms, or ∼2 Hz, n = 5, Figure 3C), which were absent in a noncoupled pair (Figure S3). In voltage clamp mode, spontaneous current fluctuations (Figure 3B, bottom traces) exhibited slow correlations on a similar timescale to the spontaneous voltage fluctuations (Figure 3D), but with a less prominent peak than seen for voltage fluctuations (mean of 0.3 ± 0.1, n = 3). In voltage clamp, synaptic currents are largely isolated from the effects of electrical coupling between the recorded neurons, as no current will flow between gap junctions as a result of differences in membrane potential between the two cells. The reduced correlation seen in voltage clamp therefore suggests that electrical coupling strongly contributes to the substantial cross-correlation of membrane voltage observed in current clamp. Given that gap junctions between Golgi cells act as low-pass filters with a cut-off frequency of ∼10 Hz (Dugué et al., 2009) and that the degree of correlated activity is strongly reduced when isolating synaptic inputs from the effects of electrical coupling in voltage clamp mode, we conclude that electrical coupling plays a prominent role in mediating slow membrane potential correlations between Golgi cells.

The raw data in both current and voltage clamp (Figure 3B) show that the rate of EPSPs/EPSCs fluctuates and that some of these fluctuations occur at the same time in both neurons. To quantify this, we detected EPSCs in both cells (Figure 3B, bottom traces) and determined the cross-correlation of the rate of EPSCs by binning EPSC coincidences in 128 ms bins in pairs of Golgi cells. EPSCs showed a peak EPSC rate cross-correlation of 0.4 ± 0.1 (Figure 3E, n = 3), suggesting that the EPSC rate is comodulated between two coupled neurons over the timescale of ∼100 ms. This slow comodulation of EPSC rate is likely to contribute to the slow correlations of input current measured in voltage clamp shown in Figure 3D, and in turn to the slow membrane potential correlations observed during voltage recording (Figure 3C). The slow membrane potential correlations resulting from both membrane potential equalization via coupling and slow comodulation of EPSC rates increase the probability that coupled neurons depolarize together toward action potential threshold.

We next computed cross-correlograms of individual EPSC event times and determined the percentage of precisely synchronous EPSCs between coupled neurons. Only a small fraction of the EPSCs (4% ± 1%, n = 3) computed from cross-correlations were precisely synchronous (defined as occurring within ±0.5 ms), as reflected in the small peak (z score of 6 ± 4) in the normalized cross-correlogram at 0 ms (Figure 3F). These results indicate that precisely synchronous or common EPSCs are rare, with the slow tails in the EPSC cross-correlogram reflecting overall periods of EPSC rate correlation at a slower timescale (compare with Figure 3E). The fact that precisely synchronous EPSCs are rare suggests that they are unlikely to drive the majority of spikes that exhibit correlations with millisecond precision.

### Spikes Trigger Depolarizing Spikelets via Gap Junctions

**Figure 4:**
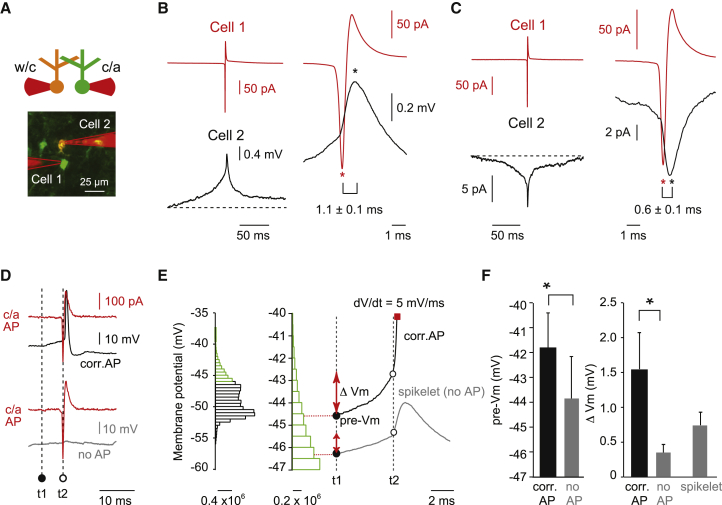
Triggering of Correlated Spikes by Spikelets Is State Dependent (A) Recording configuration for measuring the effect of single action potentials on spike timing. Two nearby (<100 μm) Golgi cells were targeted using two-photon guidance. One cell was recorded in cell-attached (c/a) mode (Cell 1), and the other cell was recorded in whole-cell (w/c) current-clamp mode (Cell 2). (B) Dual cell-attached and whole-cell recordings during spontaneous correlated activity allowed us to characterize spike-triggered voltages or so called “spikelets” in postjunctional Golgi cells. Cell-attached (c/a) spikes (top red trace, left) from Cell 1 were used to characterize the mean spike-triggered voltage (lower black trace, left) in Cell 2. A fast spike-triggered event could be identified riding on top of a broader depolarization that outlasted the spike. On the right, an overlay of the mean cell-attached spike and the whole-cell spike-triggered voltage is shown. The fast spike-triggered “spikelet” always occurred after the peak of the cell-attached spike, and the mean time lag between the two events was 1.1 ± 0.1 ms. Data represent mean ± SEM of nine pairs. (C) Same parameters as in (A) and (B), but for spike-triggered currents recorded in voltage-clamp mode. The mean time lag between the two events was 0.6 ± 0.1 ms. Data represent mean ± SEM of six pairs. (D) The membrane potential in Cell 2 in the 5 ms prior (t2 − t1) to the induction of precisely correlated (<±5 ms) spikes (black trace) or during failures (gray trace) was analyzed using spike-triggered averaging of the cell-attached spikes in Cell 1 (red trace). (E) Example recording showing on the left the membrane potential distribution (in counts of membrane potential data points recorded) of Cell 2, with the upper most depolarized range indicated in green. On the right, the mean V_m_ trajectories 5 ms prior (t1) to the peak of the cell-attached spike (t2) in correlated spikes (black trace) and during failures (gray trace) are mapped onto the depolarized subset of the membrane potential distribution (middle), which shows that correlated spikes occur when the membrane potential is most depolarized. In the case of failures (gray trace), a spikelet occurs immediately after t2. Pre-V_m_ is absolute membrane potential value at t1, and ΔV_m_ is the voltage difference between t1 and t2 (t1 = t2 − 5 ms). Red square marker indicates action potential threshold for this cell, defined as the point where dV/dt exceeds 5 mV/ms. (F) Mean data for pre-V_m_ values (p = 0.01), ΔV_m_ (p = 0.02), and spikelet amplitude computed across n = 5 pairs.

Slow subthreshold membrane potential correlations mediated by coupling may be permissive for correlated activity, but they do not explain the millisecond precision of the correlated activity we observed. We therefore set out to test whether transmission of a spike via gap junctions and the resulting “spikelet” currents can trigger millisecond precision correlated activity. First, we examined the amplitude of spikelet currents and potentials by computing spike-triggered averages in simultaneous whole-cell and cell-attached recordings (Figure 4A). This revealed that prejunctional action potentials were associated with depolarizing junction potentials (0.4 ± 0.2 mV, n = 9; Figure 4B) and currents (8 ± 2 pA, n = 6; Figure 4C) in the postjunctional cells. These spikelet voltages and currents followed the spikes in the coupled cell with a mean peak latency of 1.1 ± 0.1 ms and 0.6 ± 0.1 ms, respectively. Importantly, spikelets were absent (mean depolarization = 0.05 ± 0.01 mV in first 1.5 ms following cell-attached spike versus 0.4 ± 0.2 mV, p = 0.02) in pairs that did not appear to be electrically coupled given that they lacked significant precisely correlated activity (mean maximal z score = 5 ± 1, n = 3, Figure S4).

Spikelet potentials are small, and thus, to determine how spike-to-spike transmission across gap junctions may depend on the membrane potential in the postjunctional cell, we analyzed the membrane potential 5 ms prior to a correlated spike triggered by a cell-attached spike in its coupled neighbor and compared it to the membrane potential on those occasions in which a correlated spike failed to be induced (Figure 4D). This revealed that correlated spikes were triggered when the absolute membrane potential was more depolarized and closer to threshold (pre-*V*_m_, –41.8 ± 1.4 mV for correlated spikes and –43.9 ± 1.7 mV for noncorrelated spikes, n = 5, p = 0.01, Figures 4E and 4F). Furthermore, the membrane potential depolarized more in the 5 ms prior to a correlated spike compared to when spike-to-spike transmission failed (Δ*V*_m_, 1.5 ± 0.4 mV for correlated spikes and 0.5 ± 0.1 mV for noncorrelated spikes, n = 5, p = 0.02, Figures 4E and 4F). Finally, even in episodes where no spikes were induced, the membrane potentials were on a depolarizing trajectory (Δ*V*_m_ was positive, Figures 4E and 4F), suggesting that both coupled neurons are depolarizing prior to the generation of a spike in one of the two cells, as predicted by the slow subthreshold membrane potential correlations (Figure 3). These data suggest that spike-to-spike transmission via gap junction-mediated spikelets in vivo occurs when the membrane potential in the postjunctional cell is on a depolarizing trajectory and close to threshold.

### Triggered Spikelets Can Drive Precisely Correlated Postjunctional Spikes

**Figure 5:**
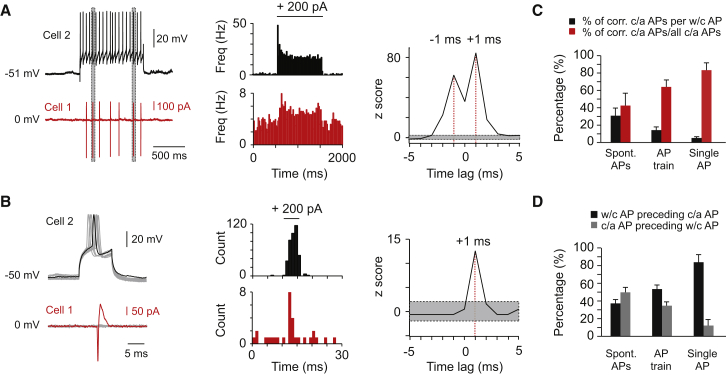
Induced Spikelets Can Drive Spike-to-Spike Transmission (A) Example traces (left) from an experiment (same experimental configuration as in Figure 4) in which a (1 s) train of action potentials was induced in Cell 2 and the effect on spike timing was assessed in Cell 1. The poststimulus time histograms (PSTH, 20 ms bin, middle) of spiking summarizing results across multiple trials indicate that the firing rate was transiently increased in Cell 1 during the time that spikes were induced in Cell 2. Gray bars (left) indicate correlated spikes (<±5 ms). The cross-correlogram (1 ms bins, right) shows two peaks (red dotted lines) at −1 ms and +1 ms, similar to spontaneous correlated activity. (B) Example traces (left) from an experiment where only a single action potential was induced in Cell 2. Across multiple trials, these single action potentials were capable of inducing correlated spikes in the coupled cell, despite a high failure rate (PSTHs, 1 ms bin, middle). The timing of correlated spikes in Cell 1 (±5 ms time lag window) also displayed millisecond precision, but spikes in Cell 1 always followed the spikes in Cell 2, resulting in a single peak at +1 ms (right). (C) Summary graph displaying the percentage of correlated spikes (<±5 ms time lag) observed under three conditions: during spontaneous action potentials, during a stimulus induced train of action potentials, and during stimulus induced single action potentials. Black bars indicate the percentage of spikes in Cell 2 that induced a correlated spike in Cell 1. Red bars indicate the percentage of correlated spikes in Cell 1 of all spikes in Cell 1. Data represent mean ± SEM from six pairs. (D) Summary graph displaying the symmetry of correlated spikes (±5 ms time window) across the three different experimental conditions. Black bars indicate the percentage of spikes in Cell 2 that precede spikes in Cell 1. Gray bars indicate the percentage of spikes in Cell 1 that precede the spikes in Cell 2. Data represent mean ± SEM from six pairs.

To test directly whether spikelets can trigger postjunctional spikes, we triggered spikes by current injection in one cell of a coupled pair (recorded in whole-cell mode) while recording the spiking of the other cell (in cell-attached mode). A train of spikes in the prejunctional cell led to a robust increase in spiking of the postjunctional cell (166% ± 82% increase in firing rate, n = 6; Figure 5A, left and middle). Cross-correlograms of pre- and postjunctional spikes revealed that spikes occurred with millisecond (±1 ms) precision (Figure 5A, right) comparable to spontaneous correlated spiking (Figure 1). To separate the effects of prejunctional membrane depolarization and the triggered action potentials, we next examined spike-to-spike transmission across gap junctions by using brief current pulses to trigger single spikes in the prejunctional cells (Figure 5B, left). Single induced action potentials were able to trigger precisely timed spikes in the postjunctional cells on 5% of the trials (Figure 5B, middle). The postjunctional spikes were driven with millisecond precision by the prejunctional spike, with a single peak in the cross-correlograms of induced whole-cell spikes and spikes in its coupled neighbor at +1 ms (Figure 5B, right).

Spike-to-spike transmission across the electrical junction was state dependent, however, as shown by the probability of spike transmission in different conditions. Spikes triggered by current injection in one prejunctional cell had a lower transmission probability than spontaneous spikes (Figure 5C, black bars), presumably because spikes were triggered at random times, and could therefore occur when the postjunctional cells may be far from threshold. In contrast, precisely correlated cell-attached spikes as a percentage of all cell-attached spikes increased during spike-injection experiments compared to spontaneous spiking conditions (Figure 5C, red bars). Similarly, the symmetry in spike coupling switched from being roughly 50:50 under spontaneous spiking conditions, to a situation where the triggered spikes nearly always preceded the postjunctional spikes (Figure 5D, black and gray bars). Notably, spike-to-spike transmission was unsuccessful in 3 pairs that lacked spontaneous precisely correlated activity (mean maximal z score = 5 ± 1, n = 3, Figure S5) and spikelets (Figure S4), confirming that electrical coupling is required for reliable spike-to-spike transmission. Together, these findings suggest the following mechanism: only if a combination of EPSC rate correlation and equalization of membrane potential via electrical coupling brings two coupled cells close to threshold, then a spike in one cell will lead to a spike in the other cell with a millisecond delay via a transmitted spikelet.

### Cooperative Effect of Subthreshold Membrane Potential Equalization and Spikelets Drives Precisely Correlated Activity

**Figure 6:**
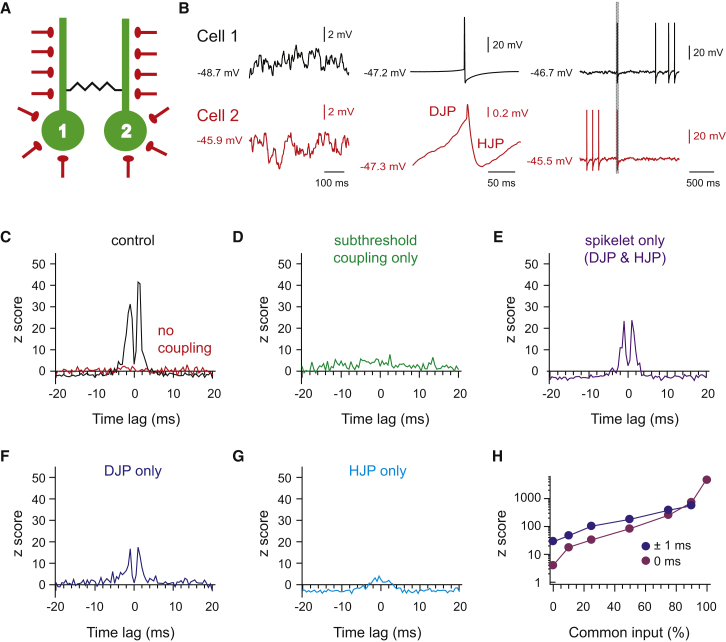
Cooperative Effect of Subthreshold Membrane Potential Correlations and Spikelet Transmission Drives Precisely Correlated Activity (A) Schematic overview of the computational model in which two neurons are electrically coupled and both receive random synaptic input, 4% of which is common to both neurons (see Supplemental Experimental Procedures). (B) Left panel, subthreshold membrane potential traces recorded at the soma. Center panel, an action potential was generated by membrane potential fluctuations crossing threshold (top) resulting in a transmitted spikelet potential (bottom), consisting of a depolarizing junction potential (DJP) and hyperpolarizing junction potential (HJP). In the model we were able to selectively turn off subthreshold membrane potential correlation due to coupling, and/or the spikelet potentials. Right panel, example traces of spiking in the two coupled model neurons. Gray bar indicates the occurrence of correlated spikes (<5 ms time lag). (C) Cross-correlograms of spike timing in the two coupled neurons under control conditions and with gap junction coupling entirely off. (D) Cross-correlogram with only subthreshold membrane potential correlation due to electrical coupling on, and with the transmission of spikelets off. (E) Cross-correlogram with only spikelet transmission on, and no electrical coupling at subthreshold membrane potentials. (F) Cross-correlogram with only DJP transmission on. (G) Cross-correlogram with only HJP transmission on. (H) Degree of correlated activity as a function of increasing degrees of precisely synchronous common input. Blue line is the average z score over the two peaks in the cross-correlograms at ±1 ms. The purple line is the z score at the 0 ms time bin representing the degree of correlated activity as a function of precisely synchronous common synaptic input.

To develop a more quantitative understanding of how synaptic input, subthreshold membrane potential correlation, and spikelets combine and interact to generate millisecond precision correlated activity, we constructed a generalized mathematical model of two electrically coupled neurons (Figures 6A and 6B). In this model, both neurons received a constant rate of in vivo-like EPSCs and IPSCs (Figure 6B; see also Supplemental Experimental Procedures) and the degree of precisely synchronous common input was set to 4%, as determined experimentally. Precisely synchronous common and independent inputs had equal amplitudes, which were set to be equivalent to those observed in the experimental data (Figure S1D; see also Supplemental Experimental Procedures). The model enabled us to turn off electrical coupling entirely, or to selectively turn off the subthreshold membrane potential correlation due to coupling and/or the spikelet. Furthermore, it allowed us to specifically turn off the depolarizing junction potential (DJP) or the hyperpolarizing junction potential (HJP) in each spikelet (Figure 6B). Under baseline conditions, spiking in these electrically coupled neurons displayed correlated activity with peaks in the cross-correlogram at ±1 ms (mean z score at ±1 ms = 36, z score at 0 ms = 3; Figures 6B and 6C, black trace), comparable to the experimentally observed correlated activity in Golgi cells in vivo. Turning off electrical coupling resulted in minimal correlated spiking (z score at 0 ms = 1; Figure 6C, red trace), qualitatively consistent with the experimental observations using the Cx36-KO mice (Figure 2B), and the idea that correlated spiking is not driven primarily by precisely synchronous EPSCs. Turning off the transmission of the spikelet while leaving subthreshold correlations due to coupling intact increased the degree of correlated activity somewhat (z score at 0 ms = 2, Figure 6D), but did not result in precisely correlated activity with peaks at ±1 ms. Turning off subthreshold correlations while turning on spikelet transmission, however, resulted in the emergence of ±1 ms peaks in the cross-correlogram, albeit with z scores lower compared to control (mean z score at ±1 ms = 24, z score at 0 ms = 3, Figure 6E). Together, these results suggest that subthreshold membrane potential correlations and spikelets cooperate to produce robust correlated activity with millisecond precision as seen experimentally.

Dissecting the effects of the DJP and HJP of the spikelets revealed that the DJP on its own causes the ±1 ms peaks (mean z score at ±1 ms = 17, z score at 0 ms = 1, Figure 6F), while the HJP on its own does not result in peaks at ± 1 ms, but in a peak at 0 ms with z score = 2 (Figure 6G), a value which is comparable to the z score at 0 ms in the case of subthreshold coupling only. The HJP also suppresses correlated firing, decreasing the cross-correlations beyond ±2 ms and rendering the z scores negative (Figure 6G). Next, we set out to determine how increasing levels of precisely synchronous common synaptic input affects correlated activity mediated by electrical coupling. Increasing the degree of precisely synchronous input enhances the peak at 0 ms, but only starts dominating spike timing over spikelet-induced peaks at ±1 ms when the degree of precisely synchronous input is >80% (Figures 6H and S6).

The prejunctional spike used in the model was based on the spike shape observed experimentally in Golgi cells, with a relatively long-lasting and deep AHP (17 mV amplitude and a duration of 14 ms defined as the full width at half-maximum, or FWHM, of the AHP). Given that the AHP has been predicted to cause desynchronization of neurons (Vervaeke et al., 2010), we modified the model to use a spike shape with a smaller and faster AHP (8 mV and 9 ms FWHM duration), more similar to cortical interneuron spikes. The degree of correlation as a function of differing degrees of precisely synchronous common input was qualitatively similar with both spike shapes (Figures S6A and S6B), indicating that millisecond precision correlated activity is reliably generated for a range of AHP amplitudes and durations. Finally, the model allowed us to study correlated spiking as a function of degree of precisely synchronous common input in the complete absence of gap junction coupling. This showed that for nonzero degrees of common input, correlated activity displays peaks at 0 ms, indicating perfect synchrony in spike timing due to synaptic inputs alone (Figure S6C). This result indicates that peaks at ±1 ms in spike time cross-correlations are caused by electrical coupling, not common synaptic inputs, and are thus a useful signature of electrical coupling in cells exhibiting correlated activity.

### Sensory Stimuli Induce Spikes via Integration of Bursts of EPSCs

**Figure 7:**
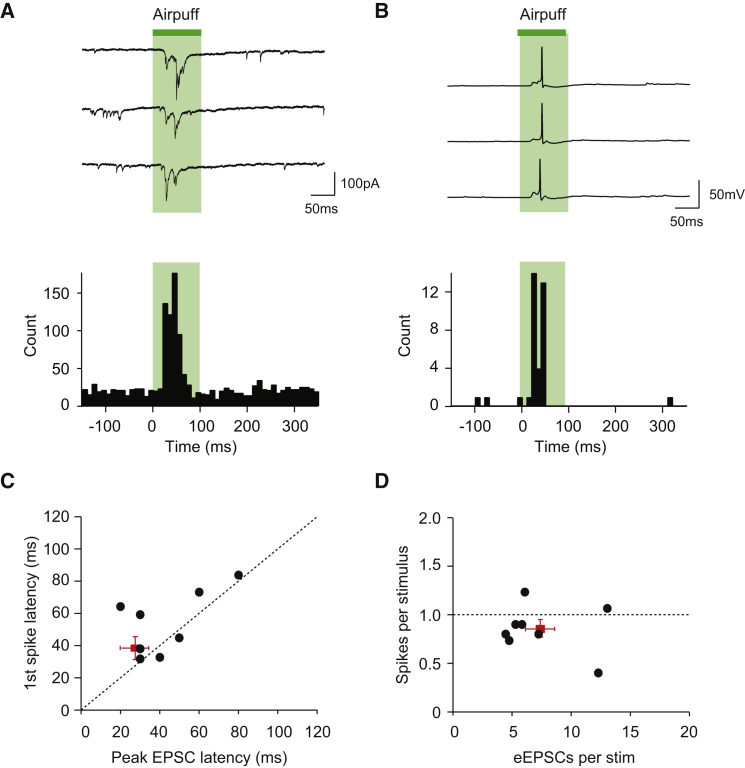
Sensory-Evoked Synaptic Integration and Spike Induction in Golgi Cells (A) Whole-cell patch clamp recording from a single Golgi cell showing that sensory stimulation of the peri-oral region and/or whisker pad evokes a burst of excitatory postsynaptic currents (EPSCs) (green area indicates duration of airpuff). Three consecutive trials and the peristimulus time histogram (PSTH, 10 ms bins) across trials are shown. (B) Spikes evoked by the same stimulus in the same cell as in (A). Three consecutive trials are shown, and one sensory-evoked spike is seen per trial in this cell. The PSTH of all evoked spikes across trials (10 ms bins) is shown below the traces. (C) Plotting the spike latency against the peak latency of the burst of EPSCs across cells reveals that Golgi cells spike after the burst of evoked EPSCs reaches its peak. Red square indicates mean ± SEM (n = 8 cells). (D) Golgi cells on average produce one spike per sensory stimulation, in response to a ranging number of evoked EPSCs (eEPSCs) within a burst. Red square indicates mean ± SEM (n = 8 cells).

Finally, we determined the degree and temporal characteristics of correlated activity in coupled Golgi cells in response to sensory stimulation. Since mossy fiber terminals conveying sensory stimuli terminate profusely in the granule cell layer and may simultaneously activate many coupled Golgi cells, precisely correlated activity between Golgi cells may be enhanced by a sensory stimulus. Alternatively, sensory stimuli might desynchronize or lessen the degree of correlated activity (Vervaeke et al., 2010). Furthermore, the temporal precision of correlated activity may broaden as a result of heterogeneous sensory-evoked responses in individual cells. We first characterized the synaptic inputs driven by sensory stimulation in single Golgi cells using whole-cell voltage clamp recordings. Sensory stimulation induced a burst of high-frequency excitatory inputs in Golgi cells, with a mean latency to the peak of the EPSC burst of 28 ± 7 ms (n = 8). In current-clamp recordings, this sensory-evoked synaptic input triggered single action potentials in most trials, with a mean latency of 35 ± 6 ms (n = 10, Figures 7A and 7B). The latencies of the peak of the EPSC burst and the resulting spikes in the same cells were highly correlated (Figure 7C), with the number of individual EPSCs leading to a spike ranging from 4 to 14 (Figure 7D). Thus, under these experimental conditions, Golgi cells need to integrate a minimum of 4–14 individual EPSCs before reaching action potential threshold.

### Enhanced Correlated Activity with Millisecond Precision during Sensory Stimulation

**Figure 8:**
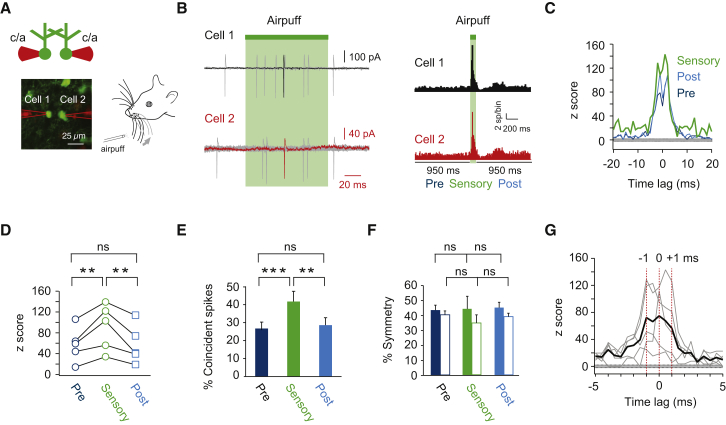
Enhanced Correlated Activity with Millisecond Precision during Sensory Stimulation (A) Recording configuration for measuring sensory evoked correlated activity between Golgi cells in GlyT2-EGFP mice. Two nearby Golgi cells were targeted and spike trains were recorded using dual loose cell-attached (c/a) recordings (left). Sensory stimulation was provided by an air puff to the peri-oral region and/or whisker pad (right). (B) Ten overlaid example traces on the left reveal the occurrence of sensory-induced spikes in response to the air-puff. One trial is highlighted (in black and red) on both spike trains. Green bars indicate the duration of the air puff (100 ms). Histograms on the right summarizing the results across all trials reveal clear sensory responses in both cells in this pair. The degree of correlated activity was computed 950 ms preceding the sensory stimulus (Pre), during the 100 ms stimulus (Sensory), and in the 950 ms following the stimulus (Post). (C) Cross-correlograms of the example pair shown in (B). The data indicate that the sensory-evoked stimulus significantly enhances prestimulus correlated activity, which returns to baseline levels during the poststimulus time period. (D) Summary graph of z score values before, during, and after sensory stimuli across all pairs (n = 5). (E) Summary bar graph displaying the percentage of correlated spikes before, during, and after sensory stimuli across all pairs (n = 5). (F) Summary bar graph displaying the symmetry of correlated spikes before, during, and after sensory stimuli across all pairs (n = 5). Solid bars reflect Cell 1 preceding Cell 2, and open bars reflect Cell 2 preceding Cell 1. (G) Individual (gray lines) and mean (black line) cross-correlograms (0.5 ms bins) of sensory evoked correlated activity indicating a multipeaked structure around zero time lag with peaks at −1, 0, and +1 ms (red lines).

In simultaneous recordings from pairs of Golgi cells, sensory stimulation triggered spiking in both cells (latency of 32 ± 2 ms; n = 5 pairs, Figures 8A and 8B), resulting in correlated activity that was more pronounced than spontaneous correlated activity in the same pairs (mean z score 91 ± 20 versus 57 ± 15 prestimulus, p < 0.01, and 57 ± 17 poststimulus, p < 0.01, n = 5; Figures 8C and 8D). Accordingly, the percentage of correlated spikes (defined as within ±5 ms) of all spikes was higher during sensory stimulation (42% ± 6%, n = 5) compared to that during spontaneous correlated activity (27% ± 4% prestimulus, p < 0.001 and 29% ± 4% poststimulus, p < 0.01, respectively, n = 5; Figure 8E). The bidirectional symmetry of correlated spiking between coupled Golgi cells did not change during sensory evoked stimuli (p = 0.96 and p = 0.33 for both directions, Figure 8F). Given that dominant inputs to only one cell would cause asymmetry due to the fact that electrical coupling is bidirectional, this suggests that both cells were driven approximately equally by the sensory stimulus.

The sensory-evoked cross-correlograms exhibited peaks at −1, 0, and +1 ms (Figures 8C and 8G), as in spontaneous spiking, suggesting that while the peak at 0 ms is larger during sensory stimuli, electrical coupling still strongly determines spike timing during sensory stimuli. However, we also observed a broader foot in the cross-correlogram of the sensory-evoked spikes, representing enhanced correlations at longer time lags (Figure S7A). To determine whether the variability of evoked spiking in individual cells underlies this increase in slower time correlations, we shuffled spike times across sensory trials which revealed broad peaks around zero (Figures S7B and S7D), similar to the autocorrelations of spike times across trials for each cell in a pair (Figure S7C). Across pairs, these shuffled cross-correlograms revealed broad correlations lacking sharp peaks around 0 ms, suggesting that the main effect of sensory stimuli is to enhance temporal correlations at longer time lags (Figure S7D). Together, these data suggest that sensory stimuli enhance the degree of correlated spiking between pairs of Golgi cells and enhance temporal correlations at longer time lags. Nevertheless, the temporal precision of the correlated activity that is ensured by electrical coupling still dominates the spike timing of coupled Golgi cells.

## Discussion

We report the first demonstration of correlated activity with millisecond precision in an identified electrically coupled interneuron network in vivo. Electrical coupling is essential for the temporal precision of this correlated activity, and acts in a dual, cooperative manner: by equalizing slow subthreshold membrane potential depolarizations and by transmitting fast depolarizing spikelet currents. Sensory stimuli evoke bursts of synaptic inputs that evoke spikes with variable timing across trials in individual cells, but they enhance the degree of precisely correlated activity. Since many interneurons in the mammalian brain are electrically coupled (Connors and Long, 2004, Galarreta and Hestrin, 2001), our results provide a mechanistic understanding of how electrical coupling can orchestrate millisecond-scale correlated activity under conditions of spontaneous and sensory evoked synaptic activity.

### Electrical Coupling Employs Two Cooperative Mechanisms to Ensure Precisely Correlated Activity of Golgi Cell Firing In Vivo

Correlated activity with millisecond precision has not previously been reported in the Golgi cell population in vivo because of the difficulty of recording unambiguously from neighboring Golgi cells in the intact brain. Previous findings of weaker, less precisely correlated activity (half-width of the cross-correlogram peak of ∼29 ms) among Golgi cells (Vos et al., 1999a), presumably mediated by common parallel fiber input, were made with electrode spacings 300–2,100 μm apart, well beyond the ∼200 μm span of the Golgi cell dendritic tree that defines the spatial dimension of the precisely correlated activity that we have observed. Our dual whole-cell recordings between coupled Golgi cells demonstrate that gap junctional coupling enables precisely correlated activity via the cooperative effect of two mechanisms. First, electrical coupling helps to equalize subthreshold membrane potentials, effectively allowing the neurons to share synaptic input (Vervaeke et al., 2012) by transmitting slow membrane potential fluctuations across the junction. Second, when both cells are driven close to threshold, then a spike triggered in one cell is able to trigger a spike in a coupled cell by transmission of a spikelet across the gap junction. These two mechanisms work together to ensure precisely correlated activity: the efficacy of the spike-to-spike transmission via the spikelet is dependent on the membrane potentials of both cells depolarizing together so that both cells are near threshold at the same time.

Our simulations using a generalized mathematical model of dendritic electrical coupling reveal further insights into the biophysical mechanisms of correlated activity. While the spikelets are responsible for the millisecond precision of correlated spiking, subthreshold membrane potential equalization due to coupling acts cooperatively with spike-to-spike transmission to ensure the robust degrees of correlated spiking as observed experimentally. Moreover, they show that precisely synchronous synaptic inputs to Golgi cells are only likely to dominate millisecond correlated spiking if they represent more than 80% of all inputs. This emphasizes the dominant role of electrical coupling in determining precisely correlated activity of Golgi cells. Our simulations furthermore reveal that these effects are largely independent of spike shape. The reduced model used in the simulation exhibits passive dendrites, as has been experimentally observed for Golgi cell dendrites (Vervaeke et al., 2012, but see Rudolph et al., 2015), like those of other types of interneurons (Hu et al., 2010). However, in electrically coupled cell types with active dendrites, spikelets may further summate with dendritic spikes to drive spiking even more effectively (Trenholm et al., 2014).

Recent experimental work in cerebellar slices (Dugué et al., 2009) and theoretical work (Chow and Kopell, 2000, Ostojic et al., 2009) have suggested that the low-pass filter represented by the gap junction conductance and the capacitance of the postjunctional cell should favor transmission of the spike afterhyperpolarization over the faster depolarizing spikelet itself. This in turn can lead to hyperpolarization and desynchronization of coupled neurons (Vervaeke et al., 2010). However, under our in vivo conditions, the spikelet has a predominantly excitatory effect, with its afterhyperpolarization appearing to be less functionally relevant than under in vitro conditions. While a hyperpolarization follows the peak of the spikelet in vivo (Figure 4B), the membrane potential does not drop below its value just prior to a spike. The importance of the depolarizing junction potential of the spikelet may be partly explained by voltage-dependent boosting of the spikelet amplitude (Curti and Pereda, 2004, Dugué et al., 2009, Mann-Metzer and Yarom, 1999) by the more depolarized membrane potentials observed in vivo. More importantly, however, both the experimentally observed and the simulated spikelet occur on a slow depolarizing ramp, which we argue is due to correlations in the ongoing synaptic input rate in vivo and the subthreshold membrane potential equalization between coupled neurons. Superimposed on this ramp, the hyperpolarizing junction potential due to the afterhyperpolarization following prejunctional spikes does not lead to a strong hyperpolarization below pre-spike baseline. Electrical coupling is usually studied in isolation of synaptic activity, while in vivo, under ketamine/xylazine anesthesia, we report high frequencies of spontaneous excitatory and inhibitory activity (73 ± 12 and 64 ± 11 Hz for EPSCs and IPSCs, respectively). In the awake animal, synaptic activities may be even higher, particularly during locomotion (e.g., see Powell et al., 2015), and thus spikelet afterhyperpolarizations may be even more strongly affected by enhanced degrees of synaptic excitation.

### Comparison with Other Brain Areas

The mechanism for millisecond precision correlated activity among electrically coupled interneurons we describe is simple and robust, and is likely to be widespread across the mammalian brain, given that electrically coupled interneurons can be found in many brain areas (Connors and Long, 2004, Galarreta and Hestrin, 2001) and have been shown to drive correlated activity in vitro (Galarreta and Hestrin, 1999, Gibson et al., 1999). Early single-unit recordings from unidentified interneurons in somatosensory cortex in vivo reported the occurrence of precisely correlated activity superimposed on a broader, slow correlated activity of tens of milliseconds (Swadlow et al., 1998), indicating that cortical interneurons can display both precisely and slow correlated activity. More recently, optogenetic identification of specific interneuron classes in combination with unit recordings in the prefrontal cortex (Kvitsiani et al., 2013) revealed that a subpopulation of pairs of parvalbumin-positive interneurons, but not somatostatin-positive units, could display millisecond precision correlated activity with each other and with some unidentified units. Dual patch-clamp recordings between pairs of interneurons in layer 2/3 of cerebral cortex revealed slow subthreshold membrane potential synchrony but correlated spike activity that was relatively imprecise (Gentet et al., 2010). This may be due to the fact that the recorded interneurons, which were not assigned to specific interneuron classes, may not have been electrically coupled, given that interneuron electrical coupling is specific to interneuron type (Connors and Long, 2004, Galarreta and Hestrin, 2001). Moreover, the slow subthreshold membrane potential synchrony was also observed between pairs of pyramidal cells and pairs of pyramidal cells and interneurons in the same preparation (Gentet et al., 2010, Lampl et al., 1999, Poulet and Petersen, 2008), suggesting that it arises from propagating waves of activity. More recent extracellular recordings of pairs of differing putative interneuron type in hippocampus revealed millisecond precision synchrony, arguing against a sole role of coupling and for a role of inhibition in mediating this synchrony (Diba et al., 2014). Thus, whether the mechanism we describe also drives correlated activity with millisecond precision among coupled cortical and hippocampal interneuron populations in vivo remains to be established.

Nevertheless, the importance of electrical coupling in cortical circuits has long been recognized at the level of network activity, where it contributes to and enhances oscillatory activity. For example, elimination of electrical coupling in Cx36-KO mice has been linked to slower theta oscillations in the hippocampus (Allen et al., 2011), as well as reduced power in gamma frequency and theta-phase modulation of gamma (Buhl et al., 2003). Interestingly, recent in vivo data showed that distinct interneuron subtypes mediate oscillations in theta and gamma frequency bands (Fukunaga et al., 2014). Together with these studies, our findings support the idea that electrical coupling in interneuron populations is important for orchestrating the temporal structure of network activity.

### Functional Role of Precisely Correlated Activity in the Cerebellar Circuit

The subthreshold membrane potential correlations combined with spike-to-spike transmission between Golgi cells ensure strong and precisely correlated activity in the face of spontaneous synaptic background activity. Furthermore, strong sensory-evoked bursts of synaptic inputs that drive spiking in individual cells enhance this precisely correlated activity. This correlated activity effectively serves to distribute MF and/or parallel fiber inputs throughout the coupled Golgi cell network, resulting in enhanced inhibition of downstream granule cells. It is known that tonic inhibition strongly determines the excitability and sensory responses of granule cells (Chadderton et al., 2004, Duguid et al., 2012). Temporally precise phasic inhibition, however, likely controls the spike timing of granule cells, and thus may control the occurrence of oscillatory or other synchronized patterns of granule cell activity (Dugué et al., 2009, Simões de Souza and De Schutter, 2011). As Golgi cells are coupled in local networks, the correlated activity of each unit in an ensemble would summate and strongly inhibit postsynaptic granule cells that were preferentially contacted by coupled Golgi cells. It will therefore be crucial to determine the exact spatial anatomical divergence and convergence of Golgi cell axons to granule cell dendrites and the location of the granule cells that are contacted by coupled ensembles of Golgi cells.

### Relevance of Golgi Cell-Correlated Activity to Motor Control

The ability to generate correlated inhibitory activity suggests that precisely timed feedforward inhibition (D’Angelo and De Zeeuw, 2009, Kanichay and Silver, 2008) and “time-windowing” of activity (D’Angelo, 2008, D’Angelo and De Zeeuw, 2009) play an important role in cerebellar computations. The exact relevance of precisely correlated activity between neighboring Golgi cells for cerebellar sensorimotor behavior remains to be determined, but some clues may be found in the phenotype of Cx36-KO mice, which have been shown to display impaired timing of locomotion, conditioned eye-blink responses, and motor learning (Frisch et al., 2005, Van Der Giessen et al., 2008, Zlomuzica et al., 2012). However, as Cx36 is also expressed in other cell types in the cerebellar circuit, including molecular layer interneurons, as well as in the inferior olive, which provides input to the cerebellum, a general Cx36-KO is not a good model to identify the behavioral relevance of Golgi cell-correlated activity. Nevertheless, specific ablation of Golgi cells in a transgenic mouse line expressing human interleukin-2 receptor α subunit using an immunotoxin-mediated cell targeting technique resulted in severe acute ataxia and a chronic inability to perform compound movements (Watanabe et al., 1998), highlighting the importance of this cell type in regulating motor control.

### Conclusion

Our data provide a mechanistic understanding of how electrical coupling causes correlated spikes in electrically coupled interneurons with millisecond precision in the context of the spontaneous and sensory-evoked synaptic drive in vivo. Importantly, the mechanism we describe should be relevant to most electrically coupled neurons in the brain, as it is generic and independent of spike shape. This suggests that electrically coupled interneurons will spike together with millisecond precision irrespective of the exact temporal structure of common synaptic input. Our results provide further support to the idea that electrical coupling is crucial for temporal computations performed in mammalian neural circuits.

## Experimental Procedures

Please see Supplemental Experimental Procedures for the full experimental procedures. Briefly, all animal procedures were performed under license from the UK Home Office in accordance with the Animal (Scientific Procedures) Act 1986. Male and female transgenic wild-type and connexin36 KO mice (P20-45) expressing EGFP under the GlyT2 promotor were used to identify and target Golgi cells. In vivo targeted patch-clamp recordings (Margrie et al., 2003) were performed using a custom two-photon microscope (MOM, Sutter) to visualize EGFP positive Golgi cells in Crus II under ketamine/xylazine anesthesia. Sensory stimulation was performed used an airpuff (100 ms, 30–40 psi) delivered to the perioral region and/or whisker pad.

Simulations were performed using NEURON 7.1 (Hines and Carnevale, 1997). Two reduced compartmental neuron models of Golgi cells, each consisting of a soma and a dendrite, were coupled via a gap junction (resistance, 3 GΩ) at a proximal dendritic location (Vervaeke et al., 2010). The gap junction was operated in different modes (Figure 6) in which it was switched on or off selectively during subthreshold signaling, the DJP, and/or the HJP. Ongoing synaptic inputs in vivo were simulated by Poisson spike trains triggering synaptic conductances that were distributed uniformly over the soma and dendrites of both neurons.

## Author Contributions

I.v.W. performed all experiments and analyses with the exception of the single whole-cell recordings of sensory-evoked responses and their analyses, which were performed by S.S.H. and S.K. The simulations were performed by A.R. The study was designed by I.v.W. and M.H., who also wrote the paper.

## Supplemental Information

Document S1. Figures S1–S7 and Supplemental Experimental Procedures

Document S2. Article plus Supplemental Information
