# Supplementary material for: Conditional Spike Transmission Mediated by Electrical Coupling Ensures Millisecond Precision-Correlated Activity among Interneurons In Vivo
Source: Neuron. 2016 May 18;90(4):810–23. doi: 10.1016/j.neuron.2016.04.013 (PMC4882376; doi:10.1016/j.neuron.2016.04.013)
Supplement: Document S1. Figures S1–S7 and Supplemental Experimental Procedures [file mmc1.pdf]

Neuron, Volume *90*

Supplemental Information

Conditional Spike Transmission Mediated by Electrical Coupling Ensures Millisecond Precision-Correlated Activity among Interneurons In Vivo

Ingrid van Welie, Arnd Roth, Sara S.N. Ho, Shoji Komai, and Michael Häusser

Supplemental Figures

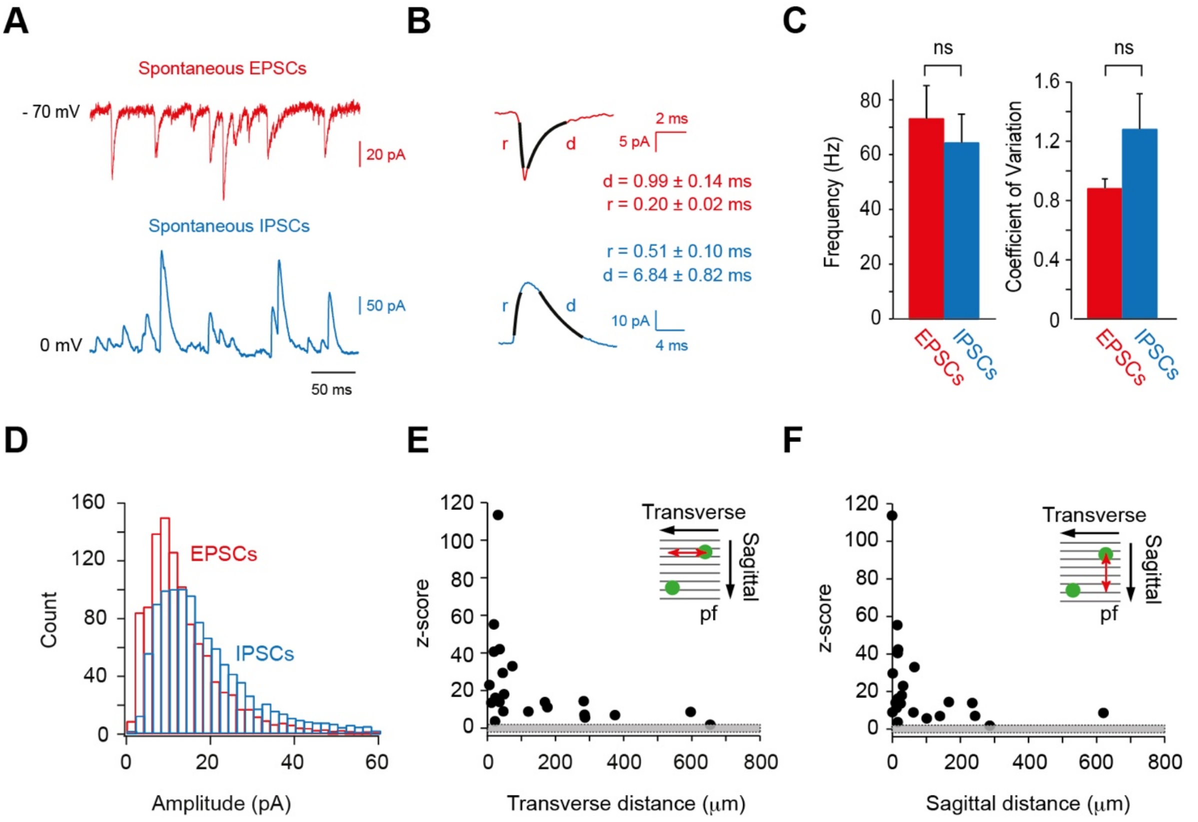

Figure S1 (related to Figure 1). Background synaptic activity in Golgi cells and distance-dependence of coupling in transverse and sagittal planes

(A) Using a cesium-based internal, spontaneous EPSCs were recorded at -70 mV and spontaneous IPSCs were recorded at 0 mV. (B) Mean EPSC and IPSC waveforms from the example recordings shown in (A). Mean waveforms were fitted with single exponential functions to obtain kinetic values for EPSC rise and decay times. The mean values across cells for rise (r) and decay (d) times are indicated next to the EPSC and IPSC waveforms (n = 6 neurons). (C) Summary bar graph of mean ± s.e.m. of spontaneous EPSC and IPSC frequencies and the coefficient of variation of event times for both (n = 6 neurons). (D) Mean amplitude distribution of all identified EPSCs and IPSCs across cells (n = 6 neurons). (E) Degree of correlated activity found in Golgi cell pairs at various inter-neuronal distances along the transverse axis (inset displays field of view from the top of the brain and how transverse distance was measured between cell somata; pf = parallel fibers). (F) Degree of correlated activity found in Golgi cell pairs at various inter-neuronal distances along the sagittal axis. Grey areas in (E) and (F) indicate confidence interval (z-scores between -2 to +2).

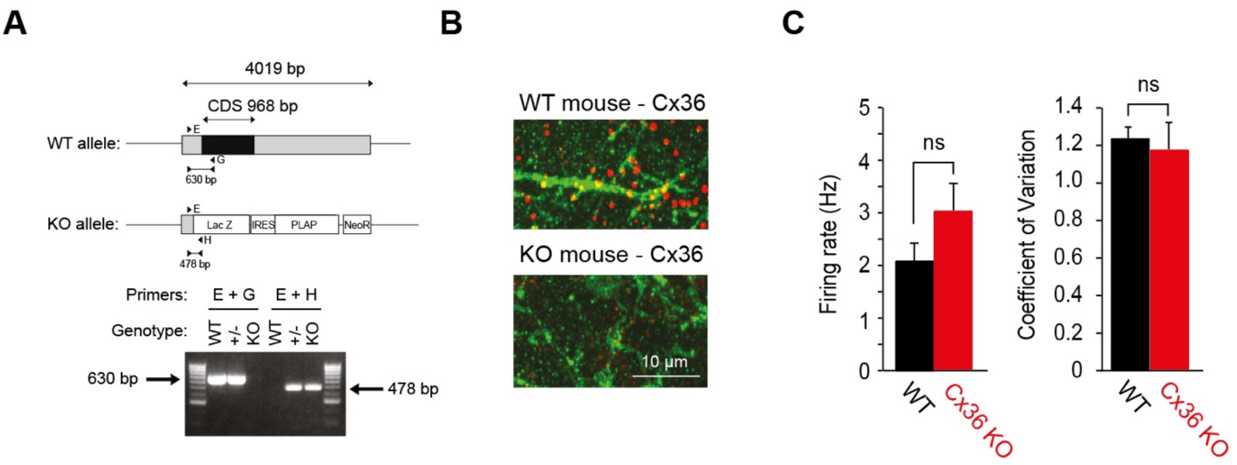

Figure S2 (related to Figure 2). Characterization of GlyT2-EGFP/Cx36 KO mice

(A) A new mouse line was created by crossing the GlyT2-EGFP mice with Cx36-KO mice. To confirm that the wildtype (WT) Cx36 gene sequence was still disrupted in the new mouse line, we used a unique set of primers (E, G & H) to identify both the WT and KO allele (see Supplemental Experimental Procedures). On the bottom an example PCR result summarizing results for the identification of WT, heterozygous and homozygous KO mice. Only homozygous mice were used for experiments. (B) To confirm the genotype of mice, we performed immunolabelling for the Cx36 protein in WT and KO mice. In WT mice, numerous Cx36 puncta are present in the molecular layer of the cerebellum many of which co-localize with Golgi cell dendrites, while these are largely absent in the KO mice. (C) Summary bar graph comparing the mean firing rates and the coefficient of variation of interspike intervals of Golgi cells in WT and Cx36-KO mice. Neither parameter was significantly different ($p = 0.06$ and $p = 0.13$ respectively).

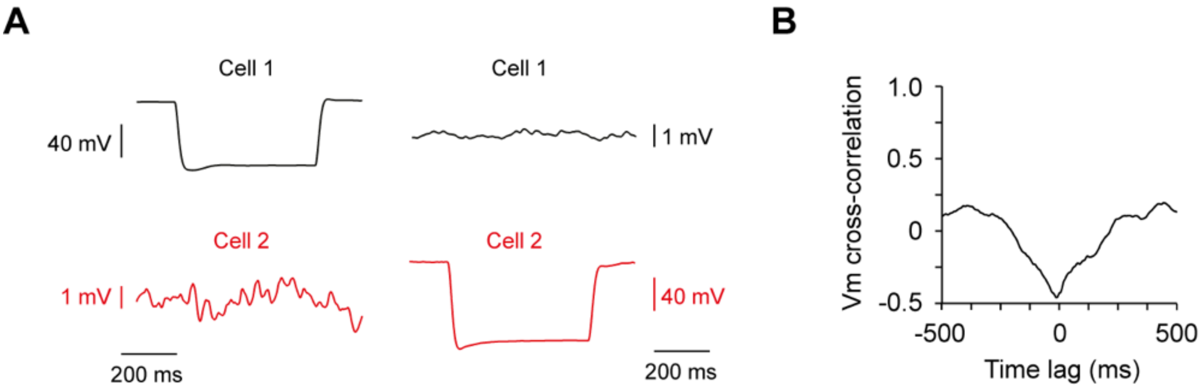

Figure S3 (related to Figure 3). Dual whole-cell recording from a non-coupled Golgi cell pair

(A) Simultaneous dual whole-cell recording from two Golgi cells that were located nearby (intersomatic distance = 24 μm), but were not electrically coupled: injecting hyperpolarizing current into one cell produced no change in membrane potential of the other cell. (B) There was no positive, but instead a negative cross-correlation of membrane potentials in these two non-coupled Golgi cells.

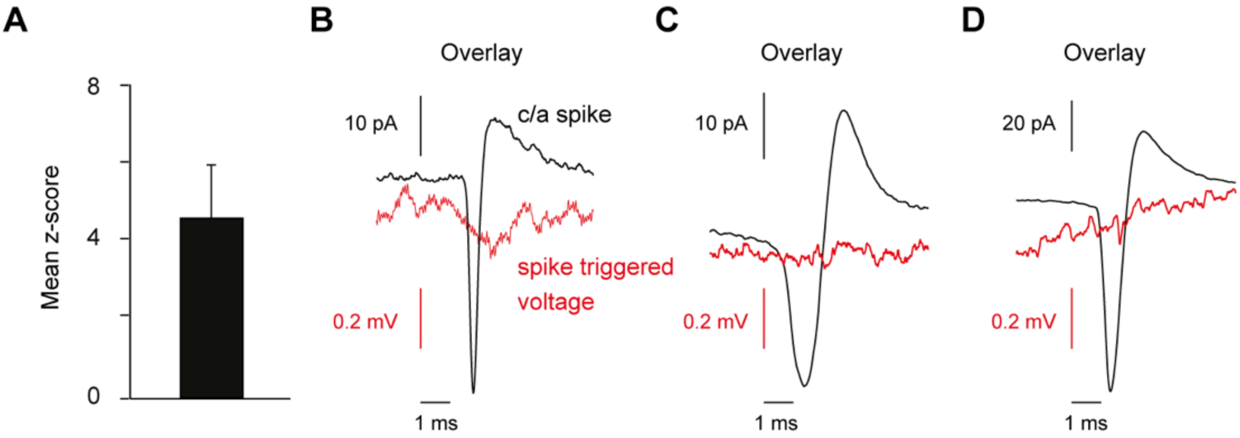

Figure S4 (related to Figure 4). In pairs with low precisely correlated activity, spikelets are absent

(A) Mean z-score (5 ± 1, n = 3) from pairs in which no to low precisely correlated activity was found. (B-C) Overlay of the mean cell-attached (c/a) spike (red trace) and the corresponding spike-triggered voltage (black trace) in the w/c recording in the 3 pairs in which there was no to low degrees of precisely correlated spiking (see also Figure S5). In all three examples, there were no spike-triggered depolarizing membrane potential changes beyond noise fluctuations (mean depolarization = 0.05 ± 0.01 mV in first 1.5 ms following c/a spike, n = 3), suggesting that these pairs were not electrically coupled.

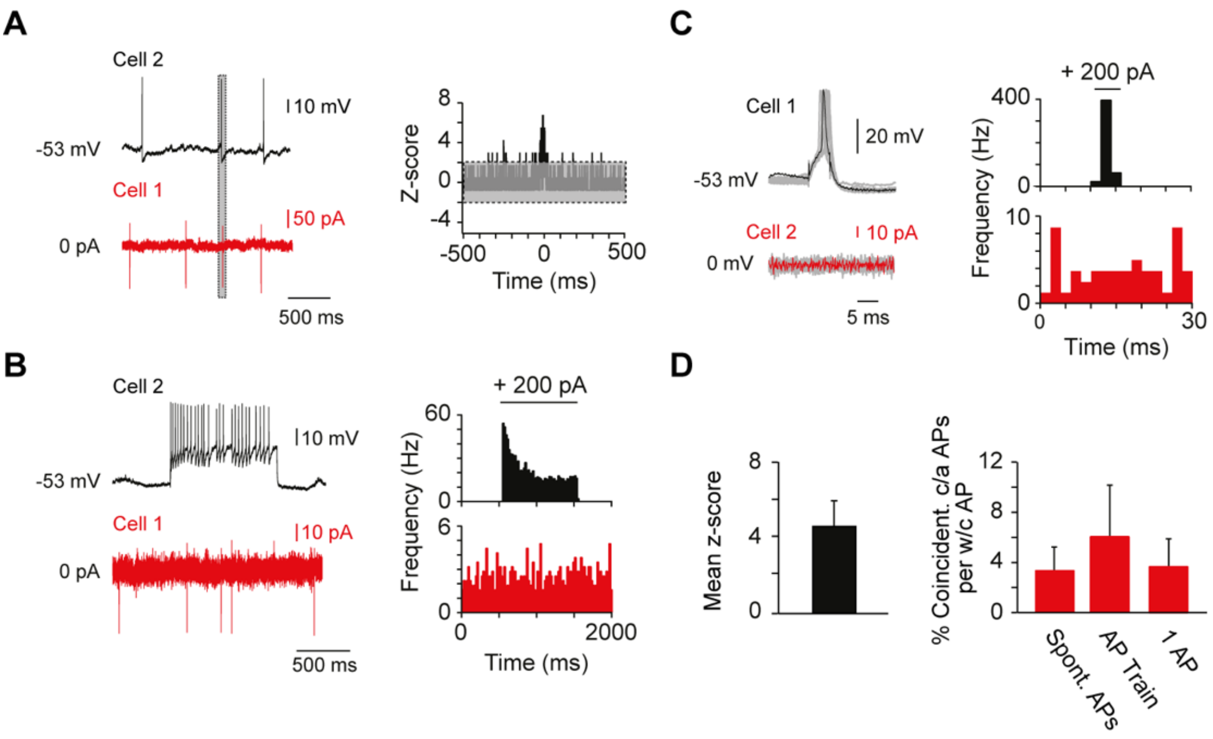

Figure S5 (related to Figure 5). In pairs with low precisely correlated activity, induced spikes in one cell do not trigger spikes in the paired cell

(A) Example traces (left) and spike time cross-correlogram (right) of a dual whole-cell (w/c) and cell-attached (c/a) recording (same experimental configuration as in Figures 4 and 5), in which the spontaneous spiking of the pair was only mildly correlated (max z-score = 7, cross-correlogram left). Grey area on trace indicates the occurrence of correlated spikes (< ± 5 ms time lag). Grey area on cross-correlogram indicates confidence interval (z-scores between -2 to +2). (B) In the same pair as in (A), inducing a train of action potentials in Cell 2 failed to induce correlated action potentials in Cell 1 as is evident from the PSTH (right, 20 ms bins) summarizing the results across multiple trials. (C) Similarly, inducing single action potentials in Cell 2 failed to induce any correlated spikes in Cell 1 across trials (PSTH 2 ms bins). (D) Summary data across 3 such non- to mildly correlated pairs in which spike injection experiments failed to induce correlated spikes. The mean z-score across these pairs was 5 ± 1 (left) and the mean percentages of correlated spikes associated with each w/c spike were very low across the different conditions (3 ± 2%, 6 ± 4% and 4 ± 2%, respectively).

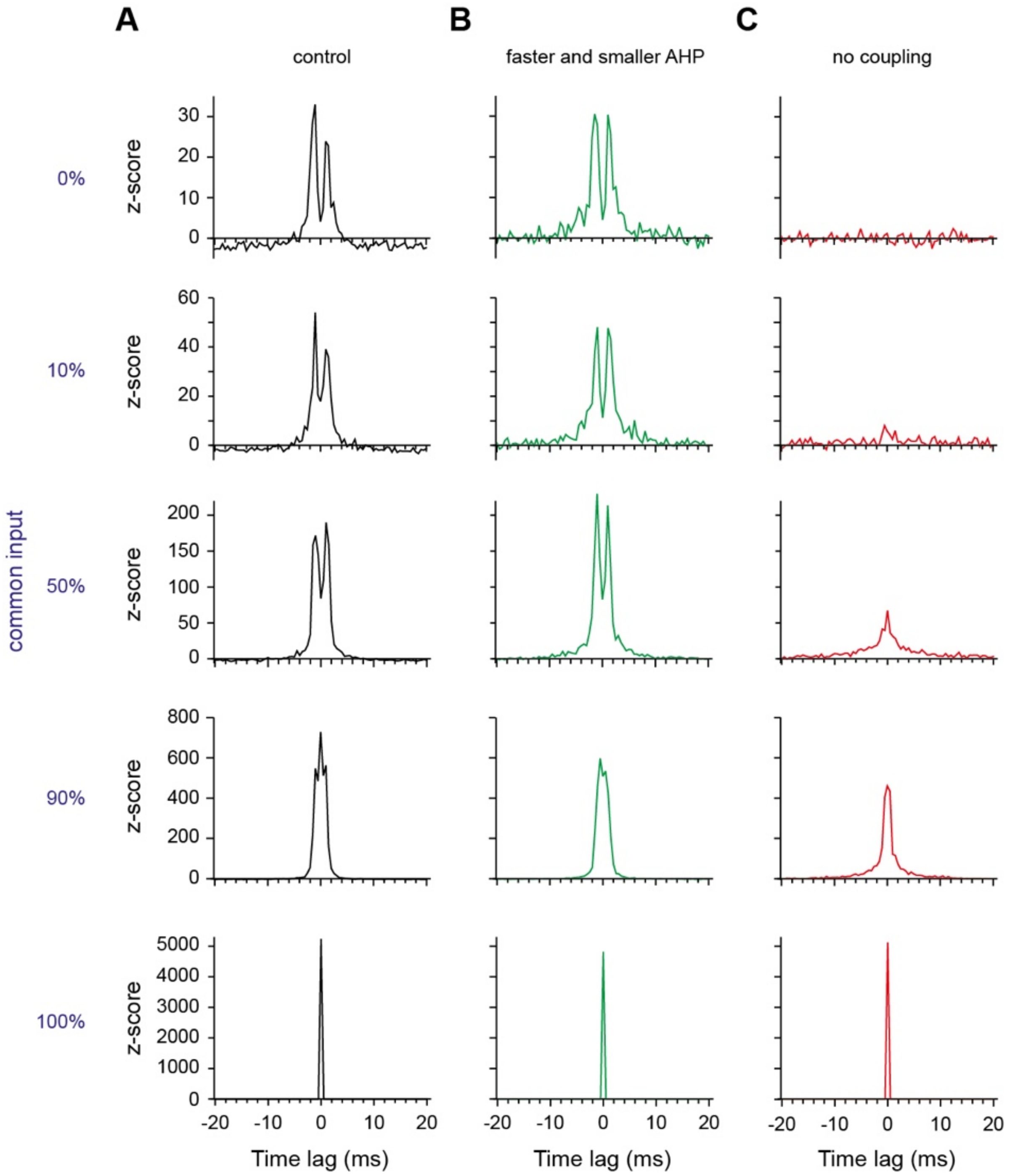

Figure S6 (related to Figure 6). Effect of degree of precisely synchronous common input and spike shape on degree of correlated activity

(A) Cross-correlograms of spike timing of two coupled Golgi cells in a computational model as a function of increasing degrees of precisely synchronous common input. Under control conditions, the somatic spike shape has a long and deep AHP (amplitude, 17 mV; duration, 14 ms FWHM). (B) Same data as in a, but using a somatic spike with a faster and smaller AHP (amplitude, 8 mV; duration, 9 ms FWHM). (C) Cross-correlograms as a function of increasing degrees of precisely synchronous common input in the absence of electrical coupling.

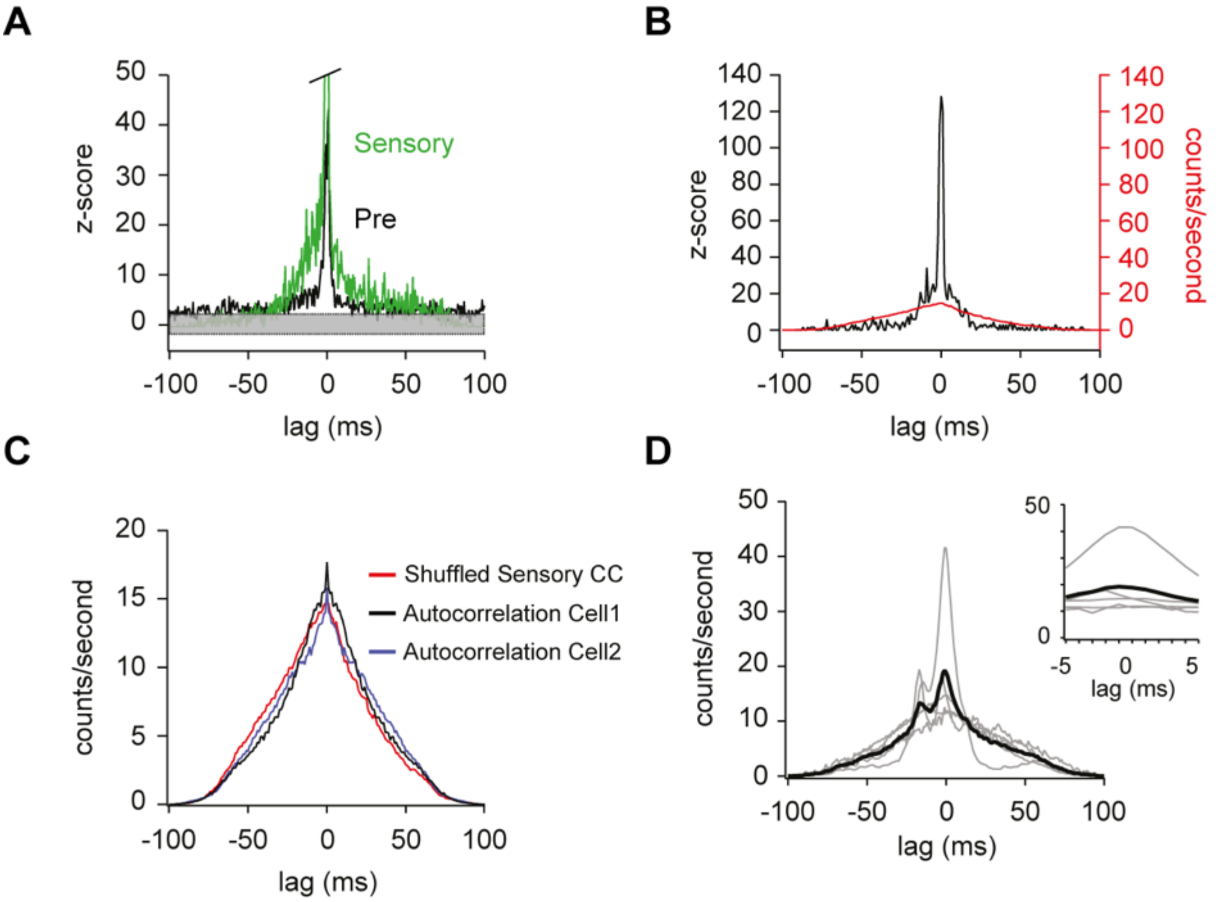

Figure S7 (related to Figure 8). Variation in temporal profile of sensory-evoked responses in individual cells and pairs

(A) Mean cross-correlograms (n = 5) computed from baseline spontaneous spiking (Pre, black trace) and during sensory stimuli (Sensory, green trace). Correlations at slower time lags are enhanced during sensory stimuli. The peak of the sensory evoked correlation is clipped for display purposes. Grey area indicates confidence interval (z-scores between -2 to +2). (B) Cross-correlogram (black trace) of sensory-evoked spiking from the same pair of Golgi cells as shown in Figure 8B and 8C. Shuffling the spike times of the sensory evoked spikes across trials in one cell of the pair reveals a slow temporal correlation (red trace) that resembles the base or "foot" of the real cross-correlogram. (C) The shuffled sensory-evoked cross-correlogram (red trace) closely resembles the autocorrelations of the spike times in each cell of the pair (black and blue traces), suggesting that the variation in spike timing in individual Golgi cells leads to the broad temporal correlation reflected in the base of the cross-correlogram in sensory evoked spiking in a Golgi cell pair as shown in (A). (D) Individual (grey traces) and mean (black trace) shuffled sensory-evoked cross-correlograms indicating broad temporal correlations in sensory evoked spiking in all pairs (n = 5). Inset shows same figure as (D), but only showing time lags from -5 to 5 ms, to directly compare with Figure 8G. Compared to Figure 8G, there is no sharp peak around 0 ms.

Supplemental Experimental Procedures

Transgenic animals and surgical preparation

Animal procedures were performed under license from the UK Home Office in accordance with the Animal (Scientific Procedures) Act 1986. Male and female transgenic mice expressing EGFP under the GlyT2 promotor (Zeilhofer et al., 2005) were backcrossed into C57Bl6 mice for multiple generations (F5-F19) and were used to identify and target Golgi cells (Simat et al., 2007). Mice (P20-45) were anaesthetized by intraperitoneal injection of ketamine (80-100 mg kg^{-1}) / xylazine (5-10 mg kg^{-1}). Body temperature was monitored using a rectal probe and maintained at 37°C using a homeothermic blanket. Craniotomies (1 mm^2) were made over Crus II. The dura was removed and the brain was covered using 1.5% agarose in HEPES buffered artificial cerebral spinal fluid (150 mM NaCl, 2.5 mM KCl, 10 mM HEPES, 2 mM $CaCl_2$ and 1 mM $MgCl_2$) to reduce breathing related movement. Connexin36 knockout (Cx36-KO) mice (Deans et al., 2001) were crossed with heterozygous GlyT2-EGFP mice and bred further to generate mice homozygous for the Cx36 gene and heterozygous for EGFP driven under the GlyT2 promoter. To confirm that the wildtype (WT) Cx36 gene sequence had been disrupted, we used a new set of primers to amplify through the start codon region of the Cx36 gene. We designed a common forward PCR primer, E (5' CTC AGA CCG CAA GAT CGC G 3') to the upstream non-coding region of the Cx36 gene (Gene ID 320846), and a 5' primer, G (5' CAC TGG GCC TCG GGT AAT 3') in the WT downstream coding sequence. The common forward primer was used in combination with either primer G, or a Lac Z reverse primer, H (5' GCC TCT TCG CTA ATT ACG 3'), corresponding to the bicistronic cassette which was used to disrupt Cx36, to amplify the target alleles. Routine genotyping of Cx36-KO/GlyT2-EGFP mice was performed using primers targeting the neo cassette in the transgenic genome.

In vivo single and dual patch-clamp recordings

In vivo targeted patch-clamp recordings (Margrie et al., 2003) were performed using a custom two-photon microscope (MOM, Sutter) to visualize EGFP positive cells in the cerebellar granule cell layer. The pipette solution used for cell-attached and whole-cell recordings contained (in mM): 125 K-Methanesulphonate, 7 KCl, 10 HEPES, 2 Mg-ATP, 2 Na_2ATP, 0.5 Na_2GTP, 0.05 EGTA and 50 µM Alexa Fluor 594 (pH 7.3 with KOH, 280-290 mOsm). For recordings of spontaneous EPSCs and IPSCs within single cells, a Cs-based internal was used containing (in mM): 120 Cs-Methanesulphonate, 10 HEPES, 2 Mg-ATP, 2 Na_2-ATP, 0.5 Na_2GTP, 5 EGTA, 5 QX-314, 10 TEA-Cl and 50 µM Alexa Fluor 594 (pH 7.3 with CsOH, 280-290 mOsm). Recordings were made using a Multiclamp 700B amplifier (Molecular Devices) and a Digidata acquisition system (Molecular Devices). Data were filtered at 3-10 kHz and acquired at 20 or 40 kHz using pClamp 9.0 software (Molecular Devices). Patch pipettes were pulled using a vertical puller (Narishige) from thick walled borosilicate glass and had a resistance of 5-7 MΩ. Access resistance for whole-cell recordings was between 20-70 MΩ. Membrane potentials were not corrected for liquid junction

potentials. For the purpose of analysis of baseline correlated activity and for spike induction protocols, only pairs in which both cells were spiking and in which extracellular spikes could be easily distinguished from noise by eye were included for analysis. Sensory stimulation was performed by delivering a 100 ms airpuff (30-40 psi) to the perioral region and/or whisker pad using a pneumatic pressure device (PDES-02DX, NPI electronic GmbH). There was an experimentally determined 15 ms delay before the pressurized air reached the mouse. Only pairs with a latency in the sensory response < 50 ms following this 15 ms delay were used for analysis.

Immunocytochemistry

For immunodetection of Cx-36, WT GlyT2-EGFP and Cx36-KO/GlyT2-EGFP mice were perfused with 4% paraformaldehyde (PF). The protocol used for immunodetection of Cx36 was as previously published (Vervaeke et al., 2012). In this protocol, tissue was not post-fixed, and this led to loss of EGFP signal in our tissue. As a consequence, an antibody against mGluR2 (which labels Golgi cells specifically) was used to label Golgi cell dendrites. In short, for co-detection of both Cx36 and mGluR2, slices (60 µm) were cut and washed in PBS, and then incubated in primary antibody (mouse monoclonal anti-Cx36, MAB3045, Chemicon and rabbit polyclonal anti-mGluR2/3, 06-676, Millipore, Billerica, MA) overnight at room temperature. After washing in PBS, slices were incubated in secondary antibody for 2 hours at room temperature. Slices were then washed again with PBS and mounted using Vectashield mounting medium. A confocal microscope system (Perkin-Elmer spinning disk) was used to take images of labeled tissue.

Event detection and analysis

Spike and synaptic event detection was performed off-line using custom written software in Igor Pro (Tarotools, courtesy of Taro Ishikawa). Events were detected using a threshold and subsequently individually verified manually. As a complementary method, a deconvolution-based algorithm (Pernia-Andrade et al., 2012) implemented in Stimfit (courtesy of Christoph Schmidt-Hieber) was used to detect synaptic events based on an average kinetic template of individual synaptic events. After low-pass filtering of the deconvolution result at 0.2 and using a detection threshold (θ) of 4 (4 times the standard deviation of the baseline Gaussian noise), this method gave results similar to that of manual verification. Cross-correlograms of spike times or synaptic event times were computed using custom-written code in Matlab. Cross-correlograms were normalized by creating 30 shuffled spike trains for each recording and subtracting the mean shuffled cross-correlogram from the raw mean cross-correlogram and by dividing by the standard deviation of the mean shuffled cross-correlogram. Z-scores between ± 2 were taken as the confidence interval, outside of which values were considered significant. Z-scores were computed from cross-correlograms using 0.5 ms bins. Spike coincidence percentages were standardly computed from cross-correlograms using 1 ms bins unless noted otherwise. The EPSC rate cross-correlation was computed by binning synaptic event

times, subtracting the mean from the resulting input rate functions, and computing the normalized cross-correlation of the mean-subtracted input rates so that the autocorrelations at zero lag equal 1. For cross-correlograms of membrane voltage or current, Matlab's built-in xcorr function was used and spectral analysis was performed using Matlab's built-in mscohere function. Spike-triggered averages of spikelet voltages and currents were determined in Igor Pro using Neuromatic (courtesy of Jason Rothman). Statistics were performed using Instat 3 (Graphpad). Two-tailed non-parametric Mann-Whitney tests were used for comparing unpaired data and two-tailed repeated measures Anova using standard parametric measures & Tukey post comparison testing or a two tailed Student's T-test were used for comparing paired data. Data is reported as mean ± standard error of the mean (s.e.m.).

Model

Simulations were performed using NEURON 7.1 (Hines and Carnevale, 1997). Two reduced compartmental neuron models, each consisting of a soma and a dendrite, were coupled via a gap junction (resistance, 3 GΩ) at a proximal dendritic location corresponding to 25% of the length of each dendrite (Vervaeke et al., 2010). Both the soma (length = 30 μm; diameter = 30 μm) and dendrite (length = 200 μm, subdivided into 50 compartments; diameter = 1 μm) of each neuron had homogeneous passive membrane properties (specific membrane capacitance C_m = 1 μF/cm^2; specific membrane resistance R_m = 20000 Ω cm^2). The passive reversal potential was set to E = –46 mV to account for the steady-state depolarization of Golgi cells due to summation of long-lasting kainate receptor-mediated components of synaptic currents (Bureau et al., 2000) during ongoing activity *in vivo*. The intracellular resistivity was R_i = 150 Ω cm. The soma of each model neuron contained a generic action potential generation mechanism. When the somatic voltage threshold of –44 mV was reached in one of the neurons, a fixed action potential current waveform described by a difference between two probability density functions of skew-normal distributions and a decaying exponential was injected into the soma of that neuron. These current waveforms were pre-computed in Mathematica. The standard action potential current waveform was given by:

```
Table[2.5 PDF[SkewNormalDistribution[0.2, 0.2, 2], t] - 2.9
PDF[SkewNormalDistribution[0.7, 0.5, 2], t] - 0.01 Exp[-t/30.], {t, 0., 65.9, 0.1}]
```

and the action potential waveform with faster and smaller AHP was given by:

```
Table[2.5 PDF[SkewNormalDistribution[0.2, 0.2, 2], t] - 2.6
PDF[SkewNormalDistribution[0.7, 0.5, 2], t] - 0.01 Exp[-t/3.], {t, 0., 65.9, 0.1}]
```

where time t is given in ms, and current in nA.

The voltage waveform of the action potential was governed by the response of the compartmental model to the injected action potential current waveform. Accordingly, the voltage waveform was dependent on dendritic location, and was transmitted across the gap junction from the pre-junctional to the post-junctional neuron model,

arriving at the post-junctional soma as a DJP and HJP waveform. To separately test the influence of the DJP, HJP and the coupling of subthreshold membrane potential via the gap junction on the synchrony of spiking of the two neurons, the gap junction was operated in different modes (Fig. 6) in which it was switched on or off selectively during subthreshold signaling, the DJP and/or the HJP. The duration of the DJP at the pre-junctional end of the dendritic gap junction (1.65 ms) was used for gating the gap junction during the DJP, while gating for the HJP was performed for 180 ms, beginning at the end of the DJP at the pre-junctional end of the gap junction. The value of 180 ms was chosen such that the HJP had decayed to less than 0.1% of its peak amplitude at the end of the gating period.

Ongoing synaptic inputs *in vivo* were simulated by independent Poisson spike trains triggering synaptic conductances, which were distributed homogeneously per membrane area over the soma and dendrites of both neurons. In the case of 0% precisely synchronous common input, each synapse was driven by its own, independent Poisson spike generator, whereas in the case of x% precisely synchronous common input, x% of excitatory synapses and x% of inhibitory synapses on the pre-junctional neuron each received the same Poisson input spike trains as their counterparts on the post-junctional neuron. The time course of each synaptic conductance was described by a sum of two exponentials. Excitatory synaptic conductances had a peak amplitude of 0.25 nS, a rise time constant of 0.2 ms and a decay time constant of 1.0 ms. The excitatory reversal potential was 0 mV, and excitatory synapses were activated at a total rate (summed over all synapses on one neuron) of 240 Hz. Inhibitory synaptic conductances also had a peak amplitude of 0.25 nS, but a rise time constant of 0.5 ms and a decay time constant of 5 ms. The inhibitory reversal potential was –75 mV, and inhibitory synapses were activated at a total rate of 120 Hz per neuron. The time step of the simulation was 0.025 ms, and the simulated time for each cross-correlogram was 10,000 s. Cross-correlograms of simulated spike trains were calculated in the same way as cross-correlograms of experimentally measured spike trains.

Supplemental References

Bureau, I., Dieudonne, S., Coussen, F. & Mulle, C. Kainate receptor-mediated synaptic currents in cerebellar Golgi cells are not shaped by diffusion of glutamate. *Proc Natl Acad Sci USA* **97**, 6838-6843 (2000).

Deans, M.R., Gibson, J.R., Sellitto, C., Connors, B.W. & Paul, D.L. Synchronous activity of inhibitory networks in neocortex requires electrical synapses containing connexin36. *Neuron* **31**, 477-485 (2001).

Hines, M.L. & Carnevale, N.T. The NEURON simulation environment. *Neural Comput* **9**, 1179-1209 (1997).

Margrie, T.W.*, et al.* Targeted whole-cell recordings in the mammalian brain in vivo. *Neuron* **39**, 911-918 (2003).

Pernia-Andrade, A.J.*, et al.* A deconvolution-based method with high sensitivity and temporal resolution for detection of spontaneous synaptic currents in vitro and in vivo. *Biophys J* **103**, 1429-1439 (2012).

Simat, M., Ambrosetti, L., Lardi-Studler, B. & Fritschy, J.M. GABAergic synaptogenesis marks the onset of differentiation of basket and stellate cells in mouse cerebellum. *Eur J Neurosci* **26**, 2239-2256 (2007).

Vervaeke, K.*, et al.* Rapid desynchronization of an electrically coupled interneuron network with sparse excitatory synaptic input. *Neuron* **67**, 435-451 (2010).

Vervaeke, K., Lőrincz, A., Nusser, Z. & Silver, R.A. Gap junctions compensate for sublinear dendritic integration in an inhibitory network. *Science* **335**, 1624-1628 (2012).

Zeilhofer, H.U.*, et al.* Glycinergic neurons expressing enhanced green fluorescent protein in bacterial artificial chromosome transgenic mice. *J Comp Neurol* **482**, 123-141 (2005).
