# Supplementary material for: Conditional Spike Transmission Mediated by Electrical Coupling Ensures Millisecond Precision-Correlated Activity among Interneurons In Vivo
Source: Neuron. 2016 May 18;90(4):810–23. doi: 10.1016/j.neuron.2016.04.013 (PMC4882376; doi:10.1016/j.neuron.2016.04.013)
Supplement: Document S2. Article plus Supplemental Information [file mmc2.pdf]

Neuron

Article

Conditional Spike Transmission Mediated by Electrical Coupling Ensures Millisecond Precision-Correlated Activity among Interneurons In Vivo

Highlights

- Double patch-clamp recordings from Golgi cells reveal millisecond synchrony in vivo
- Millisecond synchrony requires gap junctions and is enhanced by sensory stimuli
- Gap junctions drive synchrony via slow V_m equalization and fast spikelet transmission
- Modeling shows these findings can be generalized to any electrically coupled neurons

Authors

Ingrid van Welie, Arnd Roth, Sara S.N. Ho, Shoji Komai, Michael Häusser

Correspondence

m.hausser@ucl.ac.uk

In Brief

van Welie et al. show using double patch-clamp recordings that cerebellar Golgi cells display millisecond precise correlated activity in vivo, which is enhanced during sensory processing. Gap junctions mediate precise correlated activity via slow membrane potential equalization and fast spikelet transmission.

CrossMark

van Welie et al., 2016, Neuron *90*, 810–823
May 18, 2016 © 2016 The Authors. Published by Elsevier Inc.
http://dx.doi.org/10.1016/j.neuron.2016.04.013

CellPress

Neuron
Article

Conditional Spike Transmission Mediated by Electrical Coupling Ensures Millisecond Precision-Correlated Activity among Interneurons In Vivo

Ingrid van Welie,[1] Arnd Roth,[1] Sara S.N. Ho,[1] Shoji Komai,[2] and Michael Häusser[1,*]
[1]Wolfson Institute for Biomedical Research and Department of Neuroscience, Physiology, and Pharmacology, University College London, Gower Street, London WC1E 6BT, UK
[2]Nara Institute of Science and Technology, 8916-5 Takayama, Ikoma, Nara 630-0192, Japan
*Correspondence: m.hausser@ucl.ac.uk
http://dx.doi.org/10.1016/j.neuron.2016.04.013

SUMMARY

Many GABAergic interneurons are electrically coupled and in vitro can display correlated activity with millisecond precision. However, the mechanisms underlying correlated activity between interneurons in vivo are unknown. Using dual patch-clamp recordings in vivo, we reveal that in the presence of spontaneous background synaptic activity, electrically coupled cerebellar Golgi cells exhibit robust millisecond precision-correlated activity which is enhanced by sensory stimulation. This precisely correlated activity results from the cooperative action of two mechanisms. First, electrical coupling ensures slow subthreshold membrane potential correlations by equalizing membrane potential fluctuations, such that coupled neurons tend to approach action potential threshold together. Second, fast spike-triggered spikelets transmitted through gap junctions conditionally trigger postjunctional spikes, depending on both neurons being close to threshold. Electrical coupling therefore controls the temporal precision and degree of both spontaneous and sensory-evoked correlated activity between interneurons, by the cooperative effects of shared synaptic depolarization and spikelet transmission.

INTRODUCTION

The cerebellum is thought to play a central role in governing the timing of movements. To understand how the cerebellum mediates temporally precise motor control, we need to understand the mechanisms underlying temporal coding in the cerebellar circuitry (Heck et al., 2013; Person and Raman, 2012b). Various types of correlated activity have been reported at the output stage of the cerebellar cortex, in the form of Purkinje cell complex spike synchrony (Bell and Kawasaki, 1972; Sasaki et al., 1989) and Purkinje cell simple spike synchrony (Heck et al., 2007). It has also been suggested that Purkinje cell simple spike synchrony will lead to time-locked activity of downstream target neurons in the deep cerebellar nuclei (Person and Raman, 2012a). However, to understand temporal coding regimes at the output stage of the cerebellar cortex, we must first determine how temporal codes are formed and transmitted at earlier stages in the circuit, starting with the temporal integration of mossy fiber inputs in the sensory input layer.

The granule cell layer forms the input layer of the cerebellar cortex and receives mossy fiber inputs conveying sensory and motor information that are integrated by two cell types: the excitatory granule cells and inhibitory Golgi cells. Golgi cells locally inhibit the excitatory granule cells and each other (Hull and Regehr, 2012). They have large receptive fields (Holtzman et al., 2006b; Tahon et al., 2005; Vos et al., 1999b) and have been reported to display a combination of responses to mossy fiber inputs: short-latency excitation alone, long-latency inhibition alone, or a combination of both (Holtzman et al., 2006a, 2006b; Prsa et al., 2009; Tahon et al., 2005, 2011; Volny-Luraghi et al., 2002; Vos et al., 1999b). Golgi cell inhibition is traditionally thought to control the gain of granule cell excitation, thus ensuring sparse granule cell spiking (Marr, 1969), a hypothesis supported by the finding that, both in vitro and in vivo, tonic inhibition controls the gain of granule cell excitability (Chadderton et al., 2004; Duguid et al., 2012; Mitchell and Silver, 2003). However, Golgi-to-granule-cell connectivity patterns, which display a strong divergence of single Golgi cell axons onto many granule cells and the convergence of several Golgi cells onto individual granule cells, have prompted the hypothesis that Golgi cells also control the spatiotemporal patterning of granule cell activity (D'Angelo, 2008). This in turn can drive loose correlated activity of Golgi cells via the parallel fibers (Vos et al., 1999a). As such, it has been hypothesized that feedforward Golgi cell inhibition may provide a time-windowing function by limiting granule cell responsiveness to mossy fiber inputs (D'Angelo, 2008; D'Angelo and De Zeeuw, 2009).

In common with many other interneuron types (Connors and Long, 2004; Galarreta and Hestrin, 2001) in the mammalian brain, Golgi cells are electrically coupled (Dugué et al., 2009; Vervaeke et al., 2010). The contribution of electrical coupling to precisely correlated activity between interneurons, while often assumed to be important, is controversial, with experimental and theoretical work suggesting that both synchronization (Dugué et al., 2009; Galarreta and Hestrin, 1999; Gibson et al., 1999; Landisman et al., 2002; Long et al., 2005; Mann-Metzer and Yarom, 1999) and desynchronization (Chow and Kopell,

 © 2016 The Authors. Published by Elsevier Inc.
This is an open access article under the CC BY license (http://creativecommons.org/licenses/by/4.0/).

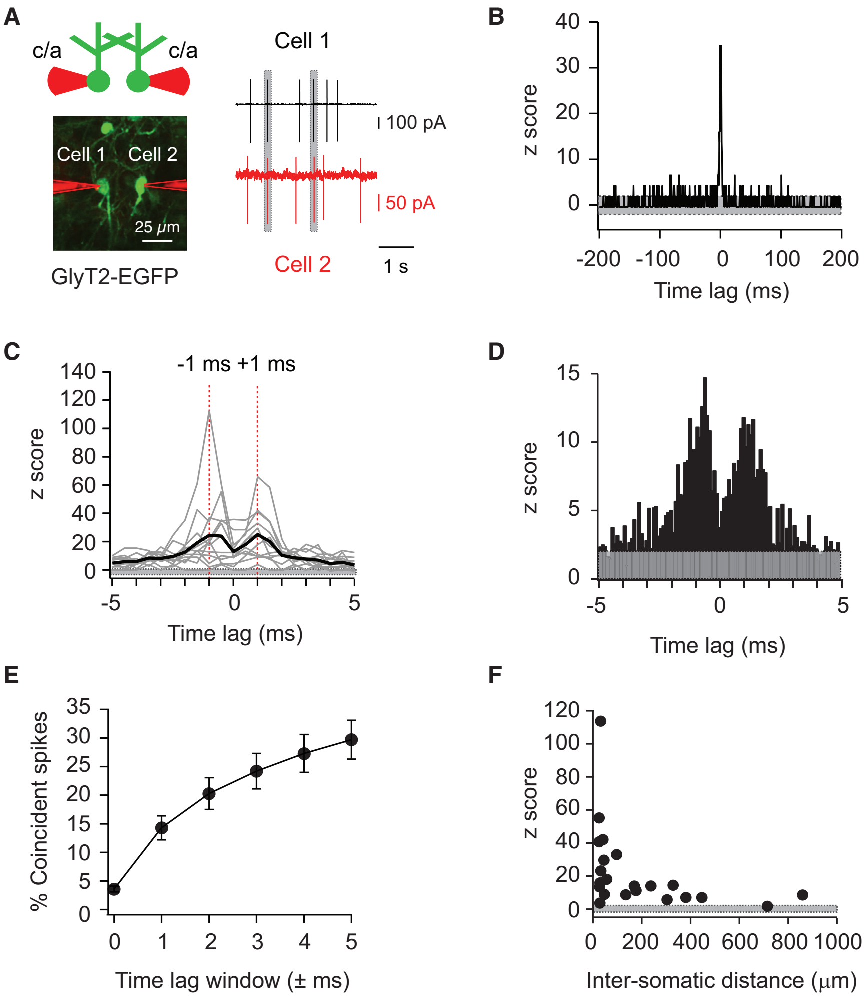

Figure 1. Golgi Cell Pairs Display Millisecond Precision-Correlated Activity

(A) Recording configuration for measuring spike time correlations between two Golgi cells in vivo. Two EGFP-expressing Golgi cells (left) were targeted using two-photon guidance, and spike trains were recorded using dual loose patch cell-attached (c/a) recordings (right). Gray areas on spike trains indicate the occurrence of correlated spikes (< ±5 ms time lag).

(B) The cross-correlogram (0.5 ms bins) computed from the example spike trains in (A) shows a large peak around 0 ms.

(C) The cross-correlograms (0.5 ms bins) for all pairs recorded within 100 μm of each other (n = 12) display two peaks (red dotted lines) around 0 ms: at −1 ms and at +1 ms. Gray lines are cross-correlograms from individual pairs; the black trace is the mean of all 12 pairs.

(D) Mean cross-correlogram as indicated in (C), but computed with 0.1 ms time bins revealing that the majority of spike time correlations occur between 0 and ±2 ms.

(E) Computing the percentage of spikes that fall within distinct time lag windows across all pairs located <100 μm distance from each other reveals that 30% ± 3% of all spikes in WT mice are correlated within 5 ms of a spike in its paired neuron.

(F) Recordings of Golgi cell pairs at various inter-somatic distances indicated that the degree of correlation (defined as the maximal z score within a ±5 ms time lag window) decreases with distance. Gray areas in (B)–(D) and (F) indicate confidence interval (z scores between −2 and +2).

2000; Dugué et al., 2009; Ostojic et al., 2009; Pfeuty et al., 2003; Vervaeke et al., 2010) can occur depending on synaptic connectivity, spike shape, and intrinsic currents. Electrical coupling between Golgi cells has been proposed to synchronize Golgi cell networks at preferred oscillatory frequencies (Dugué et al., 2009) or to desynchronize oscillatory activity patterns under conditions of sparse mossy fiber excitation (Vervaeke et al., 2010). However, electrical coupling between Golgi cells, like electrical coupling between other interneuron types, has predominantly been studied in vitro, in isolation of spontaneous or sensory-evoked synaptic activity. The functional role of electrical coupling has not yet been directly studied in vivo, where circuitry is intact, background synaptic activity is high, and the synaptic inputs conveying sensory information can be stimulated directly.

We have therefore investigated the functional role of electrical coupling in the Golgi cell network in anaesthetized mice in vivo. Using targeted dual patch-clamp recordings from Golgi cells, we reveal that nearby Golgi cells display robust correlated activity with millisecond precision that is dependent on electrical coupling. Electrical coupling mediates this precisely correlated activity by the cooperative effect of two mechanisms: by equalization of the subthreshold membrane potential and by direct spike-to-spike transmission via spikelets. This precisely correlated activity is further enhanced when sensory stimuli are used to evoke spiking, even though spikes driven by sensory input display broad temporal variation due to the integration of sensory-evoked synaptic inputs. These results suggest a role for precisely correlated activity in Golgi cells in cerebellar function and provide a template for understanding correlated activity in electrically coupled interneuron networks across the mammalian brain.

RESULTS

Neighboring Golgi Cells Display Correlated Activity with Millisecond Precision

To establish whether Golgi cells exhibit correlated activity in vivo, two-photon guided targeted loose cell-attached recordings (Figure 1A) were made from Golgi cell pairs in mice anesthetized with ketamine/xylazine. Golgi cells fired spontaneously at 2.1 ± 0.3 Hz (mean ± SEM, n = 50 cells) without obvious rhythmicity (C.V. = 1.2 ± 0.1). This spontaneous activity is driven in part by synaptic input, as voltage-clamp recordings from Golgi cells showed a high level of continuous background synaptic activity, with EPSCs and IPSCs occurring at rates of 73 ± 12 and 64 ± 11 Hz, respectively (see Figures S1A–S1D, available online). Normalized cross-correlations of spike times in Golgi cell pairs were computed

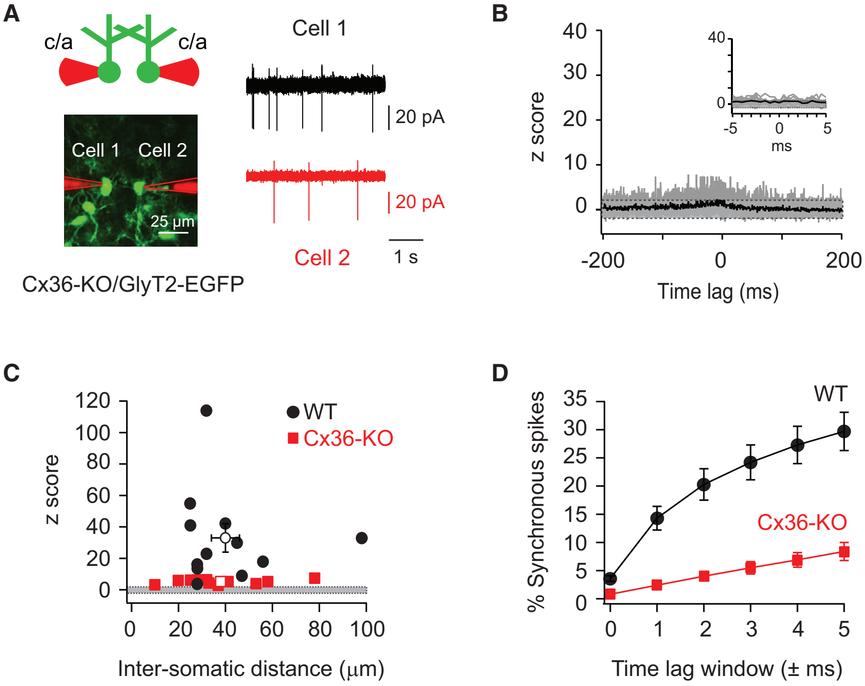

Figure 2. Precisely Correlated Activity Is Lacking in Cx36-KO Mice

(A) Recording configuration for measuring correlated activity between Golgi cells in a mouse line in which connexin36 is knocked out and glycinergic neurons express EGFP (Cx36-KO/GlyT2-EGFP, left). Dual loose-patch recordings were performed to determine spike timing in two neighboring Golgi cells (right).

(B) The cross-correlograms of all pairs recorded in Cx36-KO/GlyT2-EGFP mice lack a central peak around zero, indicating a lack of strong and precisely correlated activity. Gray lines are cross-correlograms from individual pairs; the black trace is the mean over all 12 pairs. The inset (right) displays the individual (gray lines) and mean (black line) cross-correlograms (0.5 ms bins) from −5 to +5 ms for all pairs ($n = 12$).

(C) Summary of maximal z scores determined from cross-correlograms of spike trains between Golgi cells pairs recorded with <100 μm intersomatic distance reveal strongly reduced degrees of correlated activity in Cx36-KO animals (mean = 5.0 ± 0.4, $n = 12$; red solid squares) compared to pairs in wild-type animals (mean = 33.1 ± 8.5, $n = 12$; black solid circles, $p = 0.001$).

(D) Computing the percentage of spikes within time lag windows between 0 and 5 ms across spike trains in all pairs reveals that 30% ± 3% of all spikes in WT mice are correlated within 5 ms. Only 8% ± 2% of all spikes across all spike trains in pairs in the Cx36-KO/GlyT2-EGFP mice displayed correlated activity with similar precision ($n = 12$, $p < 0.0001$). Gray areas in (B) and (C) indicate the confidence interval (z scores between −2 and +2).

by subtracting the mean cross-correlogram of 30 shuffled spike trains for each channel from the raw cross-correlogram and then dividing by the SD of the mean shuffled cross-correlogram. This normalization method controls for chance effects and normalizes for changes in firing rate between cells and pairs. The resulting normalized cross-correlograms exhibited prominent peaks around 0 ms time lag (Figures 1B and 1C). In most pairs, two discrete peaks were present—one at −1 ms and one at +1 ms (Figure 1C)—suggesting that spikes can either precede or follow a spike from a neighboring Golgi cell with millisecond precision. Histograms with 0.1 ms time bins showed that the majority of correlated activity occurred between 0 and ±2 ms (Figure 1D). To further quantify the precision of correlated activity, we determined the percentage of correlated spikes that occurred within time windows ranging between 0 to 5 ms. This revealed that 30% ± 3% of all spikes in a spike train occurred within ±5 ms of a spike from its paired neuron ($n = 12$ pairs, Figure 1E). Precisely correlated activity was dependent on intersomatic distance, with the strongest correlated activity found at intersomatic distances of less than 100 μm (Figure 1F). There was no significant relationship between the degree of correlated activity and the plane of orientation of Golgi cell pairs (Figures S1E and S1F), indicating that proximity alone determines the degree of millisecond precision-correlated activity, rather than activation by shared parallel fiber inputs (Volny-Luraghi et al., 2002; Vos et al., 1999a).

Electrical Coupling Is Essential for Precisely Correlated Activity

To test the contribution of electrical coupling to precisely correlated Golgi cell activity we crossed connexin36 knockout (Cx36-KO) mice with GlyT2-EGFP mice (see Supplemental Experimental Procedures). Connexin36 is both necessary and sufficient for mediating electrical coupling between Golgi cells (Vervaeke et al., 2010). We verified that the connexin36 gene was disrupted in the crossed mouse line and confirmed the results with immunocytochemistry (Experimental Procedures; Figures S2A and S2B). Baseline firing rates and irregularity of spiking in the Cx36-KO/GlyT2-EGFP mice were similar to those in wild-type GlyT2 mice (2.7 ± 0.5 Hz; C.V. = 1.2 ± 0.2, $n = 19$; $p = 0.06$ and $p = 0.13$, Figure S2C). Strikingly, though, paired recordings (Figure 2A) revealed a lack of precisely correlated activity in the Cx36-KO/GlyT2-EGFP mice: cross-correlations of spike times lacked peaks around 0 ms time lag (Figure 2B), and the mean maximal z score in the ± 5 ms time lag window around zero (5.0 ± 0.4, $n = 12$ pairs) was significantly reduced compared to the mean maximal z score in wild-type GlyT2-mice (33.1 ± 8.5, $n = 12$ pairs, $p = 0.0002$, Figures 2B and 2C). In Cx36-KO mice, only 8% ± 2% of all spikes on a given spike train occurred within ±5 ms of spikes from its paired neighbor, significantly fewer than in wild-type mice (30% ± 3%, $p < 0.0001$, $n = 12$, Figure 2D). These results indicate that strong and precisely correlated activity requires functional electrical coupling via gap junctions between Golgi cells.

Slow Subthreshold Membrane Potential Correlations Mediated by Electrical Coupling

To determine the mechanisms by which electrical coupling controls precisely correlated activity in vivo, dual whole-cell recordings were made from neighboring Golgi cells (Figure 3A). Most neighboring Golgi cells exhibited direct bidirectional electrical

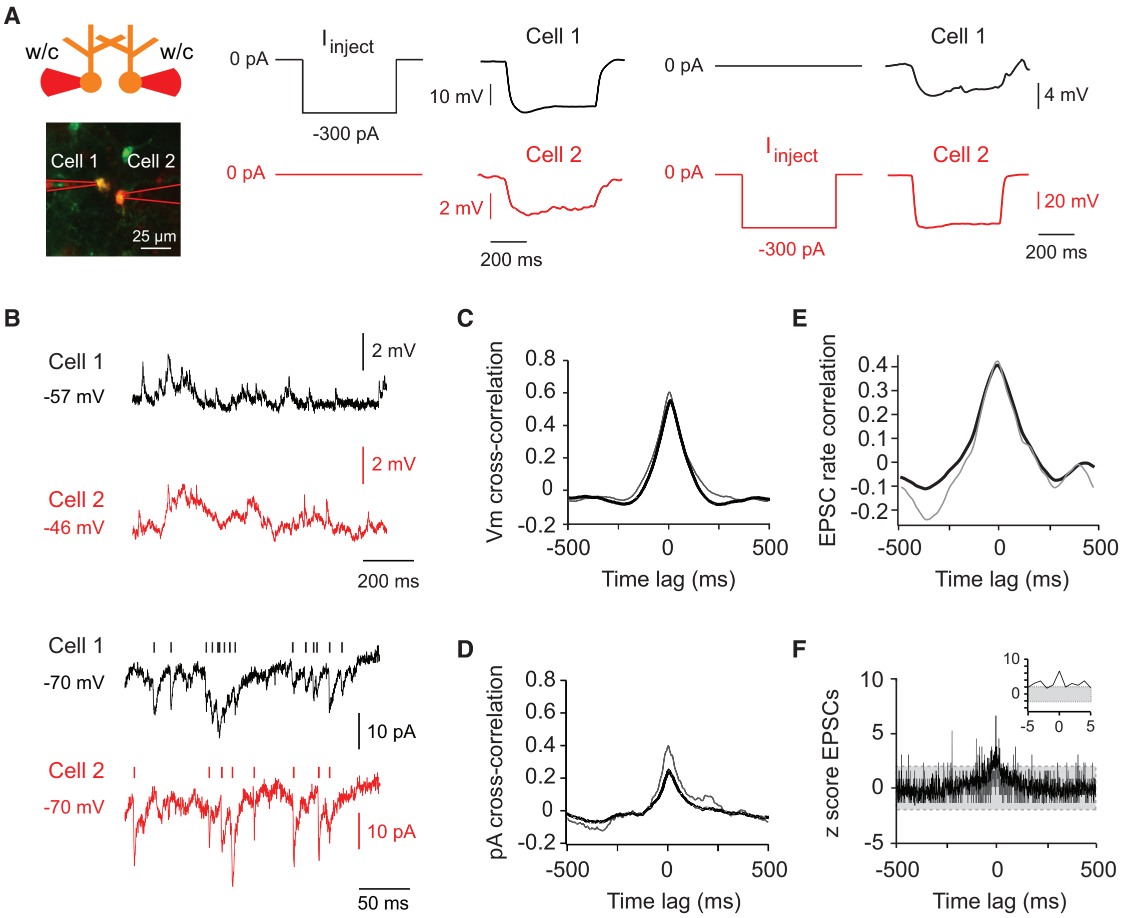

Figure 3. Dual Recordings Reveal Slow Subthreshold Correlations in Membrane Potential between Coupled Cells

(A) Recording configuration for dual whole-cell recordings between Golgi cells (left). Two nearby (<100 µm) Golgi cells were targeted, and membrane potentials or synaptic currents were recorded using dual whole-cell (w/c) recordings in current- and/or voltage-clamp mode. Consecutive hyperpolarizing current injections in one of two cells were used to determine electrical coupling between the two Golgi cells (middle and right). The coupling coefficient for this pair was 9%, averaged over both directions.

(B) Example traces of a dual whole-cell recording in a coupled Golgi cell pair. Membrane potential fluctuations were recorded in current-clamp mode (top traces), and membrane currents were recorded in voltage-clamp mode (bottom traces). The occurrences of identified individual EPSCs are indicated above the voltage-clamp traces (vertical lines).

(C) Cross-correlograms (on a scale of −1 to +1) computed from membrane voltage fluctuations revealed slow membrane potential correlations. The gray trace is the cross-correlation from the example traces shown in (B), and the black trace is the average cross-correlation across five pairs.

(D) Cross-correlograms computed from membrane current fluctuations also revealed a slow but less substantial correlation compared to the cross-correlation of voltage fluctuations. The gray trace is the cross-correlation from the example traces shown in (B), and the black trace is the average cross-correlation across three pairs.

(E) Cross-correlations of EPSC rates computed by counting synaptic event times in 128 ms bins. The gray trace is the cross-correlation of EPSC rates in a single pair. The black trace is the mean cross-correlation across three pairs of coupled neurons.

(F) The mean normalized cross-correlogram (1 ms bins) of the event times of individual EPSCs displays a small significant (z score of >2) peak around 0 time lag (inset). The gray trace is the cross-correlation from the example traces shown in (B), and the black trace is the average cross-correlation across three pairs. Gray area indicates the confidence interval (z scores between −2 and +2).

coupling in vivo, with a coupling coefficient of 5% ± 2% (n = 8; four pairs with detectable coupling out of five pairs). Spontaneous membrane potential fluctuations in coupled cells (Figure 3B, upper traces) exhibited slow positive membrane potential correlations (mean peak of 0.6 ± 0.1 on a scale of −1 to +1, and mean width between the negative inflection points of the cross-correlation peak = 474 ± 71 ms, or ~2 Hz, n = 5, Figure 3C), which were absent in a noncoupled pair (Figure S3). In voltage clamp mode, spontaneous current fluctuations (Figure 3B, bottom traces) exhibited slow correlations on a similar timescale to the spontaneous voltage fluctuations (Figure 3D), but with a less prominent peak than seen for voltage fluctuations (mean of 0.3 ± 0.1, n = 3). In voltage clamp, synaptic currents are largely isolated from the effects of electrical coupling between the recorded neurons, as no current will flow between gap junctions as a result of differences in membrane potential between the

two cells. The reduced correlation seen in voltage clamp therefore suggests that electrical coupling strongly contributes to the substantial cross-correlation of membrane voltage observed in current clamp. Given that gap junctions between Golgi cells act as low-pass filters with a cut-off frequency of ~10 Hz (Dugué et al., 2009) and that the degree of correlated activity is strongly reduced when isolating synaptic inputs from the effects of electrical coupling in voltage clamp mode, we conclude that electrical coupling plays a prominent role in mediating slow membrane potential correlations between Golgi cells.

The raw data in both current and voltage clamp (Figure 3B) show that the rate of EPSPs/EPSCs fluctuates and that some of these fluctuations occur at the same time in both neurons. To quantify this, we detected EPSCs in both cells (Figure 3B, bottom traces) and determined the cross-correlation of the rate of EPSCs by binning EPSC coincidences in 128 ms bins in pairs of Golgi cells. EPSCs showed a peak EPSC rate cross-correlation of 0.4 ± 0.1 (Figure 3E, n = 3), suggesting that the EPSC rate is comodulated between two coupled neurons over the timescale of ~100 ms. This slow comodulation of EPSC rate is likely to contribute to the slow correlations of input current measured in voltage clamp shown in Figure 3D, and in turn to the slow membrane potential correlations observed during voltage recording (Figure 3C). The slow membrane potential correlations resulting from both membrane potential equalization via coupling and slow comodulation of EPSC rates increase the probability that coupled neurons depolarize together toward action potential threshold.

We next computed cross-correlograms of individual EPSC event times and determined the percentage of precisely synchronous EPSCs between coupled neurons. Only a small fraction of the EPSCs (4% ± 1%, n = 3) computed from cross-correlations were precisely synchronous (defined as occurring within ±0.5 ms), as reflected in the small peak (z score of 6 ± 4) in the normalized cross-correlogram at 0 ms (Figure 3F). These results indicate that precisely synchronous or common EPSCs are rare, with the slow tails in the EPSC cross-correlogram reflecting overall periods of EPSC rate correlation at a slower timescale (compare with Figure 3E). The fact that precisely synchronous EPSCs are rare suggests that they are unlikely to drive the majority of spikes that exhibit correlations with millisecond precision.

Spikes Trigger Depolarizing Spikelets via Gap Junctions

Slow subthreshold membrane potential correlations mediated by coupling may be permissive for correlated activity, but they do not explain the millisecond precision of the correlated activity we observed. We therefore set out to test whether transmission of a spike via gap junctions and the resulting "spikelet" currents can trigger millisecond precision correlated activity. First, we examined the amplitude of spikelet currents and potentials by computing spike-triggered averages in simultaneous whole-cell and cell-attached recordings (Figure 4A). This revealed that prejunctional action potentials were associated with depolarizing junction potentials (0.4 ± 0.2 mV, n = 9; Figure 4B) and currents (8 ± 2 pA, n = 6; Figure 4C) in the postjunctional cells. These spikelet voltages and currents followed the spikes in the coupled cell with a mean peak latency of 1.1 ± 0.1 ms and 0.6 ± 0.1 ms, respectively. Importantly, spikelets were absent (mean depolarization = 0.05 ± 0.01 mV in first 1.5 ms following cell-attached spike versus 0.4 ± 0.2 mV, p = 0.02) in pairs that did not appear to be electrically coupled given that they lacked significant precisely correlated activity (mean maximal z score = 5 ± 1, n = 3, Figure S4).

Spikelet potentials are small, and thus, to determine how spike-to-spike transmission across gap junctions may depend on the membrane potential in the postjunctional cell, we analyzed the membrane potential 5 ms prior to a correlated spike triggered by a cell-attached spike in its coupled neighbor and compared it to the membrane potential on those occasions in which a correlated spike failed to be induced (Figure 4D). This revealed that correlated spikes were triggered when the absolute membrane potential was more depolarized and closer to threshold (pre-V_m, –41.8 ± 1.4 mV for correlated spikes and –43.9 ± 1.7 mV for noncorrelated spikes, n = 5, p = 0.01, Figures 4E and 4F). Furthermore, the membrane potential depolarized more in the 5 ms prior to a correlated spike compared to when spike-to-spike transmission failed (ΔV_m, 1.5 ± 0.4 mV for correlated spikes and 0.5 ± 0.1 mV for noncorrelated spikes, n = 5, p = 0.02, Figures 4E and 4F). Finally, even in episodes where no spikes were induced, the membrane potentials were on a depolarizing trajectory (ΔV_m was positive, Figures 4E and 4F), suggesting that both coupled neurons are depolarizing prior to the generation of a spike in one of the two cells, as predicted by the slow subthreshold membrane potential correlations (Figure 3). These data suggest that spike-to-spike transmission via gap junction-mediated spikelets in vivo occurs when the membrane potential in the postjunctional cell is on a depolarizing trajectory and close to threshold.

Triggered Spikelets Can Drive Precisely Correlated Postjunctional Spikes

To test directly whether spikelets can trigger postjunctional spikes, we triggered spikes by current injection in one cell of a coupled pair (recorded in whole-cell mode) while recording the spiking of the other cell (in cell-attached mode). A train of spikes in the prejunctional cell led to a robust increase in spiking of the postjunctional cell (166% ± 82% increase in firing rate, n = 6; Figure 5A, left and middle). Cross-correlograms of pre- and postjunctional spikes revealed that spikes occurred with millisecond (±1 ms) precision (Figure 5A, right) comparable to spontaneous correlated spiking (Figure 1). To separate the effects of prejunctional membrane depolarization and the triggered action potentials, we next examined spike-to-spike transmission across gap junctions by using brief current pulses to trigger single spikes in the prejunctional cells (Figure 5B, left). Single induced action potentials were able to trigger precisely timed spikes in the postjunctional cells on 5% of the trials (Figure 5B, middle). The postjunctional spikes were driven with millisecond precision by the prejunctional spike, with a single peak in the cross-correlograms of induced whole-cell spikes and spikes in its coupled neighbor at +1 ms (Figure 5B, right).

Spike-to-spike transmission across the electrical junction was state dependent, however, as shown by the probability of spike transmission in different conditions. Spikes triggered by current injection in one prejunctional cell had a lower transmission probability than spontaneous spikes (Figure 5C, black bars), presumably because spikes were triggered at random times, and could

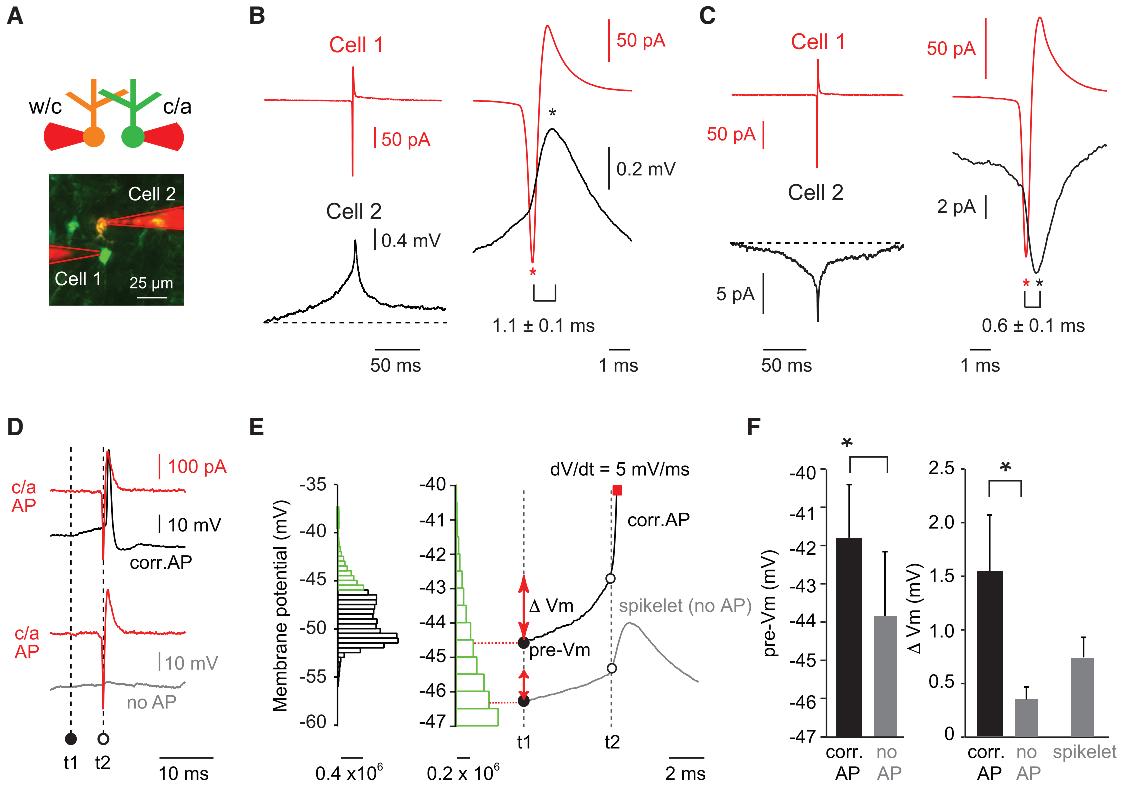

Figure 4. Triggering of Correlated Spikes by Spikelets Is State Dependent

(A) Recording configuration for measuring the effect of single action potentials on spike timing. Two nearby (<100 μm) Golgi cells were targeted using two-photon guidance. One cell was recorded in cell-attached (c/a) mode (Cell 1), and the other cell was recorded in whole-cell (w/c) current-clamp mode (Cell 2).

(B) Dual cell-attached and whole-cell recordings during spontaneous correlated activity allowed us to characterize spike-triggered voltages or so called "spikelets" in postjunctional Golgi cells. Cell-attached (c/a) spikes (top red trace, left) from Cell 1 were used to characterize the mean spike-triggered voltage (lower black trace, left) in Cell 2. A fast spike-triggered event could be identified riding on top of a broader depolarization that outlasted the spike. On the right, an overlay of the mean cell-attached spike and the whole-cell spike-triggered voltage is shown. The fast spike-triggered "spikelet" always occurred after the peak of the cell-attached spike, and the mean time lag between the two events was 1.1 ± 0.1 ms. Data represent mean ± SEM of nine pairs.

(C) Same parameters as in (A) and (B), but for spike-triggered currents recorded in voltage-clamp mode. The mean time lag between the two events was 0.6 ± 0.1 ms. Data represent mean ± SEM of six pairs.

(D) The membrane potential in Cell 2 in the 5 ms prior (t2 − t1) to the induction of precisely correlated (<±5 ms) spikes (black trace) or during failures (gray trace) was analyzed using spike-triggered averaging of the cell-attached spikes in Cell 1 (red trace).

(E) Example recording showing on the left the membrane potential distribution (in counts of membrane potential data points recorded) of Cell 2, with the upper most depolarized range indicated in green. On the right, the mean V_m trajectories 5 ms prior (t1) to the peak of the cell-attached spike (t2) in correlated spikes (black trace) and during failures (gray trace) are mapped onto the depolarized subset of the membrane potential distribution (middle), which shows that correlated spikes occur when the membrane potential is most depolarized. In the case of failures (gray trace), a spikelet occurs immediately after t2. Pre-V_m is absolute membrane potential value at t1, and ΔV_m is the voltage difference between t1 and t2 (t1 = t2 − 5 ms). Red square marker indicates action potential threshold for this cell, defined as the point where dV/dt exceeds 5 mV/ms.

(F) Mean data for pre-V_m values (p = 0.01), ΔV_m (p = 0.02), and spikelet amplitude computed across n = 5 pairs.

therefore occur when the postjunctional cells may be far from threshold. In contrast, precisely correlated cell-attached spikes as a percentage of all cell-attached spikes increased during spike-injection experiments compared to spontaneous spiking conditions (Figure 5C, red bars). Similarly, the symmetry in spike coupling switched from being roughly 50:50 under spontaneous spiking conditions, to a situation where the triggered spikes nearly always preceded the postjunctional spikes (Figure 5D, black and gray bars). Notably, spike-to-spike transmission was unsuccessful in 3 pairs that lacked spontaneous precisely correlated activity (mean maximal z score = 5 ± 1, n = 3, Figure S5) and spikelets (Figure S4), confirming that electrical coupling is required for reliable spike-to-spike transmission. Together, these findings suggest the following mechanism: only if a combination of EPSC rate correlation and equalization of membrane potential via electrical coupling brings two coupled cells close to threshold, then a spike in one cell will lead to a spike in the other cell with a millisecond delay via a transmitted spikelet.

Cooperative Effect of Subthreshold Membrane Potential Equalization and Spikelets Drives Precisely Correlated Activity

To develop a more quantitative understanding of how synaptic input, subthreshold membrane potential correlation, and

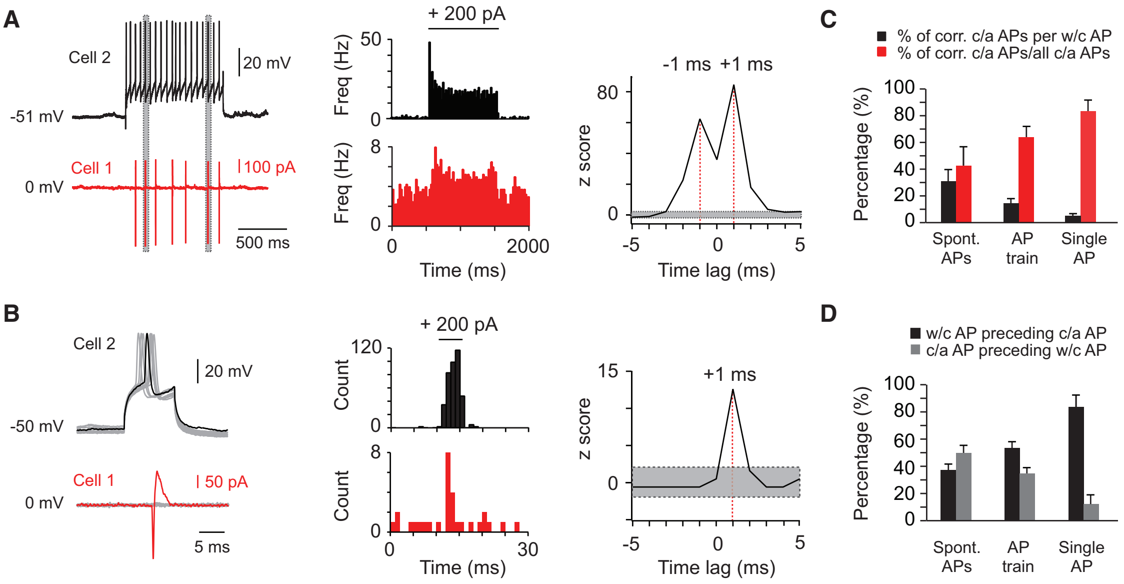

Figure 5. Induced Spikelets Can Drive Spike-to-Spike Transmission

(A) Example traces (left) from an experiment (same experimental configuration as in Figure 4) in which a (1 s) train of action potentials was induced in Cell 2 and the effect on spike timing was assessed in Cell 1. The poststimulus time histograms (PSTH, 20 ms bin, middle) of spiking summarizing results across multiple trials indicate that the firing rate was transiently increased in Cell 1 during the time that spikes were induced in Cell 2. Gray bars (left) indicate correlated spikes (<±5 ms). The cross-correlogram (1 ms bins, right) shows two peaks (red dotted lines) at −1 ms and +1 ms, similar to spontaneous correlated activity.

(B) Example traces (left) from an experiment where only a single action potential was induced in Cell 2. Across multiple trials, these single action potentials were capable of inducing correlated spikes in the coupled cell, despite a high failure rate (PSTHs, 1 ms bin, middle). The timing of correlated spikes in Cell 1 (±5 ms time lag window) also displayed millisecond precision, but spikes in Cell 1 always followed the spikes in Cell 2, resulting in a single peak at +1 ms (right).

(C) Summary graph displaying the percentage of correlated spikes (<±5 ms time lag) observed under three conditions: during spontaneous action potentials, during a stimulus induced train of action potentials, and during stimulus induced single action potentials. Black bars indicate the percentage of spikes in Cell 2 that induced a correlated spike in Cell 1. Red bars indicate the percentage of correlated spikes in Cell 1 of all spikes in Cell 1. Data represent mean ± SEM from six pairs.

(D) Summary graph displaying the symmetry of correlated spikes (±5 ms time window) across the three different experimental conditions. Black bars indicate the percentage of spikes in Cell 2 that precede spikes in Cell 1. Gray bars indicate the percentage of spikes in Cell 1 that precede the spikes in Cell 2. Data represent mean ± SEM from six pairs.

spikelets combine and interact to generate millisecond precision correlated activity, we constructed a generalized mathematical model of two electrically coupled neurons (Figures 6A and 6B). In this model, both neurons received a constant rate of in vivo-like EPSCs and IPSCs (Figure 6B; see also Supplemental Experimental Procedures) and the degree of precisely synchronous common input was set to 4%, as determined experimentally. Precisely synchronous common and independent inputs had equal amplitudes, which were set to be equivalent to those observed in the experimental data (Figure S1D; see also Supplemental Experimental Procedures). The model enabled us to turn off electrical coupling entirely, or to selectively turn off the subthreshold membrane potential correlation due to coupling and/or the spikelet. Furthermore, it allowed us to specifically turn off the depolarizing junction potential (DJP) or the hyperpolarizing junction potential (HJP) in each spikelet (Figure 6B). Under baseline conditions, spiking in these electrically coupled neurons displayed correlated activity with peaks in the cross-correlogram at ±1 ms (mean z score at ±1 ms = 36, z score at 0 ms = 3; Figures 6B and 6C, black trace), comparable to the experimentally observed correlated activity in Golgi cells in vivo. Turning off electrical coupling resulted in minimal correlated spiking (z score at 0 ms = 1; Figure 6C, red trace), qualitatively consistent with the experimental observations using the Cx36-KO mice (Figure 2B), and the idea that correlated spiking is not driven primarily by precisely synchronous EPSCs. Turning off the transmission of the spikelet while leaving subthreshold correlations due to coupling intact increased the degree of correlated activity somewhat (z score at 0 ms = 2, Figure 6D), but did not result in precisely correlated activity with peaks at ±1 ms. Turning off subthreshold correlations while turning on spikelet transmission, however, resulted in the emergence of ±1 ms peaks in the cross-correlogram, albeit with z scores lower compared to control (mean z score at ±1 ms = 24, z score at 0 ms = 3, Figure 6E). Together, these results suggest that subthreshold membrane potential correlations and spikelets cooperate to produce robust correlated activity with millisecond precision as seen experimentally.

Dissecting the effects of the DJP and HJP of the spikelets revealed that the DJP on its own causes the ±1 ms peaks (mean z score at ±1 ms = 17, z score at 0 ms = 1, Figure 6F), while the HJP on its own does not result in peaks at ± 1 ms, but in a peak at 0 ms with z score = 2 (Figure 6G), a value which is comparable to the z score at 0 ms in the case of subthreshold coupling only. The HJP also suppresses correlated firing,

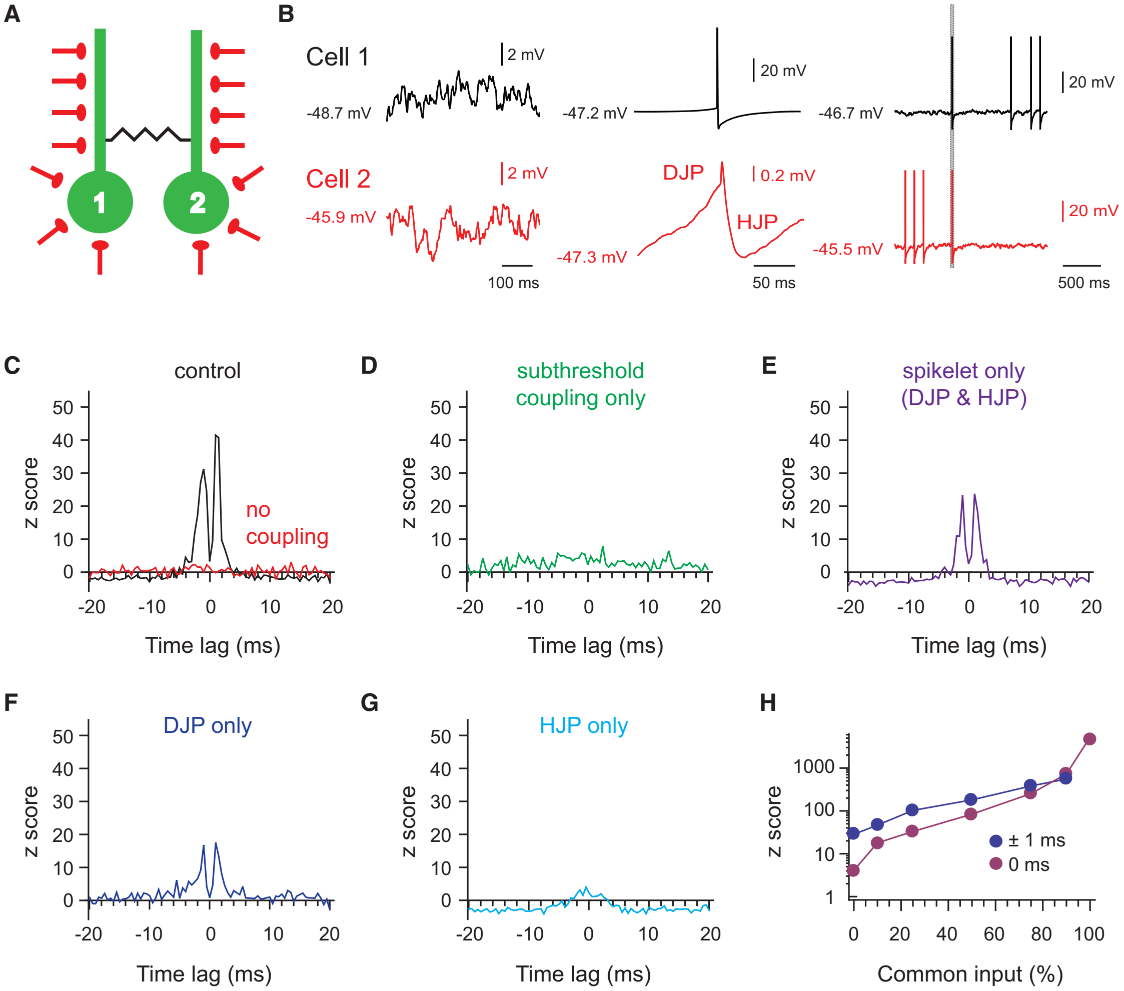

Figure 6. Cooperative Effect of Subthreshold Membrane Potential Correlations and Spikelet Transmission Drives Precisely Correlated Activity

(A) Schematic overview of the computational model in which two neurons are electrically coupled and both receive random synaptic input, 4% of which is common to both neurons (see Supplemental Experimental Procedures).

(B) Left panel, subthreshold membrane potential traces recorded at the soma. Center panel, an action potential was generated by membrane potential fluctuations crossing threshold (top) resulting in a transmitted spikelet potential (bottom), consisting of a depolarizing junction potential (DJP) and hyperpolarizing junction potential (HJP). In the model we were able to selectively turn off subthreshold membrane potential correlation due to coupling, and/or the spikelet potentials. Right panel, example traces of spiking in the two coupled model neurons. Gray bar indicates the occurrence of correlated spikes (<5 ms time lag).

(C) Cross-correlograms of spike timing in the two coupled neurons under control conditions and with gap junction coupling entirely off.

(D) Cross-correlogram with only subthreshold membrane potential correlation due to electrical coupling on, and with the transmission of spikelets off.

(E) Cross-correlogram with only spikelet transmission on, and no electrical coupling at subthreshold membrane potentials.

(F) Cross-correlogram with only DJP transmission on.

(G) Cross-correlogram with only HJP transmission on.

(H) Degree of correlated activity as a function of increasing degrees of precisely synchronous common input. Blue line is the average z score over the two peaks in the cross-correlograms at ±1 ms. The purple line is the z score at the 0 ms time bin representing the degree of correlated activity as a function of precisely synchronous common synaptic input.

decreasing the cross-correlations beyond ±2 ms and rendering the z scores negative (Figure 6G). Next, we set out to determine how increasing levels of precisely synchronous common synaptic input affects correlated activity mediated by electrical coupling. Increasing the degree of precisely synchronous input enhances the peak at 0 ms, but only starts dominating spike timing over spikelet-induced peaks at ±1 ms when the degree of precisely synchronous input is >80% (Figures 6H and S6).

The prejunctional spike used in the model was based on the spike shape observed experimentally in Golgi cells, with a relatively long-lasting and deep AHP (17 mV amplitude and a duration of 14 ms defined as the full width at half-maximum, or FWHM, of the AHP). Given that the AHP has been predicted to cause desynchronization of neurons (Vervaeke et al., 2010), we modified the model to use a spike shape with a smaller and faster AHP (8 mV and 9 ms FWHM duration), more similar to cortical

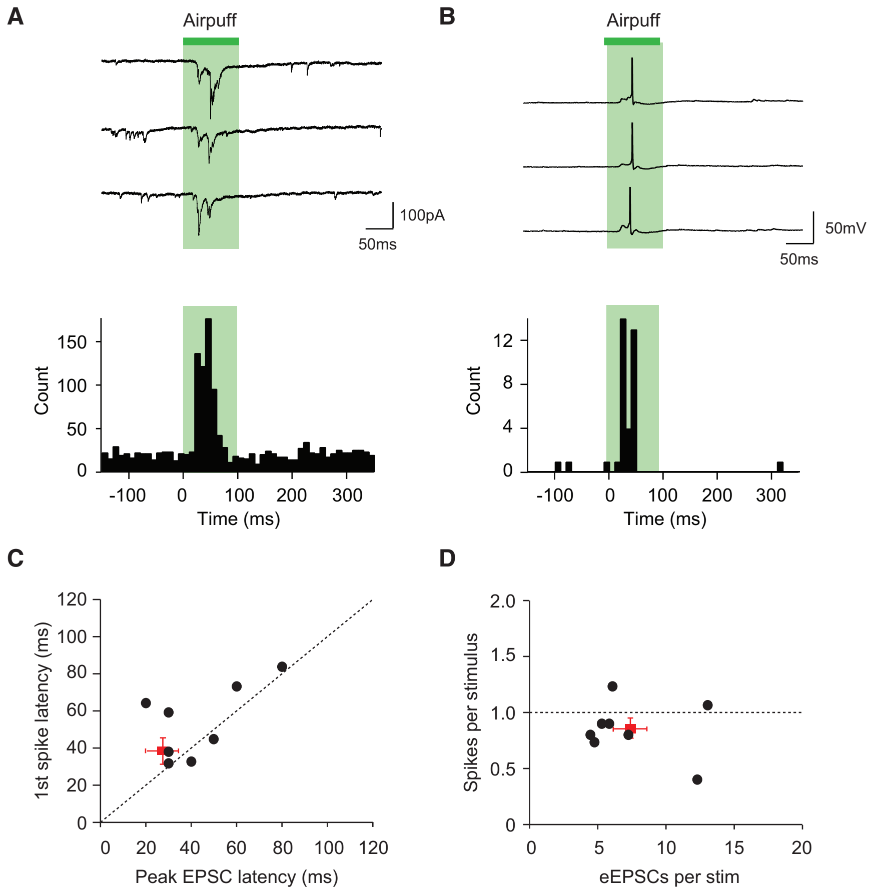

Figure 7. Sensory-Evoked Synaptic Integration and Spike Induction in Golgi Cells

(A) Whole-cell patch clamp recording from a single Golgi cell showing that sensory stimulation of the peri-oral region and/or whisker pad evokes a burst of excitatory postsynaptic currents (EPSCs) (green area indicates duration of airpuff). Three consecutive trials and the peristimulus time histogram (PSTH, 10 ms bins) across trials are shown.

(B) Spikes evoked by the same stimulus in the same cell as in (A). Three consecutive trials are shown, and one sensory-evoked spike is seen per trial in this cell. The PSTH of all evoked spikes across trials (10 ms bins) is shown below the traces.

(C) Plotting the spike latency against the peak latency of the burst of EPSCs across cells reveals that Golgi cells spike after the burst of evoked EPSCs reaches its peak. Red square indicates mean ± SEM (n = 8 cells).

(D) Golgi cells on average produce one spike per sensory stimulation, in response to a ranging number of evoked EPSCs (eEPSCs) within a burst. Red square indicates mean ± SEM (n = 8 cells).

interneuron spikes. The degree of correlation as a function of differing degrees of precisely synchronous common input was qualitatively similar with both spike shapes (Figures S6A and S6B), indicating that millisecond precision correlated activity is reliably generated for a range of AHP amplitudes and durations. Finally, the model allowed us to study correlated spiking as a function of degree of precisely synchronous common input in the complete absence of gap junction coupling. This showed that for nonzero degrees of common input, correlated activity displays peaks at 0 ms, indicating perfect synchrony in spike timing due to synaptic inputs alone (Figure S6C). This result indicates that peaks at ±1 ms in spike time cross-correlations are caused by electrical coupling, not common synaptic inputs, and are thus a useful signature of electrical coupling in cells exhibiting correlated activity.

Sensory Stimuli Induce Spikes via Integration of Bursts of EPSCs

Finally, we determined the degree and temporal characteristics of correlated activity in coupled Golgi cells in response to sensory stimulation. Since mossy fiber terminals conveying sensory stimuli terminate profusely in the granule cell layer and may simultaneously activate many coupled Golgi cells, precisely correlated activity between Golgi cells may be enhanced by a sensory stimulus. Alternatively, sensory stimuli might desynchronize or lessen the degree of correlated activity (Vervaeke et al., 2010). Furthermore, the temporal precision of correlated activity may broaden as a result of heterogeneous sensory-evoked responses in individual cells. We first characterized the synaptic inputs driven by sensory stimulation in single Golgi cells using whole-cell voltage clamp recordings. Sensory stimulation induced a burst of high-frequency excitatory inputs in Golgi cells, with a mean latency to the peak of the EPSC burst of 28 ± 7 ms (n = 8). In current-clamp recordings, this sensory-evoked synaptic input triggered single action potentials in most trials, with a mean latency of 35 ± 6 ms (n = 10, Figures 7A and 7B). The latencies of the peak of the EPSC burst and the resulting spikes in the same cells were highly correlated (Figure 7C), with the number of individual EPSCs leading to a spike ranging from 4 to 14 (Figure 7D). Thus, under these experimental conditions, Golgi cells need to integrate a minimum of 4–14 individual EPSCs before reaching action potential threshold.

Enhanced Correlated Activity with Millisecond Precision during Sensory Stimulation

In simultaneous recordings from pairs of Golgi cells, sensory stimulation triggered spiking in both cells (latency of 32 ± 2 ms; n = 5 pairs, Figures 8A and 8B), resulting in correlated activity that was more pronounced than spontaneous correlated activity in the same pairs (mean z score 91 ± 20 versus 57 ± 15 prestimulus, $p < 0.01$, and 57 ± 17 poststimulus, $p < 0.01$, n = 5; Figures 8C and 8D). Accordingly, the percentage of correlated spikes (defined as within ±5 ms) of all spikes was higher during sensory stimulation (42% ± 6%, n = 5) compared to that during spontaneous correlated activity (27% ± 4% prestimulus, $p < 0.001$ and 29% ± 4% poststimulus, $p < 0.01$, respectively, n = 5; Figure 8E). The bidirectional symmetry of correlated spiking

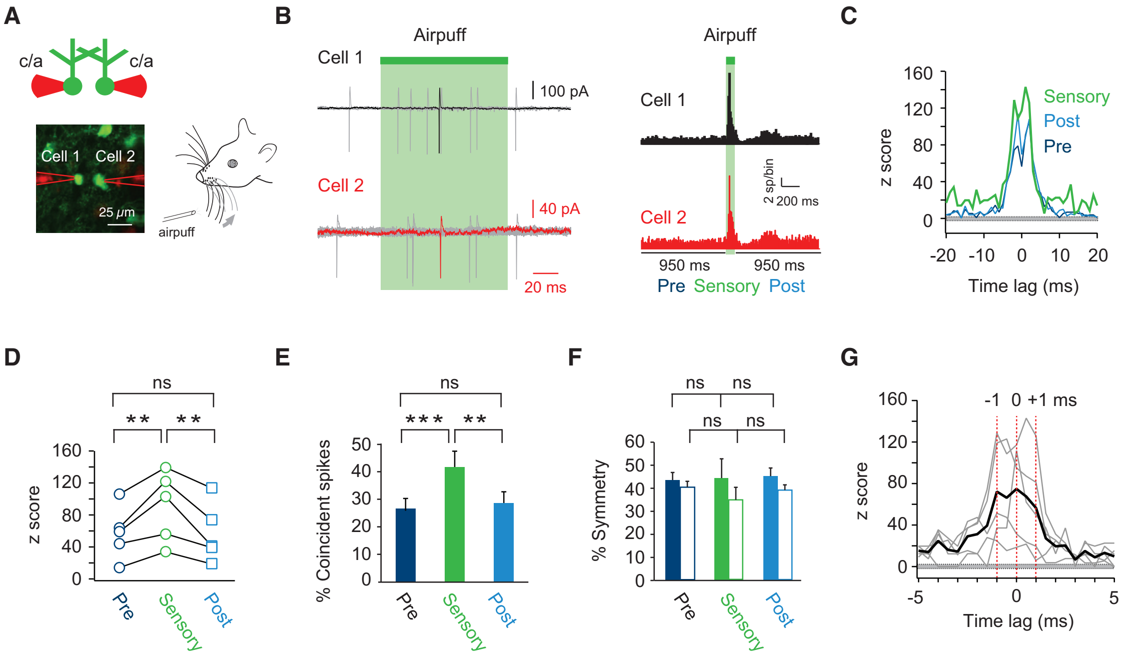

Figure 8. Enhanced Correlated Activity with Millisecond Precision during Sensory Stimulation

(A) Recording configuration for measuring sensory evoked correlated activity between Golgi cells in GlyT2-EGFP mice. Two nearby Golgi cells were targeted and spike trains were recorded using dual loose cell-attached (c/a) recordings (left). Sensory stimulation was provided by an air puff to the peri-oral region and/or whisker pad (right).

(B) Ten overlaid example traces on the left reveal the occurrence of sensory-induced spikes in response to the air-puff. One trial is highlighted (in black and red) on both spike trains. Green bars indicate the duration of the air puff (100 ms). Histograms on the right summarizing the results across all trials reveal clear sensory responses in both cells in this pair. The degree of correlated activity was computed 950 ms preceding the sensory stimulus (Pre), during the 100 ms stimulus (Sensory), and in the 950 ms following the stimulus (Post).

(C) Cross-correlograms of the example pair shown in (B). The data indicate that the sensory-evoked stimulus significantly enhances prestimulus correlated activity, which returns to baseline levels during the poststimulus time period.

(D) Summary graph of z score values before, during, and after sensory stimuli across all pairs (n = 5).

(E) Summary bar graph displaying the percentage of correlated spikes before, during, and after sensory stimuli across all pairs (n = 5).

(F) Summary bar graph displaying the symmetry of correlated spikes before, during, and after sensory stimuli across all pairs (n = 5). Solid bars reflect Cell 1 preceding Cell 2, and open bars reflect Cell 2 preceding Cell 1.

(G) Individual (gray lines) and mean (black line) cross-correlograms (0.5 ms bins) of sensory evoked correlated activity indicating a multipeaked structure around zero time lag with peaks at −1, 0, and +1 ms (red lines).

between coupled Golgi cells did not change during sensory evoked stimuli (p = 0.96 and p = 0.33 for both directions, Figure 8F). Given that dominant inputs to only one cell would cause asymmetry due to the fact that electrical coupling is bidirectional, this suggests that both cells were driven approximately equally by the sensory stimulus.

The sensory-evoked cross-correlograms exhibited peaks at −1, 0, and +1 ms (Figures 8C and 8G), as in spontaneous spiking, suggesting that while the peak at 0 ms is larger during sensory stimuli, electrical coupling still strongly determines spike timing during sensory stimuli. However, we also observed a broader foot in the cross-correlogram of the sensory-evoked spikes, representing enhanced correlations at longer time lags (Figure S7A). To determine whether the variability of evoked spiking in individual cells underlies this increase in slower time correlations, we shuffled spike times across sensory trials which revealed broad peaks around zero (Figures S7B and S7D), similar to the autocorrelations of spike times across trials for each cell in a pair (Figure S7C). Across pairs, these shuffled cross-correlograms revealed broad correlations lacking sharp peaks around 0 ms, suggesting that the main effect of sensory stimuli is to enhance temporal correlations at longer time lags (Figure S7D). Together, these data suggest that sensory stimuli enhance the degree of correlated spiking between pairs of Golgi cells and enhance temporal correlations at longer time lags. Nevertheless, the temporal precision of the correlated activity that is ensured by electrical coupling still dominates the spike timing of coupled Golgi cells.

DISCUSSION

We report the first demonstration of correlated activity with millisecond precision in an identified electrically coupled interneuron network in vivo. Electrical coupling is essential for the temporal precision of this correlated activity, and acts in a dual, cooperative manner: by equalizing slow subthreshold

membrane potential depolarizations and by transmitting fast depolarizing spikelet currents. Sensory stimuli evoke bursts of synaptic inputs that evoke spikes with variable timing across trials in individual cells, but they enhance the degree of precisely correlated activity. Since many interneurons in the mammalian brain are electrically coupled (Connors and Long, 2004; Galarreta and Hestrin, 2001), our results provide a mechanistic understanding of how electrical coupling can orchestrate millisecond-scale correlated activity under conditions of spontaneous and sensory evoked synaptic activity.

Electrical Coupling Employs Two Cooperative Mechanisms to Ensure Precisely Correlated Activity of Golgi Cell Firing In Vivo

Correlated activity with millisecond precision has not previously been reported in the Golgi cell population in vivo because of the difficulty of recording unambiguously from neighboring Golgi cells in the intact brain. Previous findings of weaker, less precisely correlated activity (half-width of the cross-correlogram peak of ~29 ms) among Golgi cells (Vos et al., 1999a), presumably mediated by common parallel fiber input, were made with electrode spacings 300–2,100 μm apart, well beyond the ~200 μm span of the Golgi cell dendritic tree that defines the spatial dimension of the precisely correlated activity that we have observed. Our dual whole-cell recordings between coupled Golgi cells demonstrate that gap junctional coupling enables precisely correlated activity via the cooperative effect of two mechanisms. First, electrical coupling helps to equalize subthreshold membrane potentials, effectively allowing the neurons to share synaptic input (Vervaeke et al., 2012) by transmitting slow membrane potential fluctuations across the junction. Second, when both cells are driven close to threshold, then a spike triggered in one cell is able to trigger a spike in a coupled cell by transmission of a spikelet across the gap junction. These two mechanisms work together to ensure precisely correlated activity: the efficacy of the spike-to-spike transmission via the spikelet is dependent on the membrane potentials of both cells depolarizing together so that both cells are near threshold at the same time.

Our simulations using a generalized mathematical model of dendritic electrical coupling reveal further insights into the biophysical mechanisms of correlated activity. While the spikelets are responsible for the millisecond precision of correlated spiking, subthreshold membrane potential equalization due to coupling acts cooperatively with spike-to-spike transmission to ensure the robust degrees of correlated spiking as observed experimentally. Moreover, they show that precisely synchronous synaptic inputs to Golgi cells are only likely to dominate millisecond correlated spiking if they represent more than 80% of all inputs. This emphasizes the dominant role of electrical coupling in determining precisely correlated activity of Golgi cells. Our simulations furthermore reveal that these effects are largely independent of spike shape. The reduced model used in the simulation exhibits passive dendrites, as has been experimentally observed for Golgi cell dendrites (Vervaeke et al., 2012, but see Rudolph et al., 2015), like those of other types of interneurons (Hu et al., 2010). However, in electrically coupled cell types with active dendrites, spikelets may further summate with dendritic spikes to drive spiking even more effectively (Trenholm et al., 2014).

Recent experimental work in cerebellar slices (Dugué et al., 2009) and theoretical work (Chow and Kopell, 2000; Ostojic et al., 2009) have suggested that the low-pass filter represented by the gap junction conductance and the capacitance of the postjunctional cell should favor transmission of the spike afterhyperpolarization over the faster depolarizing spikelet itself. This in turn can lead to hyperpolarization and desynchronization of coupled neurons (Vervaeke et al., 2010). However, under our in vivo conditions, the spikelet has a predominantly excitatory effect, with its afterhyperpolarization appearing to be less functionally relevant than under in vitro conditions. While a hyperpolarization follows the peak of the spikelet in vivo (Figure 4B), the membrane potential does not drop below its value just prior to a spike. The importance of the depolarizing junction potential of the spikelet may be partly explained by voltage-dependent boosting of the spikelet amplitude (Curti and Pereda, 2004; Dugué et al., 2009; Mann-Metzer and Yarom, 1999) by the more depolarized membrane potentials observed in vivo. More importantly, however, both the experimentally observed and the simulated spikelet occur on a slow depolarizing ramp, which we argue is due to correlations in the ongoing synaptic input rate in vivo and the subthreshold membrane potential equalization between coupled neurons. Superimposed on this ramp, the hyperpolarizing junction potential due to the afterhyperpolarization following prejunctional spikes does not lead to a strong hyperpolarization below pre-spike baseline. Electrical coupling is usually studied in isolation of synaptic activity, while in vivo, under ketamine/xylazine anesthesia, we report high frequencies of spontaneous excitatory and inhibitory activity (73 ± 12 and 64 ± 11 Hz for EPSCs and IPSCs, respectively). In the awake animal, synaptic activities may be even higher, particularly during locomotion (e.g., see Powell et al., 2015), and thus spikelet afterhyperpolarizations may be even more strongly affected by enhanced degrees of synaptic excitation.

Comparison with Other Brain Areas

The mechanism for millisecond precision correlated activity among electrically coupled interneurons we describe is simple and robust, and is likely to be widespread across the mammalian brain, given that electrically coupled interneurons can be found in many brain areas (Connors and Long, 2004; Galarreta and Hestrin, 2001) and have been shown to drive correlated activity in vitro (Galarreta and Hestrin, 1999; Gibson et al., 1999). Early single-unit recordings from unidentified interneurons in somatosensory cortex in vivo reported the occurrence of precisely correlated activity superimposed on a broader, slow correlated activity of tens of milliseconds (Swadlow et al., 1998), indicating that cortical interneurons can display both precisely and slow correlated activity. More recently, optogenetic identification of specific interneuron classes in combination with unit recordings in the prefrontal cortex (Kvitsiani et al., 2013) revealed that a subpopulation of pairs of parvalbumin-positive interneurons, but not somatostatin-positive units, could display millisecond precision correlated activity with each other and with some unidentified units. Dual patch-clamp recordings between pairs of interneurons in layer 2/3 of cerebral cortex revealed slow subthreshold

membrane potential synchrony but correlated spike activity that was relatively imprecise (Gentet et al., 2010). This may be due to the fact that the recorded interneurons, which were not assigned to specific interneuron classes, may not have been electrically coupled, given that interneuron electrical coupling is specific to interneuron type (Connors and Long, 2004; Galarreta and Hestrin, 2001). Moreover, the slow subthreshold membrane potential synchrony was also observed between pairs of pyramidal cells and pairs of pyramidal cells and interneurons in the same preparation (Gentet et al., 2010; Lampl et al., 1999; Poulet and Petersen, 2008), suggesting that it arises from propagating waves of activity. More recent extracellular recordings of pairs of differing putative interneuron type in hippocampus revealed millisecond precision synchrony, arguing against a sole role of coupling and for a role of inhibition in mediating this synchrony (Diba et al., 2014). Thus, whether the mechanism we describe also drives correlated activity with millisecond precision among coupled cortical and hippocampal interneuron populations in vivo remains to be established.

Nevertheless, the importance of electrical coupling in cortical circuits has long been recognized at the level of network activity, where it contributes to and enhances oscillatory activity. For example, elimination of electrical coupling in Cx36-KO mice has been linked to slower theta oscillations in the hippocampus (Allen et al., 2011), as well as reduced power in gamma frequency and theta-phase modulation of gamma (Buhl et al., 2003). Interestingly, recent in vivo data showed that distinct interneuron subtypes mediate oscillations in theta and gamma frequency bands (Fukunaga et al., 2014). Together with these studies, our findings support the idea that electrical coupling in interneuron populations is important for orchestrating the temporal structure of network activity.

Functional Role of Precisely Correlated Activity in the Cerebellar Circuit

The subthreshold membrane potential correlations combined with spike-to-spike transmission between Golgi cells ensure strong and precisely correlated activity in the face of spontaneous synaptic background activity. Furthermore, strong sensory-evoked bursts of synaptic inputs that drive spiking in individual cells enhance this precisely correlated activity. This correlated activity effectively serves to distribute MF and/or parallel fiber inputs throughout the coupled Golgi cell network, resulting in enhanced inhibition of downstream granule cells. It is known that tonic inhibition strongly determines the excitability and sensory responses of granule cells (Chadderton et al., 2004; Duguid et al., 2012). Temporally precise phasic inhibition, however, likely controls the spike timing of granule cells, and thus may control the occurrence of oscillatory or other synchronized patterns of granule cell activity (Dugué et al., 2009; Simões de Souza and De Schutter, 2011). As Golgi cells are coupled in local networks, the correlated activity of each unit in an ensemble would summate and strongly inhibit postsynaptic granule cells that were preferentially contacted by coupled Golgi cells. It will therefore be crucial to determine the exact spatial anatomical divergence and convergence of Golgi cell axons to granule cell dendrites and the location of the granule cells that are contacted by coupled ensembles of Golgi cells.

Relevance of Golgi Cell-Correlated Activity to Motor Control

The ability to generate correlated inhibitory activity suggests that precisely timed feedforward inhibition (D'Angelo and De Zeeuw, 2009; Kanichay and Silver, 2008) and ''time-windowing'' of activity (D'Angelo, 2008; D'Angelo and De Zeeuw, 2009) play an important role in cerebellar computations. The exact relevance of precisely correlated activity between neighboring Golgi cells for cerebellar sensorimotor behavior remains to be determined, but some clues may be found in the phenotype of Cx36-KO mice, which have been shown to display impaired timing of locomotion, conditioned eye-blink responses, and motor learning (Frisch et al., 2005; Van Der Giessen et al., 2008; Zlomuzica et al., 2012). However, as Cx36 is also expressed in other cell types in the cerebellar circuit, including molecular layer interneurons, as well as in the inferior olive, which provides input to the cerebellum, a general Cx36-KO is not a good model to identify the behavioral relevance of Golgi cell-correlated activity. Nevertheless, specific ablation of Golgi cells in a transgenic mouse line expressing human interleukin-2 receptor α subunit using an immunotoxin-mediated cell targeting technique resulted in severe acute ataxia and a chronic inability to perform compound movements (Watanabe et al., 1998), highlighting the importance of this cell type in regulating motor control.

Conclusion

Our data provide a mechanistic understanding of how electrical coupling causes correlated spikes in electrically coupled interneurons with millisecond precision in the context of the spontaneous and sensory-evoked synaptic drive in vivo. Importantly, the mechanism we describe should be relevant to most electrically coupled neurons in the brain, as it is generic and independent of spike shape. This suggests that electrically coupled interneurons will spike together with millisecond precision irrespective of the exact temporal structure of common synaptic input. Our results provide further support to the idea that electrical coupling is crucial for temporal computations performed in mammalian neural circuits.

EXPERIMENTAL PROCEDURES

Please see Supplemental Experimental Procedures for the full experimental procedures. Briefly, all animal procedures were performed under license from the UK Home Office in accordance with the Animal (Scientific Procedures) Act 1986. Male and female transgenic wild-type and connexin36 KO mice (P20-45) expressing EGFP under the GlyT2 promotor were used to identify and target Golgi cells. In vivo targeted patch-clamp recordings (Margrie et al., 2003) were performed using a custom two-photon microscope (MOM, Sutter) to visualize EGFP positive Golgi cells in Crus II under ketamine/xylazine anesthesia. Sensory stimulation was performed used an airpuff (100 ms, 30–40 psi) delivered to the perioral region and/or whisker pad.

Simulations were performed using NEURON 7.1 (Hines and Carnevale, 1997). Two reduced compartmental neuron models of Golgi cells, each consisting of a soma and a dendrite, were coupled via a gap junction (resistance, 3 GΩ) at a proximal dendritic location (Vervaeke et al., 2010). The gap junction was operated in different modes (Figure 6) in which it was switched on or off selectively during subthreshold signaling, the DJP, and/or the HJP. Ongoing synaptic inputs in vivo were simulated by Poisson spike trains triggering synaptic conductances that were distributed uniformly over the soma and dendrites of both neurons.

SUPPLEMENTAL INFORMATION

Supplemental Information includes seven figures and Supplemental Experimental Procedures and can be found with this article at http://dx.doi.org/10.1016/j.neuron.2016.04.013.

AUTHOR CONTRIBUTIONS

I.v.W. performed all experiments and analyses with the exception of the single whole-cell recordings of sensory-evoked responses and their analyses, which were performed by S.S.H. and S.K. The simulations were performed by A.R. The study was designed by I.v.W. and M.H., who also wrote the paper.

ACKNOWLEDGMENTS

We are grateful to Michael London, Alex Mathy, Kazuo Kitamura, Martha Havenith, and Christoph Schmidt-Hieber for advice on experiments and analysis, and to Mark Farrant, Charlotte Arlt, Michael London, and Christoph Schmidt-Hieber for discussion and comments on the manuscript. We thank Arifa Naeem for help with immunocytochemistry and Arifa Naeem, Salpie Nowinski, and Stuart Martin for help with genotyping. This work was supported by grants from the Human Frontier Science Program Organization (I.v.W.), the Gatsby Charitable Foundation (M.H.), the European Research Council (M.H.), The Wellcome Trust (M.H.), and the European Commission (FP7; Eurospin, M.H. and I.v.W.).

Received: August 12, 2015
Revised: February 18, 2016
Accepted: April 1, 2016
Published: May 5, 2016

REFERENCES

Allen, K., Fuchs, E.C., Jaschonek, H., Bannerman, D.M., and Monyer, H. (2011). Gap junctions between interneurons are required for normal spatial coding in the hippocampus and short-term spatial memory. J. Neurosci. *31*, 6542–6552.

Bell, C.C., and Kawasaki, T. (1972). Relations among climbing fiber responses of nearby Purkinje Cells. J. Neurophysiol. *35*, 155–169.

Buhl, D.L., Harris, K.D., Hormuzdi, S.G., Monyer, H., and Buzsáki, G. (2003). Selective impairment of hippocampal gamma oscillations in connexin-36 knock-out mouse in vivo. J. Neurosci. *23*, 1013–1018.

Chadderton, P., Margrie, T.W., and Häusser, M. (2004). Integration of quanta in cerebellar granule cells during sensory processing. Nature *428*, 856–860.

Chow, C.C., and Kopell, N. (2000). Dynamics of spiking neurons with electrical coupling. Neural Comput. *12*, 1643–1678.

Connors, B.W., and Long, M.A. (2004). Electrical synapses in the mammalian brain. Annu. Rev. Neurosci. *27*, 393–418.

Curti, S., and Pereda, A.E. (2004). Voltage-dependent enhancement of electrical coupling by a subthreshold sodium current. J. Neurosci. *24*, 3999–4010.

D'Angelo, E. (2008). The critical role of Golgi cells in regulating spatio-temporal integration and plasticity at the cerebellum input stage. Front. Neurosci. *2*, 35–46.

D'Angelo, E., and De Zeeuw, C.I. (2009). Timing and plasticity in the cerebellum: focus on the granular layer. Trends Neurosci. *32*, 30–40.

Diba, K., Amarasingham, A., Mizuseki, K., and Buzsáki, G. (2014). Millisecond timescale synchrony among hippocampal neurons. J. Neurosci *34*, 14984–14994.

Dugué, G.P., Brunel, N., Hakim, V., Schwartz, E., Chat, M., Lévesque, M., Courtemanche, R., Léna, C., and Dieudonné, S. (2009). Electrical coupling mediates tunable low-frequency oscillations and resonance in the cerebellar Golgi cell network. Neuron *61*, 126–139.

Duguid, I., Branco, T., London, M., Chadderton, P., and Häusser, M. (2012). Tonic inhibition enhances fidelity of sensory information transmission in the cerebellar cortex. J. Neurosci. *32*, 11132–11143.

Frisch, C., De Souza-Silva, M.A., Söhl, G., Güldenagel, M., Willecke, K., Huston, J.P., and Dere, E. (2005). Stimulus complexity dependent memory impairment and changes in motor performance after deletion of the neuronal gap junction protein connexin36 in mice. Behav. Brain Res. *157*, 177–185.

Fukunaga, I., Herb, J.T., Kollo, M., Boyden, E.S., and Schaefer, A.T. (2014). Independent control of gamma and theta activity by distinct interneuron networks in the olfactory bulb. Nat. Neurosci. *17*, 1208–1216.

Galarreta, M., and Hestrin, S. (1999). A network of fast-spiking cells in the neocortex connected by electrical synapses. Nature *402*, 72–75.

Galarreta, M., and Hestrin, S. (2001). Electrical synapses between GABA-releasing interneurons. Nat. Rev. Neurosci. *2*, 425–433.

Gentet, L.J., Avermann, M., Matyas, F., Staiger, J.F., and Petersen, C.C. (2010). Membrane potential dynamics of GABAergic neurons in the barrel cortex of behaving mice. Neuron *65*, 422–435.

Gibson, J.R., Beierlein, M., and Connors, B.W. (1999). Two networks of electrically coupled inhibitory neurons in neocortex. Nature *402*, 75–79.

Heck, D.H., Thach, W.T., and Keating, J.G. (2007). On-beam synchrony in the cerebellum as the mechanism for the timing and coordination of movement. Proc. Natl. Acad. Sci. USA *104*, 7658–7663.

Heck, D.H., De Zeeuw, C.I., Jaeger, D., Khodakhah, K., and Person, A.L. (2013). The neuronal code(s) of the cerebellum. J. Neurosci. *33*, 17603–17609.

Hines, M.L., and Carnevale, N.T. (1997). The NEURON simulation environment. Neural Comput. *9*, 1179–1209.

Holtzman, T., Mostofi, A., Phuah, C.L., and Edgley, S.A. (2006a). Cerebellar Golgi cells in the rat receive multimodal convergent peripheral inputs via the lateral funiculus of the spinal cord. J. Physiol. *577*, 69–80.

Holtzman, T., Rajapaksa, T., Mostofi, A., and Edgley, S.A. (2006b). Different responses of rat cerebellar Purkinje cells and Golgi cells evoked by widespread convergent sensory inputs. J. Physiol. *574*, 491–507.

Hu, H., Martina, M., and Jonas, P. (2010). Dendritic mechanisms underlying rapid synaptic activation of fast-spiking hippocampal interneurons. Science *327*, 52–58.

Hull, C., and Regehr, W.G. (2012). Identification of an inhibitory circuit that regulates cerebellar Golgi cell activity. Neuron *73*, 149–158.

Kanichay, R.T., and Silver, R.A. (2008). Synaptic and cellular properties of the feedforward inhibitory circuit within the input layer of the cerebellar cortex. J. Neurosci. *28*, 8955–8967.

Kvitsiani, D., Ranade, S., Hangya, B., Taniguchi, H., Huang, J.Z., and Kepecs, A. (2013). Distinct behavioural and network correlates of two interneuron types in prefrontal cortex. Nature *498*, 363–366.

Lampl, I., Reichova, I., and Ferster, D. (1999). Synchronous membrane potential fluctuations in neurons of the cat visual cortex. Neuron *22*, 361–374.

Landisman, C.E., Long, M.A., Beierlein, M., Deans, M.R., Paul, D.L., and Connors, B.W. (2002). Electrical synapses in the thalamic reticular nucleus. J. Neurosci. *22*, 1002–1009.

Long, M.A., Jutras, M.J., Connors, B.W., and Burwell, R.D. (2005). Electrical synapses coordinate activity in the suprachiasmatic nucleus. Nat. Neurosci. *8*, 61–66.

Mann-Metzer, P., and Yarom, Y. (1999). Electrotonic coupling interacts with intrinsic properties to generate synchronized activity in cerebellar networks of inhibitory interneurons. J. Neurosci. *19*, 3298–3306.

Marr, D. (1969). A theory of cerebellar cortex. J. Physiol. *202*, 437–470.

Mitchell, S.J., and Silver, R.A. (2003). Shunting inhibition modulates neuronal gain during synaptic excitation. Neuron *38*, 433–445.

Ostojic, S., Brunel, N., and Hakim, V. (2009). Synchronization properties of networks of electrically coupled neurons in the presence of noise and heterogeneities. J. Comput. Neurosci. *26*, 369–392.

Person, A.L., and Raman, I.M. (2012a). Purkinje neuron synchrony elicits time-locked spiking in the cerebellar nuclei. Nature *481*, 502–505.

Person, A.L., and Raman, I.M. (2012b). Synchrony and neural coding in cerebellar circuits. Front. Neural Circuits *6*, 97.

Pfeuty, B., Mato, G., Golomb, D., and Hansel, D. (2003). Electrical synapses and synchrony: the role of intrinsic currents. J. Neurosci. *23*, 6280–6294.

Poulet, J.F., and Petersen, C.C. (2008). Internal brain state regulates membrane potential synchrony in barrel cortex of behaving mice. Nature *454*, 881–885.

Powell, K., Mathy, A., Duguid, I., and Häusser, M. (2015). Synaptic representation of locomotion in single cerebellar granule cells. eLife *4*, 4, http://dx.doi.org/10.7554/eLife.07290.

Prsa, M., Dash, S., Catz, N., Dicke, P.W., and Thier, P. (2009). Characteristics of responses of Golgi cells and mossy fibers to eye saccades and saccadic adaptation recorded from the posterior vermis of the cerebellum. J. Neurosci. *29*, 250–262.

Rudolph, S., Hull, C., and Regehr, W.G. (2015). Active dendrites and differential distribution of calcium channels enable functional compartmentalization of Golgi cells. J. Neurosci. *35*, 15492–15504.

Sasaki, K., Bower, J.M., and Llinás, R. (1989). Multiple Purkinje Cell Recording in Rodent Cerebellar Cortex. Eur. J. Neurosci. *1*, 572–586.

Simões de Souza, F.M., and De Schutter, E. (2011). Robustness effect of gap junctions between Golgi cells on cerebellar cortex oscillations. Neural Syst. Circuits *1*, 7.

Swadlow, H.A., Beloozerova, I.N., and Sirota, M.G. (1998). Sharp, local synchrony among putative feed-forward inhibitory interneurons of rabbit somatosensory cortex. J. Neurophysiol. *79*, 567–582.

Tahon, K., Volny-Luraghi, A., and De Schutter, E. (2005). Temporal characteristics of tactile stimuli influence the response profile of cerebellar Golgi cells. Neurosci. Lett. *390*, 156–161.

Tahon, K., Wijnants, M., De Schutter, E., and Maex, R. (2011). Current source density correlates of cerebellar Golgi and Purkinje cell responses to tactile input. J. Neurophysiol. *105*, 1327–1341.

Trenholm, S., McLaughlin, A.J., Schwab, D.J., Turner, M.H., Smith, R.G., Rieke, F., and Awatramani, G.B. (2014). Nonlinear dendritic integration of electrical and chemical synaptic inputs drives fine-scale correlations. Nat. Neurosci. *17*, 1759–1766.

Van Der Giessen, R.S., Koekkoek, S.K., van Dorp, S., De Gruijl, J.R., Cupido, A., Khosrovani, S., Dortland, B., Wellershaus, K., Degen, J., Deuchars, J., et al. (2008). Role of olivary electrical coupling in cerebellar motor learning. Neuron *58*, 599–612.

Vervaeke, K., Lorincz, A., Gleeson, P., Farinella, M., Nusser, Z., and Silver, R.A. (2010). Rapid desynchronization of an electrically coupled interneuron network with sparse excitatory synaptic input. Neuron *67*, 435–451.

Vervaeke, K., Lorincz, A., Nusser, Z., and Silver, R.A. (2012). Gap junctions compensate for sublinear dendritic integration in an inhibitory network. Science *335*, 1624–1628.

Volny-Luraghi, A., Maex, R., Vosdagger, B., and De Schutter, E. (2002). Peripheral stimuli excite coronal beams of Golgi cells in rat cerebellar cortex. Neuroscience *113*, 363–373.

Vos, B.P., Maex, R., Volny-Luraghi, A., and De Schutter, E. (1999a). Parallel fibers synchronize spontaneous activity in cerebellar Golgi cells. J. Neurosci. *19*, RC6.

Vos, B.P., Volny-Luraghi, A., and De Schutter, E. (1999b). Cerebellar Golgi cells in the rat: receptive fields and timing of responses to facial stimulation. Eur. J. Neurosci. *11*, 2621–2634.

Watanabe, D., Inokawa, H., Hashimoto, K., Suzuki, N., Kano, M., Shigemoto, R., Hirano, T., Toyama, K., Kaneko, S., Yokoi, M., et al. (1998). Ablation of cerebellar Golgi cells disrupts synaptic integration involving GABA inhibition and NMDA receptor activation in motor coordination. Cell *95*, 17–27.

Zlomuzica, A., Viggiano, D., Degen, J., Binder, S., Ruocco, L.A., Sadile, A.G., Willecke, K., Huston, J.P., and Dere, E. (2012). Behavioral alterations and changes in Ca/calmodulin kinase II levels in the striatum of connexin36 deficient mice. Behav. Brain Res. *226*, 293–300.

Neuron, Volume *90*

Supplemental Information

Conditional Spike Transmission Mediated by Electrical Coupling Ensures Millisecond Precision-Correlated Activity among Interneurons In Vivo

Ingrid van Welie, Arnd Roth, Sara S.N. Ho, Shoji Komai, and Michael Häusser

Supplemental Figures

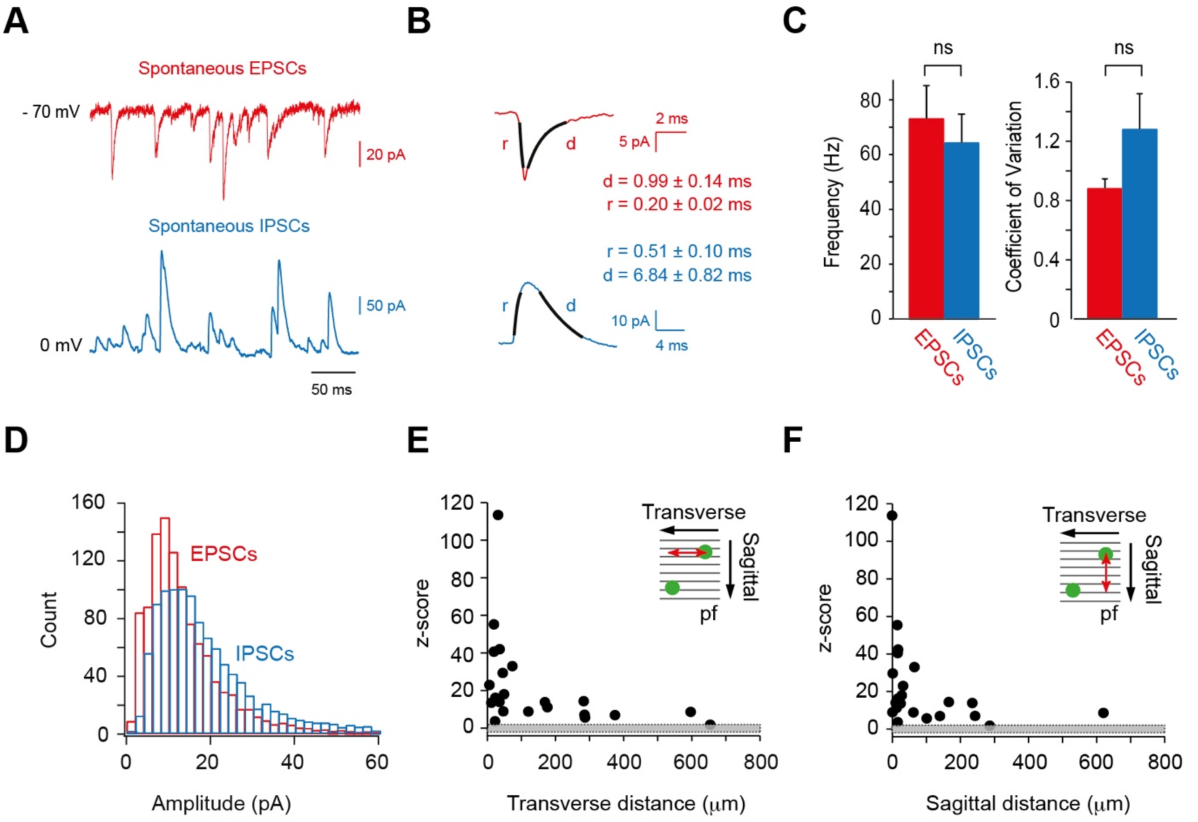

Figure S1 (related to Figure 1). Background synaptic activity in Golgi cells and distance-dependence of coupling in transverse and sagittal planes

(A) Using a cesium-based internal, spontaneous EPSCs were recorded at -70 mV and spontaneous IPSCs were recorded at 0 mV. (B) Mean EPSC and IPSC waveforms from the example recordings shown in (A). Mean waveforms were fitted with single exponential functions to obtain kinetic values for EPSC rise and decay times. The mean values across cells for rise (r) and decay (d) times are indicated next to the EPSC and IPSC waveforms (n = 6 neurons). (C) Summary bar graph of mean ± s.e.m. of spontaneous EPSC and IPSC frequencies and the coefficient of variation of event times for both (n = 6 neurons). (D) Mean amplitude distribution of all identified EPSCs and IPSCs across cells (n = 6 neurons). (E) Degree of correlated activity found in Golgi cell pairs at various inter-neuronal distances along the transverse axis (inset displays field of view from the top of the brain and how transverse distance was measured between cell somata; pf = parallel fibers). (F) Degree of correlated activity found in Golgi cell pairs at various inter-neuronal distances along the sagittal axis. Grey areas in (E) and (F) indicate confidence interval (z-scores between -2 to +2).

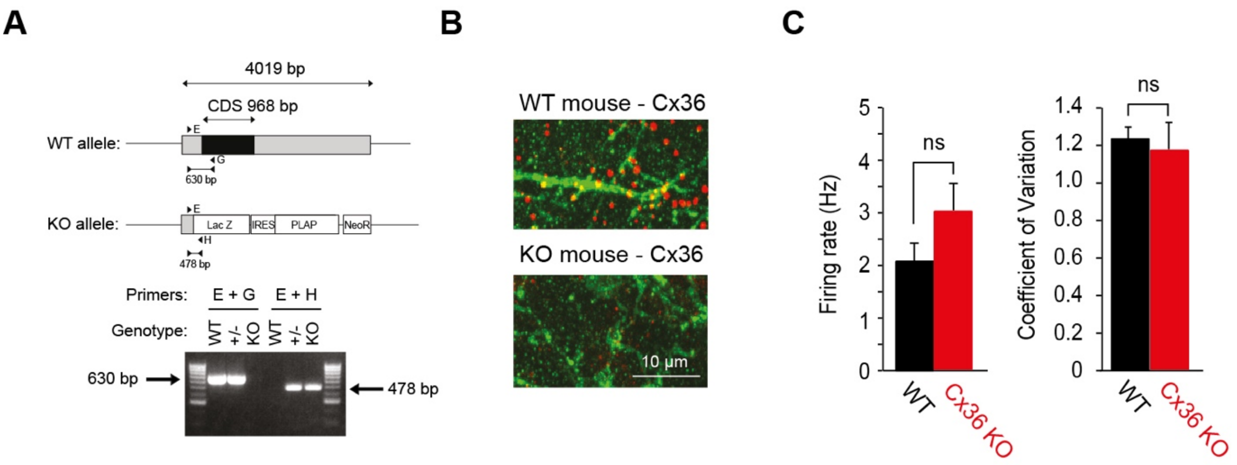

Figure S2 (related to Figure 2). Characterization of GlyT2-EGFP/Cx36 KO mice

(A) A new mouse line was created by crossing the GlyT2-EGFP mice with Cx36-KO mice. To confirm that the wildtype (WT) Cx36 gene sequence was still disrupted in the new mouse line, we used a unique set of primers (E, G & H) to identify both the WT and KO allele (see Supplemental Experimental Procedures). On the bottom an example PCR result summarizing results for the identification of WT, heterozygous and homozygous KO mice. Only homozygous mice were used for experiments. (B) To confirm the genotype of mice, we performed immunolabelling for the Cx36 protein in WT and KO mice. In WT mice, numerous Cx36 puncta are present in the molecular layer of the cerebellum many of which co-localize with Golgi cell dendrites, while these are largely absent in the KO mice. (C) Summary bar graph comparing the mean firing rates and the coefficient of variation of interspike intervals of Golgi cells in WT and Cx36-KO mice. Neither parameter was significantly different ($p = 0.06$ and $p = 0.13$ respectively).

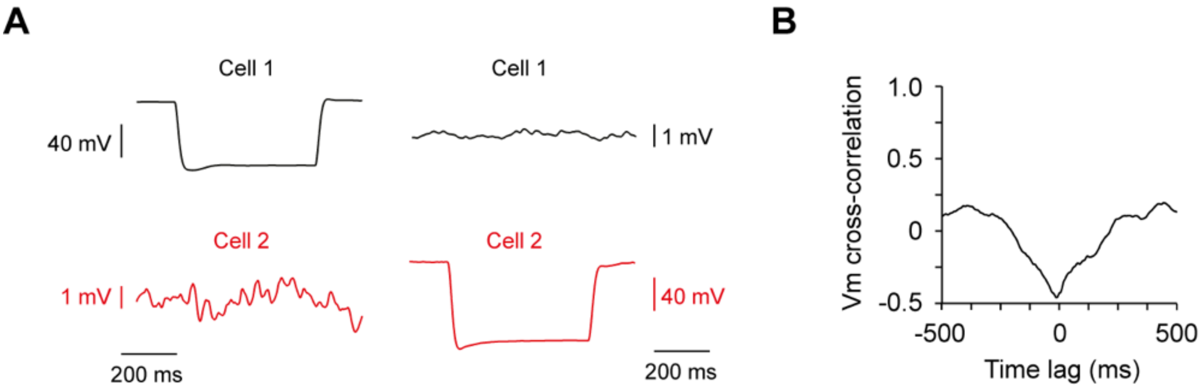

Figure S3 (related to Figure 3). Dual whole-cell recording from a non-coupled Golgi cell pair

(A) Simultaneous dual whole-cell recording from two Golgi cells that were located nearby (intersomatic distance = 24 μm), but were not electrically coupled: injecting hyperpolarizing current into one cell produced no change in membrane potential of the other cell. (B) There was no positive, but instead a negative cross-correlation of membrane potentials in these two non-coupled Golgi cells.

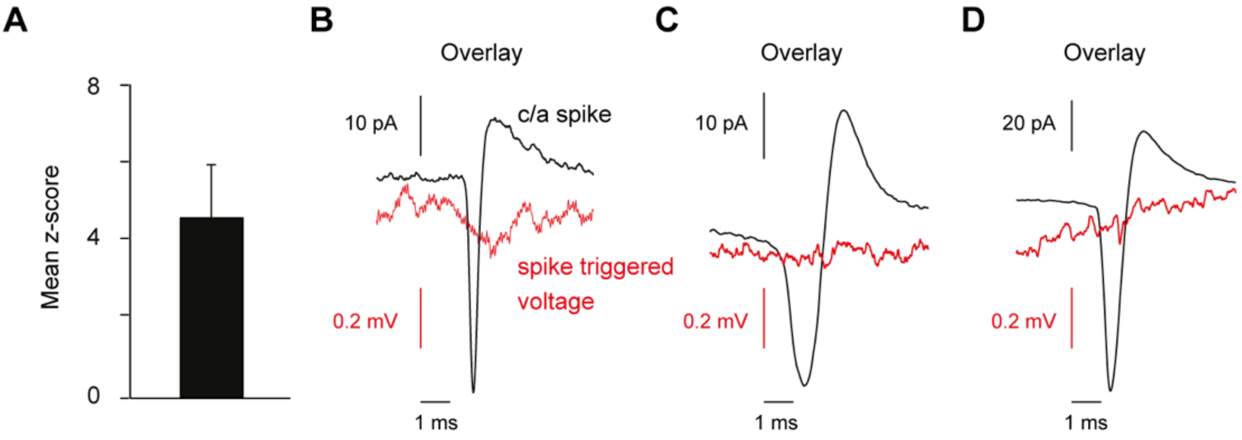

Figure S4 (related to Figure 4). In pairs with low precisely correlated activity, spikelets are absent

(A) Mean z-score (5 ± 1, n = 3) from pairs in which no to low precisely correlated activity was found. (B-C) Overlay of the mean cell-attached (c/a) spike (red trace) and the corresponding spike-triggered voltage (black trace) in the w/c recording in the 3 pairs in which there was no to low degrees of precisely correlated spiking (see also Figure S5). In all three examples, there were no spike-triggered depolarizing membrane potential changes beyond noise fluctuations (mean depolarization = 0.05 ± 0.01 mV in first 1.5 ms following c/a spike, n = 3), suggesting that these pairs were not electrically coupled.

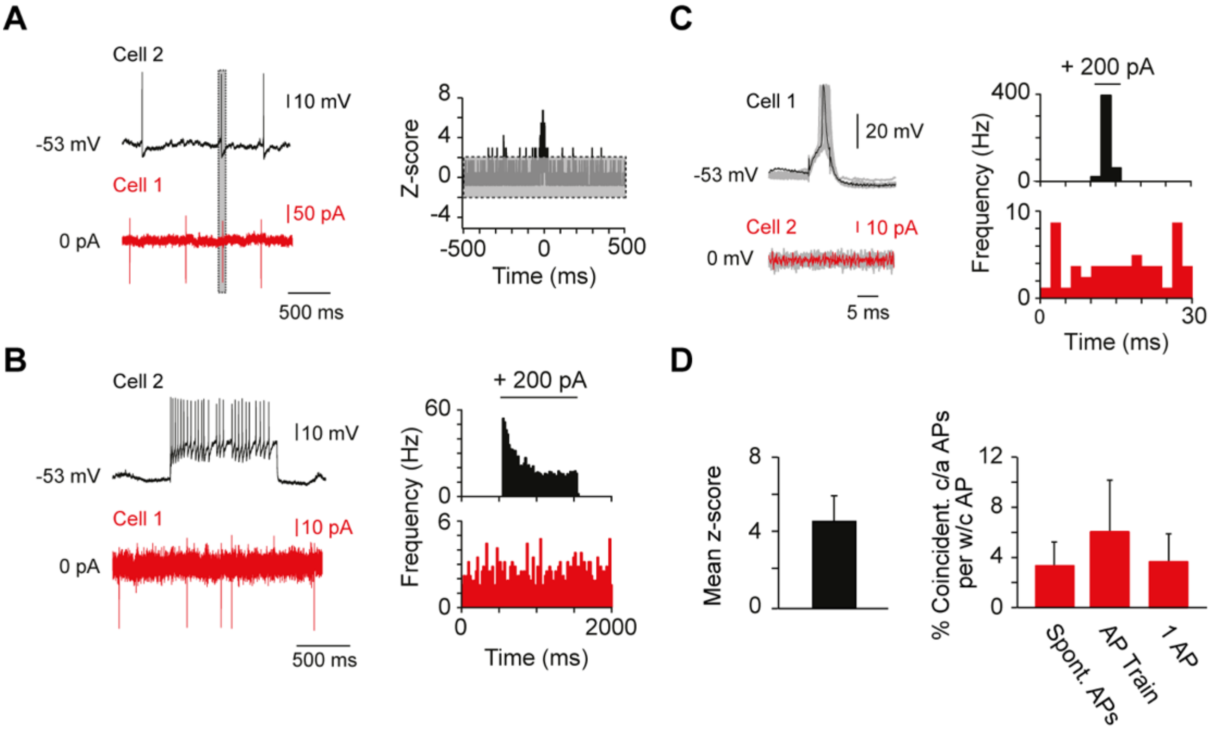

Figure S5 (related to Figure 5). In pairs with low precisely correlated activity, induced spikes in one cell do not trigger spikes in the paired cell

(A) Example traces (left) and spike time cross-correlogram (right) of a dual whole-cell (w/c) and cell-attached (c/a) recording (same experimental configuration as in Figures 4 and 5), in which the spontaneous spiking of the pair was only mildly correlated (max z-score = 7, cross-correlogram left). Grey area on trace indicates the occurrence of correlated spikes (< ± 5 ms time lag). Grey area on cross-correlogram indicates confidence interval (z-scores between -2 to +2). (B) In the same pair as in (A), inducing a train of action potentials in Cell 2 failed to induce correlated action potentials in Cell 1 as is evident from the PSTH (right, 20 ms bins) summarizing the results across multiple trials. (C) Similarly, inducing single action potentials in Cell 2 failed to induce any correlated spikes in Cell 1 across trials (PSTH 2 ms bins). (D) Summary data across 3 such non- to mildly correlated pairs in which spike injection experiments failed to induce correlated spikes. The mean z-score across these pairs was 5 ± 1 (left) and the mean percentages of correlated spikes associated with each w/c spike were very low across the different conditions (3 ± 2%, 6 ± 4% and 4 ± 2%, respectively).

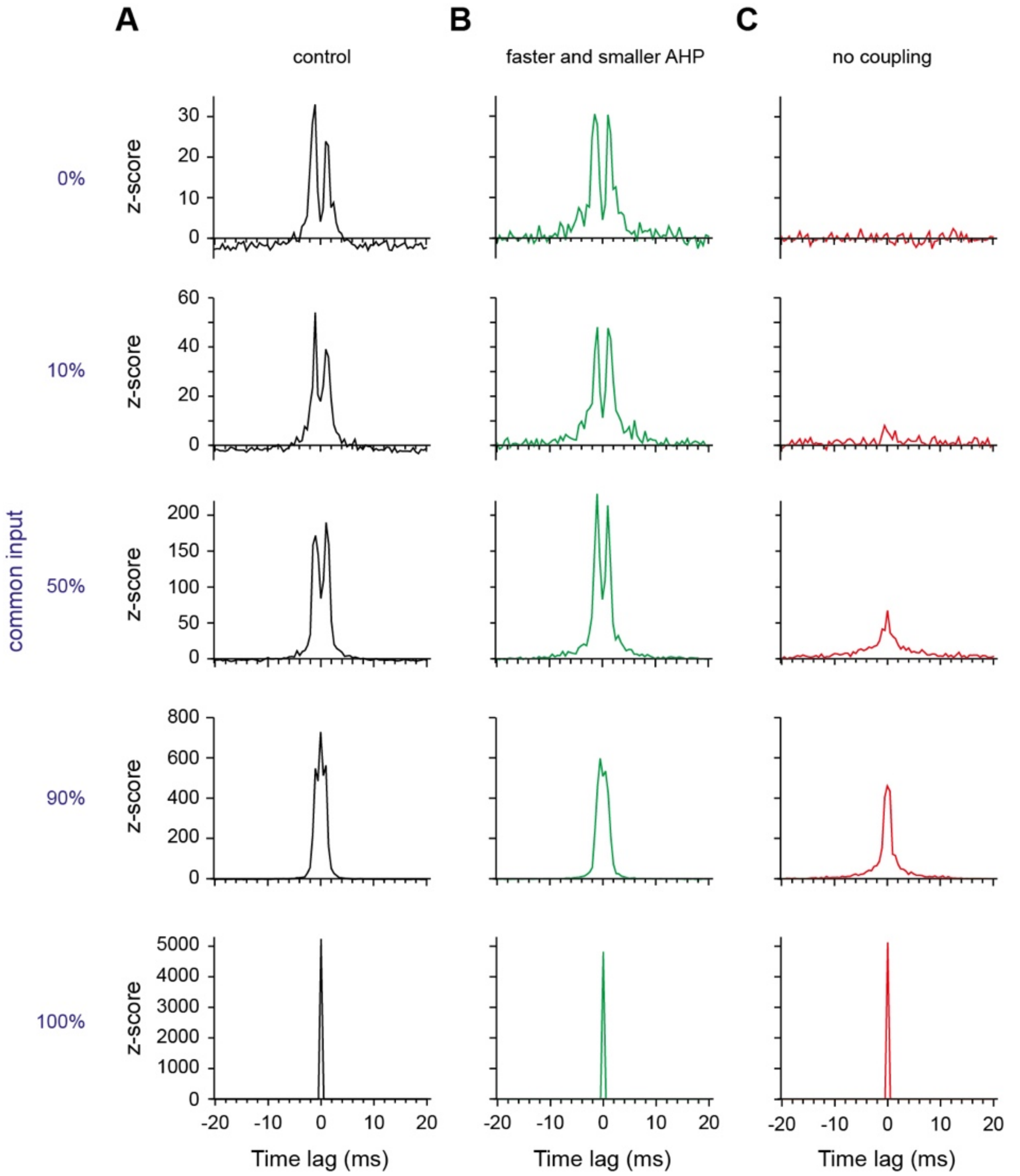

Figure S6 (related to Figure 6). Effect of degree of precisely synchronous common input and spike shape on degree of correlated activity

(A) Cross-correlograms of spike timing of two coupled Golgi cells in a computational model as a function of increasing degrees of precisely synchronous common input. Under control conditions, the somatic spike shape has a long and deep AHP (amplitude, 17 mV; duration, 14 ms FWHM). (B) Same data as in a, but using a somatic spike with a faster and smaller AHP (amplitude, 8 mV; duration, 9 ms FWHM). (C) Cross-correlograms as a function of increasing degrees of precisely synchronous common input in the absence of electrical coupling.

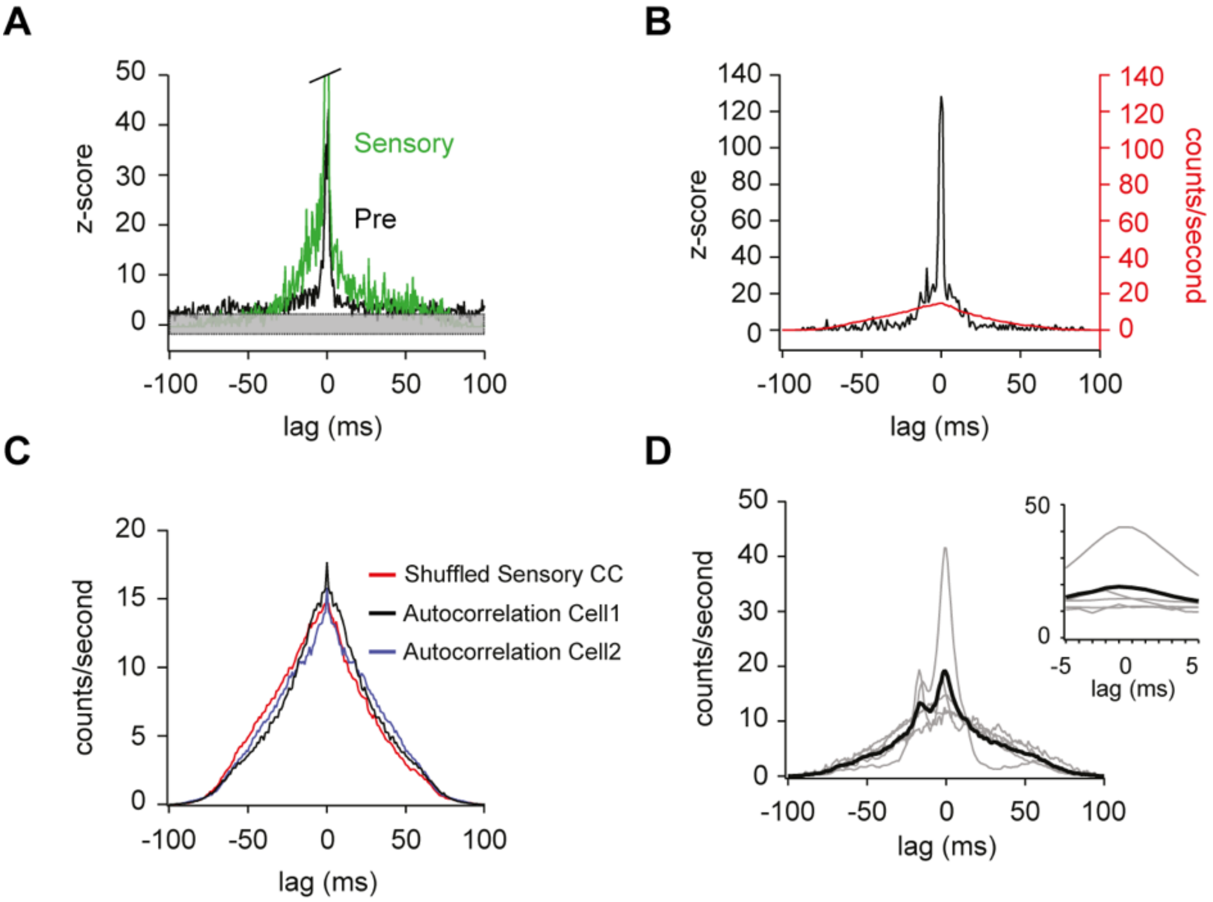

Figure S7 (related to Figure 8). Variation in temporal profile of sensory-evoked responses in individual cells and pairs

(A) Mean cross-correlograms (n = 5) computed from baseline spontaneous spiking (Pre, black trace) and during sensory stimuli (Sensory, green trace). Correlations at slower time lags are enhanced during sensory stimuli. The peak of the sensory evoked correlation is clipped for display purposes. Grey area indicates confidence interval (z-scores between -2 to +2). (B) Cross-correlogram (black trace) of sensory-evoked spiking from the same pair of Golgi cells as shown in Figure 8B and 8C. Shuffling the spike times of the sensory evoked spikes across trials in one cell of the pair reveals a slow temporal correlation (red trace) that resembles the base or "foot" of the real cross-correlogram. (C) The shuffled sensory-evoked cross-correlogram (red trace) closely resembles the autocorrelations of the spike times in each cell of the pair (black and blue traces), suggesting that the variation in spike timing in individual Golgi cells leads to the broad temporal correlation reflected in the base of the cross-correlogram in sensory evoked spiking in a Golgi cell pair as shown in (A). (D) Individual (grey traces) and mean (black trace) shuffled sensory-evoked cross-correlograms indicating broad temporal correlations in sensory evoked spiking in all pairs (n = 5). Inset shows same figure as (D), but only showing time lags from -5 to 5 ms, to directly compare with Figure 8G. Compared to Figure 8G, there is no sharp peak around 0 ms.

Supplemental Experimental Procedures

Transgenic animals and surgical preparation

Animal procedures were performed under license from the UK Home Office in accordance with the Animal (Scientific Procedures) Act 1986. Male and female transgenic mice expressing EGFP under the GlyT2 promotor (Zeilhofer et al., 2005) were backcrossed into C57Bl6 mice for multiple generations (F5-F19) and were used to identify and target Golgi cells (Simat et al., 2007). Mice (P20-45) were anaesthetized by intraperitoneal injection of ketamine (80-100 mg kg^{-1}) / xylazine (5-10 mg kg^{-1}). Body temperature was monitored using a rectal probe and maintained at 37°C using a homeothermic blanket. Craniotomies (1 mm^2) were made over Crus II. The dura was removed and the brain was covered using 1.5% agarose in HEPES buffered artificial cerebral spinal fluid (150 mM NaCl, 2.5 mM KCl, 10 mM HEPES, 2 mM $CaCl_2$ and 1 mM $MgCl_2$) to reduce breathing related movement. Connexin36 knockout (Cx36-KO) mice (Deans et al., 2001) were crossed with heterozygous GlyT2-EGFP mice and bred further to generate mice homozygous for the Cx36 gene and heterozygous for EGFP driven under the GlyT2 promoter. To confirm that the wildtype (WT) Cx36 gene sequence had been disrupted, we used a new set of primers to amplify through the start codon region of the Cx36 gene. We designed a common forward PCR primer, E (5' CTC AGA CCG CAA GAT CGC G 3') to the upstream non-coding region of the Cx36 gene (Gene ID 320846), and a 5' primer, G (5' CAC TGG GCC TCG GGT AAT 3') in the WT downstream coding sequence. The common forward primer was used in combination with either primer G, or a Lac Z reverse primer, H (5' GCC TCT TCG CTA ATT ACG 3'), corresponding to the bicistronic cassette which was used to disrupt Cx36, to amplify the target alleles. Routine genotyping of Cx36-KO/GlyT2-EGFP mice was performed using primers targeting the neo cassette in the transgenic genome.

In vivo single and dual patch-clamp recordings

In vivo targeted patch-clamp recordings (Margrie et al., 2003) were performed using a custom two-photon microscope (MOM, Sutter) to visualize EGFP positive cells in the cerebellar granule cell layer. The pipette solution used for cell-attached and whole-cell recordings contained (in mM): 125 K-Methanesulphonate, 7 KCl, 10 HEPES, 2 Mg-ATP, 2 Na_2ATP, 0.5 Na_2GTP, 0.05 EGTA and 50 µM Alexa Fluor 594 (pH 7.3 with KOH, 280-290 mOsm). For recordings of spontaneous EPSCs and IPSCs within single cells, a Cs-based internal was used containing (in mM): 120 Cs-Methanesulphonate, 10 HEPES, 2 Mg-ATP, 2 Na_2-ATP, 0.5 Na_2GTP, 5 EGTA, 5 QX-314, 10 TEA-Cl and 50 µM Alexa Fluor 594 (pH 7.3 with CsOH, 280-290 mOsm). Recordings were made using a Multiclamp 700B amplifier (Molecular Devices) and a Digidata acquisition system (Molecular Devices). Data were filtered at 3-10 kHz and acquired at 20 or 40 kHz using pClamp 9.0 software (Molecular Devices). Patch pipettes were pulled using a vertical puller (Narishige) from thick walled borosilicate glass and had a resistance of 5-7 MΩ. Access resistance for whole-cell recordings was between 20-70 MΩ. Membrane potentials were not corrected for liquid junction

potentials. For the purpose of analysis of baseline correlated activity and for spike induction protocols, only pairs in which both cells were spiking and in which extracellular spikes could be easily distinguished from noise by eye were included for analysis. Sensory stimulation was performed by delivering a 100 ms airpuff (30-40 psi) to the perioral region and/or whisker pad using a pneumatic pressure device (PDES-02DX, NPI electronic GmbH). There was an experimentally determined 15 ms delay before the pressurized air reached the mouse. Only pairs with a latency in the sensory response < 50 ms following this 15 ms delay were used for analysis.

Immunocytochemistry

For immunodetection of Cx-36, WT GlyT2-EGFP and Cx36-KO/GlyT2-EGFP mice were perfused with 4% paraformaldehyde (PF). The protocol used for immunodetection of Cx36 was as previously published (Vervaeke et al., 2012). In this protocol, tissue was not post-fixed, and this led to loss of EGFP signal in our tissue. As a consequence, an antibody against mGluR2 (which labels Golgi cells specifically) was used to label Golgi cell dendrites. In short, for co-detection of both Cx36 and mGluR2, slices (60 μm) were cut and washed in PBS, and then incubated in primary antibody (mouse monoclonal anti-Cx36, MAB3045, Chemicon and rabbit polyclonal anti-mGluR2/3, 06-676, Millipore, Billerica, MA) overnight at room temperature. After washing in PBS, slices were incubated in secondary antibody for 2 hours at room temperature. Slices were then washed again with PBS and mounted using Vectashield mounting medium. A confocal microscope system (Perkin-Elmer spinning disk) was used to take images of labeled tissue.

Event detection and analysis

Spike and synaptic event detection was performed off-line using custom written software in Igor Pro (Tarotools, courtesy of Taro Ishikawa). Events were detected using a threshold and subsequently individually verified manually. As a complementary method, a deconvolution-based algorithm (Pernia-Andrade et al., 2012) implemented in Stimfit (courtesy of Christoph Schmidt-Hieber) was used to detect synaptic events based on an average kinetic template of individual synaptic events. After low-pass filtering of the deconvolution result at 0.2 and using a detection threshold (θ) of 4 (4 times the standard deviation of the baseline Gaussian noise), this method gave results similar to that of manual verification. Cross-correlograms of spike times or synaptic event times were computed using custom-written code in Matlab. Cross-correlograms were normalized by creating 30 shuffled spike trains for each recording and subtracting the mean shuffled cross-correlogram from the raw mean cross-correlogram and by dividing by the standard deviation of the mean shuffled cross-correlogram. Z-scores between ± 2 were taken as the confidence interval, outside of which values were considered significant. Z-scores were computed from cross-correlograms using 0.5 ms bins. Spike coincidence percentages were standardly computed from cross-correlograms using 1 ms bins unless noted otherwise. The EPSC rate cross-correlation was computed by binning synaptic event

times, subtracting the mean from the resulting input rate functions, and computing the normalized cross-correlation of the mean-subtracted input rates so that the autocorrelations at zero lag equal 1. For cross-correlograms of membrane voltage or current, Matlab's built-in xcorr function was used and spectral analysis was performed using Matlab's built-in mscohere function. Spike-triggered averages of spikelet voltages and currents were determined in Igor Pro using Neuromatic (courtesy of Jason Rothman). Statistics were performed using Instat 3 (Graphpad). Two-tailed non-parametric Mann-Whitney tests were used for comparing unpaired data and two-tailed repeated measures Anova using standard parametric measures & Tukey post comparison testing or a two tailed Student's T-test were used for comparing paired data. Data is reported as mean ± standard error of the mean (s.e.m.).

Model

Simulations were performed using NEURON 7.1 (Hines and Carnevale, 1997). Two reduced compartmental neuron models, each consisting of a soma and a dendrite, were coupled via a gap junction (resistance, 3 GΩ) at a proximal dendritic location corresponding to 25% of the length of each dendrite (Vervaeke et al., 2010). Both the soma (length = 30 μm; diameter = 30 μm) and dendrite (length = 200 μm, subdivided into 50 compartments; diameter = 1 μm) of each neuron had homogeneous passive membrane properties (specific membrane capacitance C_m = 1 μF/cm^2; specific membrane resistance R_m = 20000 Ω cm^2). The passive reversal potential was set to E = –46 mV to account for the steady-state depolarization of Golgi cells due to summation of long-lasting kainate receptor-mediated components of synaptic currents (Bureau et al., 2000) during ongoing activity *in vivo*. The intracellular resistivity was R_i = 150 Ω cm. The soma of each model neuron contained a generic action potential generation mechanism. When the somatic voltage threshold of –44 mV was reached in one of the neurons, a fixed action potential current waveform described by a difference between two probability density functions of skew-normal distributions and a decaying exponential was injected into the soma of that neuron. These current waveforms were pre-computed in Mathematica. The standard action potential current waveform was given by:

```
Table[2.5 PDF[SkewNormalDistribution[0.2, 0.2, 2], t] - 2.9
PDF[SkewNormalDistribution[0.7, 0.5, 2], t] - 0.01 Exp[-t/30.], {t, 0., 65.9, 0.1}]
```

and the action potential waveform with faster and smaller AHP was given by:

```
Table[2.5 PDF[SkewNormalDistribution[0.2, 0.2, 2], t] - 2.6
PDF[SkewNormalDistribution[0.7, 0.5, 2], t] - 0.01 Exp[-t/3.], {t, 0., 65.9, 0.1}]
```

where time t is given in ms, and current in nA.

The voltage waveform of the action potential was governed by the response of the compartmental model to the injected action potential current waveform. Accordingly, the voltage waveform was dependent on dendritic location, and was transmitted across the gap junction from the pre-junctional to the post-junctional neuron model,

arriving at the post-junctional soma as a DJP and HJP waveform. To separately test the influence of the DJP, HJP and the coupling of subthreshold membrane potential via the gap junction on the synchrony of spiking of the two neurons, the gap junction was operated in different modes (Fig. 6) in which it was switched on or off selectively during subthreshold signaling, the DJP and/or the HJP. The duration of the DJP at the pre-junctional end of the dendritic gap junction (1.65 ms) was used for gating the gap junction during the DJP, while gating for the HJP was performed for 180 ms, beginning at the end of the DJP at the pre-junctional end of the gap junction. The value of 180 ms was chosen such that the HJP had decayed to less than 0.1% of its peak amplitude at the end of the gating period.

Ongoing synaptic inputs *in vivo* were simulated by independent Poisson spike trains triggering synaptic conductances, which were distributed homogeneously per membrane area over the soma and dendrites of both neurons. In the case of 0% precisely synchronous common input, each synapse was driven by its own, independent Poisson spike generator, whereas in the case of x% precisely synchronous common input, x% of excitatory synapses and x% of inhibitory synapses on the pre-junctional neuron each received the same Poisson input spike trains as their counterparts on the post-junctional neuron. The time course of each synaptic conductance was described by a sum of two exponentials. Excitatory synaptic conductances had a peak amplitude of 0.25 nS, a rise time constant of 0.2 ms and a decay time constant of 1.0 ms. The excitatory reversal potential was 0 mV, and excitatory synapses were activated at a total rate (summed over all synapses on one neuron) of 240 Hz. Inhibitory synaptic conductances also had a peak amplitude of 0.25 nS, but a rise time constant of 0.5 ms and a decay time constant of 5 ms. The inhibitory reversal potential was –75 mV, and inhibitory synapses were activated at a total rate of 120 Hz per neuron. The time step of the simulation was 0.025 ms, and the simulated time for each cross-correlogram was 10,000 s. Cross-correlograms of simulated spike trains were calculated in the same way as cross-correlograms of experimentally measured spike trains.

Supplemental References

Bureau, I., Dieudonne, S., Coussen, F. & Mulle, C. Kainate receptor-mediated synaptic currents in cerebellar Golgi cells are not shaped by diffusion of glutamate. *Proc Natl Acad Sci USA* **97**, 6838-6843 (2000).

Deans, M.R., Gibson, J.R., Sellitto, C., Connors, B.W. & Paul, D.L. Synchronous activity of inhibitory networks in neocortex requires electrical synapses containing connexin36. *Neuron* **31**, 477-485 (2001).

Hines, M.L. & Carnevale, N.T. The NEURON simulation environment. *Neural Comput* **9**, 1179-1209 (1997).

Margrie, T.W.*, et al.* Targeted whole-cell recordings in the mammalian brain in vivo. *Neuron* **39**, 911-918 (2003).

Pernia-Andrade, A.J.*, et al.* A deconvolution-based method with high sensitivity and temporal resolution for detection of spontaneous synaptic currents in vitro and in vivo. *Biophys J* **103**, 1429-1439 (2012).

Simat, M., Ambrosetti, L., Lardi-Studler, B. & Fritschy, J.M. GABAergic synaptogenesis marks the onset of differentiation of basket and stellate cells in mouse cerebellum. *Eur J Neurosci* **26**, 2239-2256 (2007).

Vervaeke, K.*, et al.* Rapid desynchronization of an electrically coupled interneuron network with sparse excitatory synaptic input. *Neuron* **67**, 435-451 (2010).

Vervaeke, K., Lőrincz, A., Nusser, Z. & Silver, R.A. Gap junctions compensate for sublinear dendritic integration in an inhibitory network. *Science* **335**, 1624-1628 (2012).

Zeilhofer, H.U.*, et al.* Glycinergic neurons expressing enhanced green fluorescent protein in bacterial artificial chromosome transgenic mice. *J Comp Neurol* **482**, 123-141 (2005).
